# Immunogenic implications of translational readthrough modulate the association of *F8* nonsense mutations with inhibitors in Hemophilia A

**DOI:** 10.1186/s10020-026-01536-y

**Published:** 2026-06-27

**Authors:** Maria Francesca Testa, Mirko Pinotti, Alessio Branchini, Maria Elisa Mancuso, Francesco Bernardi, Dario Balestra

**Affiliations:** 1 Department of Life Sciences and Biotechnology and LTTA Center, University of Ferrara, Via Fossato di Mortara 74, Ferrara, 44121 Italy; 2 IRCCS, Humanitas Research Hospital, Center for Thrombosis and Hemorrhagic Diseases, Milan, Italy

**Keywords:** *F8* PTCs, Hemophilia A, Translational readthrough, Inhibitory antibody, Peptide MHCII affinity, Personalized medicine

## Abstract

**Background:**

Among *F8* mutation types, main determinants of inhibitor development after replacement therapy in Hemophilia A (HA), nonsense mutations display wide variation in the associated risk. Translational readthrough (Rdthr) at premature termination codons (PTCs) produces traces of full-length factor VIII (FVIII) including missense and wild-type molecules (WT Rdthr), and may influence the immune response involved in inhibitor formation.

**Methods:**

For inhibitor association analysis, we investigated *F8* genotypes, inhibitor status and Rdthr features in 335 PTCs (1048 patients), recorded in the European Association for Haemophilia and Allied Disorders (EAHAD) database, and exploited expression of *F8* PTCs variants. Mean-differences in affinity of HLA-DR alleles for FVIII WT peptides and their missense counterparts were bioinformatically calculated.

**Results:**

WT Rdthr was higher for patients affected by PTCs not associated with inhibitor and was not predicted at all in patients with PTCs detected in at least three inhibitor positive cases (*n* = 136, *p* = 0.0001). WT Rdthr was lower for PTCs in the inhibitor prone FVIII light chain.

Among FVIII PTCs fused with luciferase, quantitative output of WT Rdthr negative and positive groups did not differ, potentially highlighting for the positive group the importance of WT Rdthr to decrease inhibitor association. Readthrough output was the lowest among WT Rdthr negative PTCs, highly associated with inhibitors.

The WT Rdthr prediction was extended to all PTCs that could arise by single nucleotide variations for the entire *F8* coding sequence. Estimated WT Rdthr was higher in PTCs reported in EAHAD than those predicted (*n* = 662), foreseeing a higher risk of developing inhibitors.

In silico mean differences in affinity of HLA-DR alleles for WT FVIII peptides and their missense counterparts, potentially arising from Rdthr of PTCs without WT formation (*n* = 297), were higher for missense variants predicted in patients with inhibitors than without (*p* < 0.0001), and increased for PTCs present in more than one patient with inhibitor, potentially supporting immunogenic features.

**Conclusions:**

The new genetic classification of HA PTCs may improve our knowledge about their relationship with inhibitors. It deserves to be explored for estimating inhibitor PTC association in HLA genotyped patients as well as in other human diseases.

**Supplementary Information:**

The online version contains supplementary material available at https://doi.org/10.1186/s10020-026-01536-y.

## Background

**Fig. 1:**
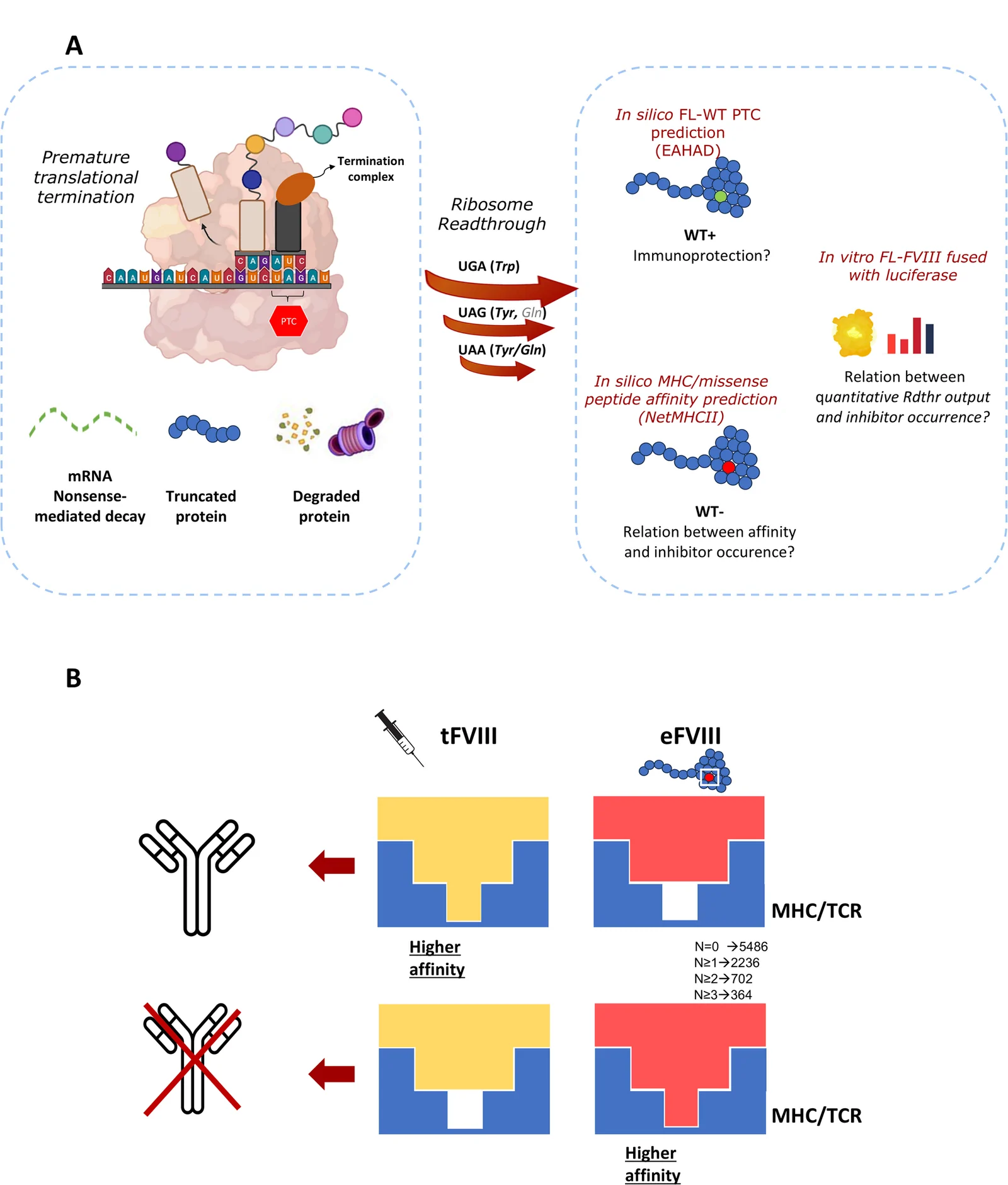
Schematic representation of the study. **A** Schematic representation of altered FVIII expression caused by *F8* premature termination codons (PTC) (Left panel) and investigation (Right panel) of readthrough features by in silico and in vitro approaches: estimation of WT Rdthr and of HLA affinity of missense peptides arising from ribosome readthrough and recombinant expression of FVIII nonsense variants fused with Gaussia luciferase. WT +, readthrough output with re-insertion of WT amino acid; WT-, readthrough output with re-insertion of missense variants only. **B** Immunogenic perspectives of FVIII peptides stemming from PTC readthrough (endogenous, eFVIII) and from therapeutic FVIII concentrate (tFVIII, NM_000132.4). TCR, T-cell receptor; MHC, Major Histocompatibility Complex; Trp, Tyr, Gln, amino acids most frequently inserted upon readthrough of UGA, UAG, UAA PTCs. The picture was prepared by using Biorender

Nonsense mutations, introducing premature termination codons (PTCs) into the mRNA sequence, result in the synthesis of truncated proteins with decreased stability and/or loss-of-function features (Fig. 1A), and in PTC-bearing transcripts which might trigger nonsense-mediated mRNA decay, (Popp and Maquat 2013) thus leading to the common classification as null variants (Lombardi et al. 2020).

One of the most relevant issues in patients affected by potentially null mutations treated with the infusion of the missing protein, such as Pompe (de Vries et al. 2017), Fabry disease (Matucci et al. 2026), mucopolysaccharidosis (Ghosh et al. 2019) and Hemophilias (Mancuso 2025; Mannucci 2023) is the development of inhibitory antibodies.

However, nonsense mutations result in variable risk of inhibitor development suggesting additional molecular genetic mechanisms playing a role (Singer et al. 2025; Testa et al. 2023).

Translational readthrough (Rdthr) (Fig. 1A), albeit with a low frequency (0.01–1% in mammalian cells) (Rospert et al. 2005), may potentially produce traces of full-length protein (Keeling et al. 2014) by overriding the PTC, through the incorporation of amino acid(s) subset predictable on the basis of PTC type (Blanchet et al. 2014). Among PTCs associated with inhibitor development after replacement therapy, Hemophilia A (Peyvandi et al. 2016) (HA, OMIM:306,700), whose mutations in the *F8* gene lead to coagulation factor VIII (FVIII) deficiency, represents a paradigmatic model to deepen our knowledge about relation of PTC Rdthr features and inhibitor susceptibility. HA displays a high prevalence of neutralizing antibodies against the infused FVIII (Ehrenforth et al. 1992; Lillicrap et al. 2020; Peyvandi et al. 2018) (30–35%) in severe patients (Paul et al. 2023) and, among multiple determinants (van Stam et al. 2025) of inhibitor risk, molecular genetics and epidemiological studies, and more recently machine learning approaches (Rawal et al. 2023), have clearly indicated the crucial role of *F8* mutation type (Schwaab et al. 1995) in patients with severe (Gouw et al. 2012) as well as non-severe (Eckhardt et al. 2013; Jourdy et al. 2018) HA. *F8* nonsense mutations display a highly variable inhibitor risk: in highly recurrent *F8* nonsense mutations, for which propensity to inhibitor onset can be affordably estimated, the inhibitor incidence (McVey et al. 2020) ranges from 7% (4 inhibitor patients out of 57) for the Arg814Ter, to 53% (26/49) for the Arg1985Ter, a 7–eightfold difference that has probably contributed to lower the performance of clinical risk prediction models (Hashemi et al. 2014; Hassan et al. 2024, 2021; Spena et al. 2018; ter Avest et al. 2008). Rdthr over a wide panel of recombinantly expressed variants supported that it contributes to secretion of traces of full-length FVIII protein, and to lower the B-domain PTC associated inhibitor risk.(Testa et al. 2023).

Based on strong experimental evidences (Jacquemin et al. 2003; James et al. 2011) aimed at interpreting the risk of inhibitor formation (Eckhardt et al. 2013), the *F8* missense variants have been considered alloreactivity models. They have been tested for the single amino acid difference between endogenous FVIII (eFVIII) and the administered therapeutic FVIII (tFVIII) protein (Fig. 1B) (Hart et al. 2018; Hassan et al. 2024; Schweiger et al. 2022). It has been established that the affinity between HA *F8* missense variants and MHC alleles (Li et al. 2025; Nielsen and Lund 2009) plays a role in inhibitor development (Hart 2020; Kempton and Payne 2018; Pashov et al. 2013; Shepherd et al. 2014). A higher affinity of the tFVIII, relative to the eFVIII, may increase the risk of triggering an immune response.

In this scenario, we hypothesized that translational Rdthr, potentially explaining the residual coagulation factor VIII expression observed in some patients with nonsense mutations (Branchini et al. 2016; Pinotti et al. 2012; Testa et al. 2023), could insert amino acids (WT or missense variants) influencing eFVIII immunological features. It is hypothesized that the rescue of the authentic FVIII protein, defined as wild-type (WT) Rdthr (Fig. 1A), could provide a protective effect on inhibitor development for tFVIII (Fig. 1B).

Here, we conducted an extensive analysis of genetic information from EAHAD (McVey et al. 2020) database (PTCs *n* = 335; 1048 patients), integrated with CDC Hemophilia Mutation Projects (CHAMP)(Payne et al. 2013), aimed at classifying nonsense mutations based on their propensity to Rdthr and inhibitor formation (Blanchet et al. 2014; Manuvakhova et al. 2000). PTC domain/chain localization was also studied because large FVIII chain–related difference in inhibitor was observed for this multi-domain protein circulating as heavy and light chains (HC, LC). These observations were integrated with Rdthr output analysis of *F8* PTCs *in-vitro* expression and in silico comparison of calculated affinity between FVIII missense peptides arising from Rdthr or FVIII WT with MHCII molecules.

The understanding of these relationships would be relevant to evaluate “in vivo” correlates of “in silico” predictions, potentially improving predictive models of inhibitor risk. Similar genotype-based, personalized approaches may be devised for others inherited diseases in which the development of neutralizing antibodies against the therapeutic infused protein has been observed (Berrier et al. 2015; Tsukimura et al. 2020). We provide new insights into the molecular genetics underpinnings of Rdthr, with potential implications in modulating tFVIII immunogenicity.

## Methods

### EAHAD database

The EAHAD genetic database (McVey et al. 2020) is a project to gather single gene variants related to inherited bleeding disorders, as hemophilia A and B, von Willebrand disease, and rarer coagulation disorders as Factor VII deficiency. At the time when the analysis was performed (6 April 2023), the database contained 3050 distinct unique *F8* mutations from 10,113 patients (den Dunnen et al. 2016; Goodeve et al. 2011). Of them, 335 nonsense variants with data from 1048 HA patients were reported (Supplementary Table 1). Among these, only 9 patients/PTCs were associated with moderate/mild hemophilia (Table 1).

**Table 1:** Main characteristics of patients with *F8* PTCs (EAHAD)

Characteristic	Patients (*n* = 1048)	PTCs (*n* = 335)
HA severity		
-Severe, n (%)	1039 (99)	326 (97)
-Moderate, n (%)	8 (0.7)	8 (2.3)
-Mild, n (%)	1 (0.1)	1 (0.3)
Inhibitor status		
-Positive, n (%)	231 (22)	33 (10)
-Negative, n (%)	817 (78)	302 (90)
*No, n (%)*	*594 (56.6)*	*229 (68.3)*
*Not reported, n (%)*	*223 (21.3)*	*106 (31.6)*
Predicted WT Rdthr		
-Positive, n (%)	195 (19)	105 (31)
-Negative, n (%)	853 (81)	230 (69)
Type of PTC		
-UGA, n (%)	613 (58)	88 (26)
-UAG, n (%)	243 (24)	120 (36)
-UAA, n (%)	192 (18)	125 (37)
PTC location		
-A1-A2 domain	286 (27)	106 (32)
-B domain	263 (25)	120 (36)
-A3-C1-C2	479 (46)	108 (32)

The inhibitor negative group includes patients for whom the absence of inhibitors is indicated (No), and those for whom no information is given in the database (Not reported). The degree of severity of patients was reported as severe (mostly with FVIII:C < 1%, as expected for PTC) unless reported as moderate/mild in the EAHAD database with indication of FVIII:C % levels. The number of PTCs includes the p.Arg15Ter PTC located in the FVIII signal peptide, removed in the secreted FVIII, and thus not considered in the domain analyses

*PTC* Premature termination codon, *HA* Hemophilia A, *WT* Wild-type

To mitigate the potentially confounding effect of sporadic inhibitor occurrence associated with recognized and treatment-related risk factors (e.g. intensity of FVIII infusions, type of FVIII concentrate used, etc.) (Jardim et al. 2020), and thus better evaluate the *F8* genetic propensity to develop neutralizing antibodies, we evaluated PTCs present in multiple inhibitor positive cases. For this purpose, we grouped PTCs and patients (Pts) according to four categorization criteria: PTCs for which inhibitor was absent or not reported in the EAHAD database were defined as PTC^inh=0^, and PTCs for which inhibitor was reported in at least 1, 2 or 3 patients were defined as PTC^inh≥1^, PTC^inh≥2^ or PTC^inh≥3^, respectively. Accordingly, patients affected by PTCs for which inhibitor was absent or not reported were defined as Pts^inh=0^, and Pts^inh≥1^, Pts^inh≥2^ or Pts^inh≥3^ were those with PTCs found in at least 1, 2 or 3 patients with inhibitor.

### Other databases

To integrate data from databases, particularly on PTC^inh=0^, we referred also to CHAMP database categorization, with the limitation that the number of patients with inhibitors for each PTC is not reported in this database. The discrepancy between EAHAD and CHAMP concerning the inhibitor status (defined “Yes” or “No”) was implemented with CHAMP data, leading to switch from PTC^inh=0^ to PTC^inh≥1^ classification for two PTCs (Ser872Ter UAG and UAA). A cumulative count of EAHAD and CHAMP patients, which would require patients’ identification in both databases, was not feasible. Data in the My Life Our Future MLOF and TopMed databases could not be analyzed due to accessibility limitation.

### Wild-type ribosome readthrough prediction

Nonsense variants can be further classified by the potential Rdthr‐induced re-insertion of the wild‐type residue or not (Fig. 1A, Supplementary Table 1) based on the reported observation that glutamine and tyrosine are preferentially (44% and 54% respectively) inserted at UAA, and tryptophan (82%) at UGA (Blanchet et al. 2014; Roy et al. 2015). For UAG, the hypotheses that tyrosine or glutamine may be alternatively inserted, based on different experimental settings (Blanchet et al. 2014; Roy et al. 2015), were both explored. We also explored the association of lysine incorporated at low frequency at UAA (2%), cysteine and arginine at UGA (14% and 4% respectively) and lysine at UAG (3%).

### Recombinant expression in PTCs with or without WT readthrough.

Quantitative readthrough output in relation to the predicted presence of WT Rdthr (although without identification of the incorporated amino acid) was investigated by evaluation of recombinant *F8* cDNA bearing PTCs plasmid expression in mammalian cells, previously performed by us by FVIII*-Gaussia* luciferase fusion (Testa et al. 2023). For this purpose luciferase activity was measured in PTCs (dedicated column in Supplementary Table 1) either positive (*n* = 12) or negative (*n* = 33) for WT Rdthr, the last further subgrouped for PTCs^inh=0^ (*n* = 12), PTCs^inh≥1^ (*n* = 21), PTCs^inh≥2^ (*n* = 17) and PTCs^inh≥3^ (*n* = 12). The comparison between groups was conducted by unpaired t test with two tails. The results were considered statistically significant with *p*-values ≤ 0.05.

### Peptide-MHC binding prediction

To test the possibility of generating a novel peptide MHC (p-MHC) surface, our workflow started by exploiting the widely used in silico NetMHCII tool (Nielsen and Lund 2009) to predict strength of peptide-MHC binding. Starting from a 15-residue long peptide, the NetMHCII uses an artificial neural network to predict the core set of nine consecutive residues within this peptide that bind optimally within the groove of the MHC class II (MHC-II) molecule and the avidity with which that 9-residue core binds within the MHC groove. To evaluate the strength of binding, we used the metric of percentile rank providing a uniform scale that allows comparisons across different HLA alleles. A percentile rank is generated by comparing the selected peptide's predicted binding affinity with that of a large set of similarly sized peptides randomly selected from the SWISS-PROT database, with values ranging between 0 and 100, where 0 corresponds to a high binding affinity for a given HLA allele and 100 to a low binding affinity (Pashov et al. 2013).

The use of percentile ranking instead of measured affinity may improve comparison by decreasing allele-specific bias (Jurtz et al. 2017; Reynisson et al. 2020).

For the analysis, a set of all 29 mers spanning the position of any FVIII missense-derived variants (14 mers before and after the target variant) from eFVIII, predicted to arise from Rdthr of PTCs, and corresponding mers from tFVIII, were submitted to NetMHCII to predict the 9-residue core and how strongly each binds to the MHC groove.

26 HLA class II alleles that are most frequent in the general worldwide population were considered during the prediction. Variants for which it was impossible to extrapolate a 29-mer due to their location to the C-terminus of the FVIII sequence were removed from the analysis. By taking into consideration the most probable amino acid incorporation upon Rdthr of the three PTC types, analysis of p-MHC binding resulted in 252.330 predicted percentile ranks (647 29-mers × 26 alleles × 15-mer predictions).

For each allele, only the epitope with the minimum percentile rank was considered, and the difference with the tFVIII calculated.

### Principal Component Analysis (PCA) analysis

Principal Component Analysis (PCA) was performed to explore the variability among PTCs reported in the EAHAD database. The analysis included the following variables: FVIII protein chain, PTC class, the identity of the fourth nucleotide downstream of the stop codon, the PTCs exon location within the *F8* mRNA sequence, and the percentage of WT Rdthr observed. Categorical variables were appropriately encoded to allow integration with continuous features. The PCA was carried out using the Orange data mining software (v.3.38), and the first two principal components were visualized to assess the distribution of PTCs across functional and structural contexts.

### Statistical analysis

The comparison between groups has been conducted by Fisher's exact test with two tails.

The results were considered statistically significant with p-values ≤ 0.05. Given the exploratory nature of the study, which includes multiple subgroup analyses, we did not apply a formal correction (such as Bonferroni or Benjamini-Hochberg) to all comparisons in the main analyses. Grubb’s test was performed to identify statistical outlier.

## Results

### PTCs predicted to undergo Wild-type readthrough are associated with a lower incidence of inhibitors

To investigate differential association between the PTC reported in the EAHAD database and inhibitor development, at first, we analyzed the distribution of patients with and without inhibitors respect to the predicted WT Rdthr.

Table 1 summarizes the main characteristics of the patients’ population in relation to the present study, and Supplementary Table S1 provides details on single PTCs.

To mitigate the potentially confounding effect of sporadic inhibitor occurrence associated with recognized risk factors (e.g. intensity of FVIII infusions, type of FVIII concentrate used, etc. (Jardim et al. 2020)), and thus better evaluate the *F8* genetic propensity to develop neutralizing antibodies, we evaluated PTCs present in multiple inhibitor positive cases.

**Fig. 2:**
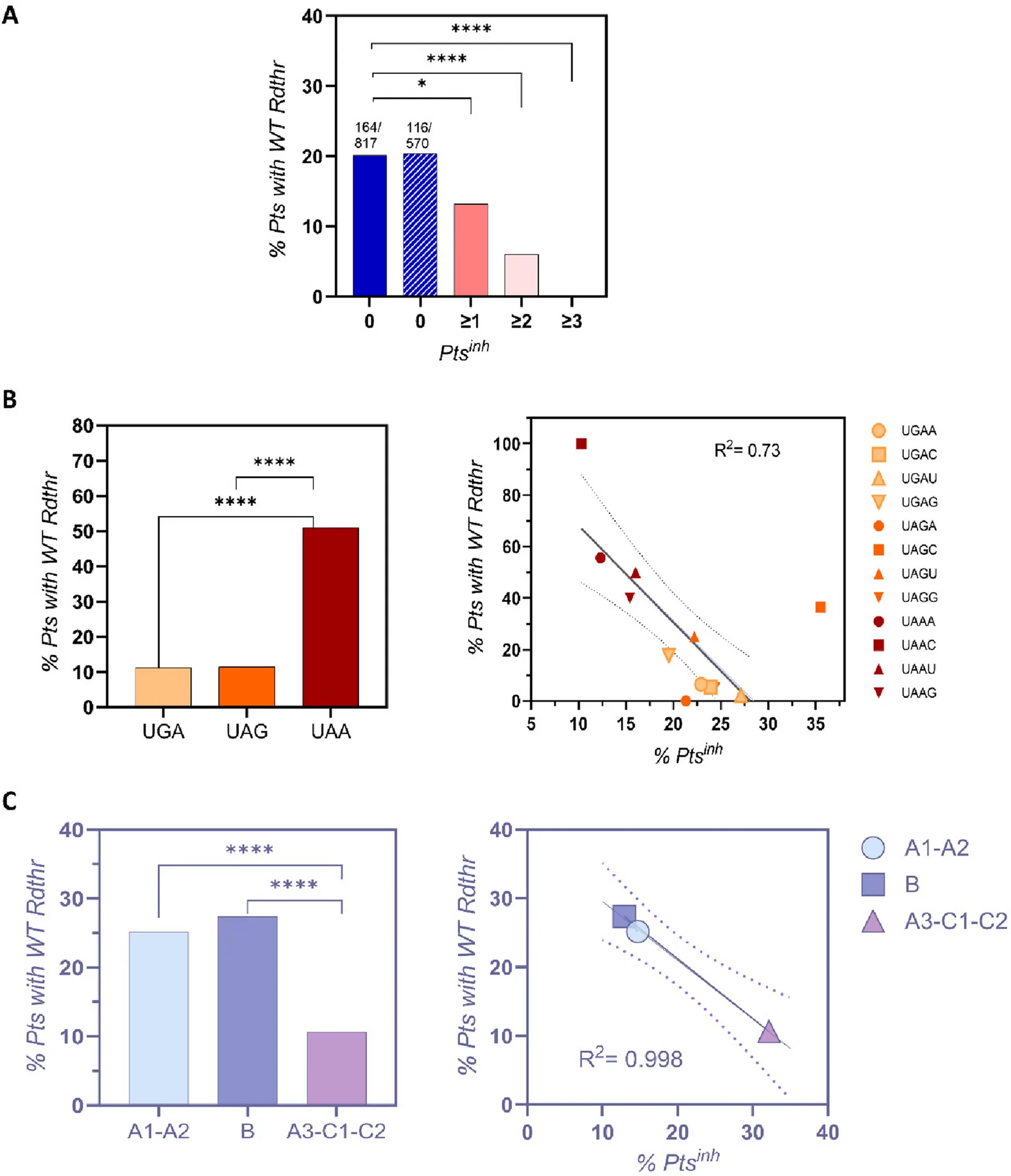
Insertion of WT amino acid upon readthrough is associated with inhibitor incidence in patients. **A** HA patients with *F8* PTCs predicted to undergo WT readthrough were classified in accordance with PTCs association with an increasing number of inhibitor positive cases. Patients affected by PTCs for which inhibitor was absent or not reported were defined as Pts^inh=0^ (blue bar), and Pts^inh≥1^, Pts^inh≥2^ or Pts^inh≥3^ were those with PTCs found in at least 1, 2 or 3 patients with inhibitor. Patients affected by PTCs for which inhibitor was absent (Pts^inh=0^) were represented by hatched blue bar. **B** *Left panel.* Distribution of patients grouped for PTC classes in terms of predicted WT readthrough (WT Rdthr). *Right panel*. Relation between predicted WT Rdthr and inhibitor occurrence for each PTC (UAA/UAG/UGA) and downstream nucleotide (A/C/G/U) representing all 12 tetranucleotide combinations (colored symbols). See also supplementary Table S7 and S8. The R^2^ for linear correlation is reported with the confidence limits of the distribution, excluding UGAC value. **C** *Left panel*. Distribution of patients with WT Rdthr as a function of grouped FVIII domains location of PTCs. *Right panel*. Relation between patients with PTCs predicted to undergo WT Rdthr, and patients with inhibitors, considering PTCs location in the FVIII domains. The R^2^ for linear correlation is reported with the confidence limits of the distribution

The comparison among patient groups (see Methods for details) revealed that (Fig. 2A, and Supplementary Table S2A) the Pts^inh=0^ group was characterized by a percentage of WT Rdthr higher than Pts^inh≥1^ (164/817, 20.1% vs 31/231, 13.4%; *p* = 0.022). The percentage of WT Rdthr further decreased in Pts^inh≥2^ (10/166, 6%; *p* = 0.0001) and was not predicted at all in Pts^inh≥3^ (0/136; *p* = 0.0001).

Noticeably, virtually overlapping percentages of WT Rdthr were observed including in the group of Pts^inh=0^ only patients for whom the absence of inhibitor was reported in the database (116/570, 20.4%) (Fig. 2A, hatched blue bar and Supplementary Table S2B). Furthermore, the large statistical differences between Pts^inh=0^ and inhibitor patients’ groups were also maintained.

We performed an additional analysis considering the reinsertion at low frequency of cysteine and arginine at UGA and lysine at UAA and UGA PTCs. This simulation produces an exceeding number of patients with PTCs potentially undergoing low frequency WT Rdthr (633/1048, 60.4%) and, among these, virtually all Pts^inh≥3^ (133/136, 97.8%). These numbers do not suggest that reinsertion of WT amino acids at very low frequency contributes to modulating PTCs inhibitors association.

Based on partially discrepant results in previous studies, the procedure was repeated by predicting main UAG Rdthr as Gln (Roy et al. 2015) instead of Tyr (Blanchet et al. 2014), with UAA and UGA maintaining the Tyr/Gln and Trp codification, respectively (Supplementary Figure S1A and Supplementary Table S3). As observed for the UAG Rdthr as Tyr (Fig. 2A), the proportion of WT Rdthr was higher in patients without inhibitors than in Pts^inh≥1^ (209/817, 25.6% vs 46/231, 19.9%; *p* = 0.082), and the percentage of WT Rdthr progressively decreased in Pts^inh≥1^, Pts^inh≥2^ and Pts^inh≥3^. However, the Tyr model was better associated than the Gln model (i.e. 2 orders of magnitude lower p-value for Pts^inh=0^ vs Pts^inh≥2^ and 4 orders of magnitude lower p-value for Pts^inh=0^ vs Pts^inh≥3^, Supplementary Figure S1B) with the inverse relation between the hypothesized presence of WT Rdthr and inhibitor occurrence. This comparison prompted us to conduct the subsequent analyses considering Tyr as main UAG Rdthr prediction.

With the limitation of differences in database reporting the inhibitors status (see Methods paragraph, 

“EAHAD database”), we then attempted to integrate information available in the EAHAD and CHAMP databases. Considering that the CHAMP database does not report the number of patients with inhibitor for each PTC, we only compared the Pts^inh=0^ vs Pts^inh≥1^ groups (Supplementary Table S4). The percentage of WT Rdthr (158/807, 19.7% vs 35/241, 14.5%, p = 0.07) was similar to that observed in the EAHAD database only (164/817, 20.1% vs 31/231, 13.4%; p = 0.022).

To detail the different association between PTCs and inhibitor occurrence, the distribution of patients for each PTC class (UGA, UAG, UAA) with respect to antibody development was investigated (Supplementary Table S5). UAA PTC carriers displayed inhibitory antibodies less frequently (26/192, 13.5%) than UAG (59/243, 24.3%; *p* = 0.05) or UGA (146/613, 23.8%; *p* = 0.002). Next, we examined whether differences existed among patients grouped for the PTC class in terms of predicted WT Rdthr (Fig. 2B left panel and Supplementary Table S6). The UAA PTC class, associated with the lowest incidence of patients with inhibitors, showed by far the highest proportion (98/192, 51.0%) of patients with PTCs potentially undergoing WT Rdthr compared with UGA (69/613, 11.2%; *p* < 0.0001) and UAG (28/243, 11.5%; *p* < 0.0001).

Prompted by these data, the relationship between the number of patients with inhibitors and patients with predicted WT Rdthr was investigated taking into account PTC class and the fourth nucleotide, a well-known molecular determinant of Rdthr efficiency (Manuvakhova et al. 2000). Despite the moderate number of patients in each group (Supplementary Table S7 and S8), 11 tetranucleotides (Fig. 2B, right panel) displayed a tight and inverse relationship (R^2^ = 0.73) between percentage of WT Rdthr and percentage of patients with inhibitors. The outlier UAGC (Grubb’s test P = 0.012) was characterized by the highest value of patients with inhibitors.

To investigate the previously observed lower incidence of inhibitor occurrence for PTCs located in the heavy chain (Gouw et al. 2012; Singer et al. 2025), WT Rdthr was compared in heavy chain and light chain domains. The FVIII A3-C1-C2 domains were associated (Supplementary Figure S2, Supplementary Table S1 and Supplementary Table S9) with a two-fold increase in inhibitor positive patients (157/479, 32.1%) as compared with A1-A2 (42/286, 14.7%) and B (34/263, 12.9%) domains. This difference remained considering each PTC class (Supplementary Figure S3). The percentage of Pts^inh^ with UAA PTCs in the A3-C1-C2 domains (30.9%) was 3.5-fold higher than in the A1-A2 and B domains (8.7% and 8.6%, respectively), and approximately two-fold higher for UAG (A3-C1-C2, 34.3% vs A1-A2, 17.9% and B domains, 15.9%) and UGA (A3-C1-C2, 31.6% vs A1-A2, 14.7% and B domains, 12.9%). The WT Rdthr was significantly less represented in patients with PTCs in the A3-C1-C2 (51/479, 12%) than in the A1-A2 (72/286, 25.2%) and B (72/263, 27.4%) domains (Fig. 2C left panel and Supplementary Table S10), as well as comparing separately A3, C1 and C2 domains (Supplementary Figure S4). In these domains, the distributions of patients with WT Rdthr and with inhibitors were inversely related (Fig. 2C, right panel).

This comparison of inhibitor occurrence between domains/chains does not include the underlying effects of nonsense-mediated mRNA decay, that may modulate endogenous antigen availability (David et al. 2003) and thus immune tolerance.

**Fig. 3:**
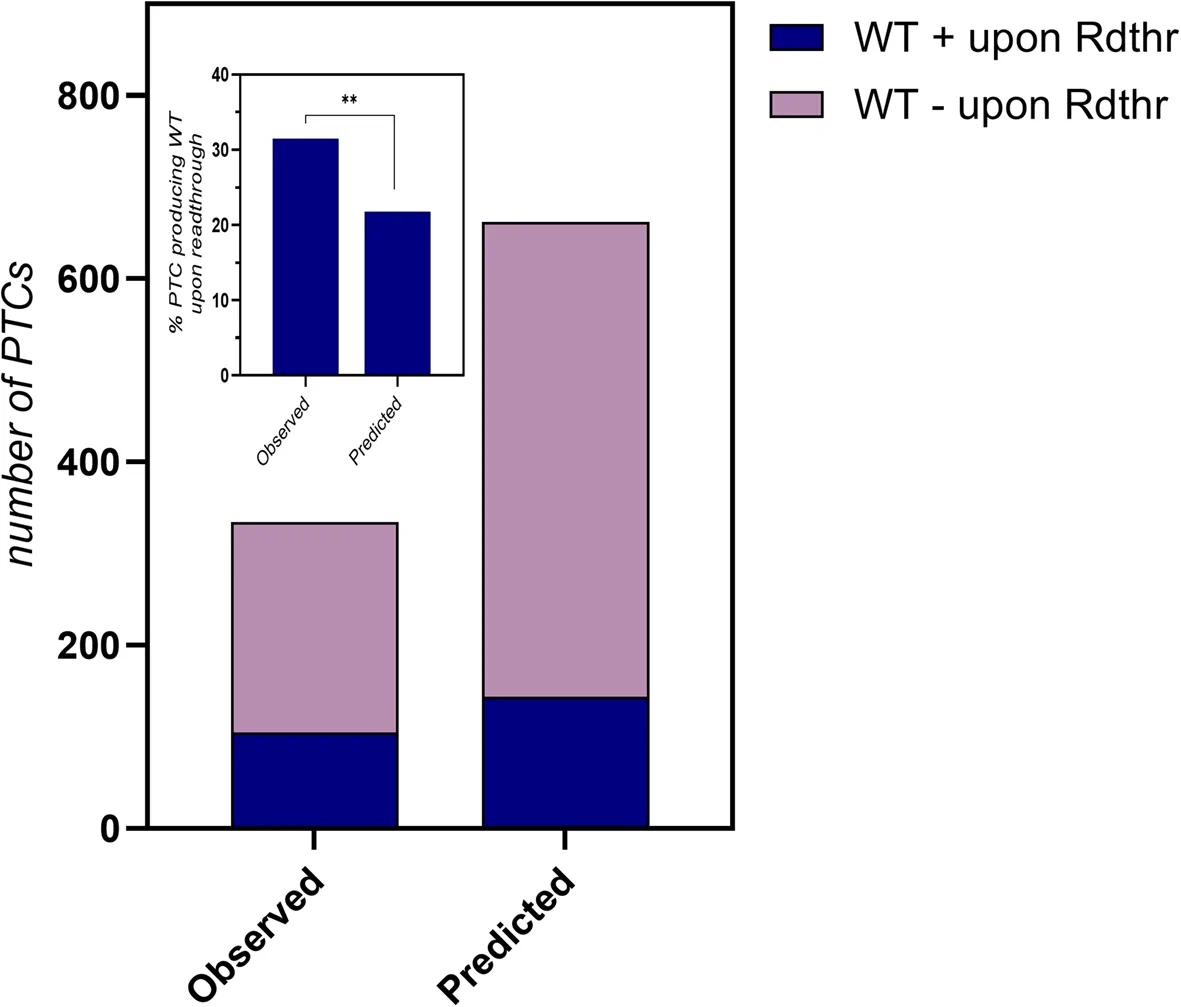
WT readthrough in observed and predicted PTCs. Number of PTCs producing WT FVIII upon Rdthr (blue bars) or not (dark pink bars) among PTCs observed (reported in EAHAD database) and predicted (never reported before) by Single Nucleotide Variations in the entire *F8* coding sequence. Inset: Comparison between percentage of WT Rdthr among observed and predicted PTCs

The WT Rdthr prediction was extended to all PTCs that could arise by single nucleotide variations (SNV) for the entire *F8* coding sequence (Fig. 3). PTCs never reported, defined as “predicted” were approximately twice (*n* = 662) of those listed in the database, defined as “observed” (*n* = 335). This analysis (Fig. 3 and Supplementary Table S11) indicates that “predicted” PTCs are characterized by a significantly lower proportion of WT Rdthr than “observed” PTCs (144/662, 22% vs 105/335, 31%; inset Fig. 3, p = 0.001). Observed and predicted PTCs did not differ in PTC class distribution (Supplementary Figure S5 and Supplementary Table S12).

We performed a PCA analysis taking into consideration as variables FVIII domains, PTC class and the fourth nucleotide, the percentage of WT Rdthr and *F8* PTC exon location (PCA loading are listed in Supplementary Table 13), which may predict Nonsense-Mediated Decay occurrence. This PCA analysis of PTCs in the EAHAD database visually represents (Supplementary Figure S6) the variability of PTCs, and partially highlights minor sub-groups not associated with inhibitor in the inhibitor-prone C1-C2, or associated with inhibitor in the A1-A2 and B domains.

### Recombinant expression levels of FVIII PTC WT Rdthr negative variants support the inverse relation with inhibitor occurrence

Differences in Rdth output between PTC WT Rdthr + and PTC WT Rdthr – were investigated through *F8* cDNA bearing PTCs fused with secretable Gaussia luciferase sequence to evaluate FVIII full-length (FL) expression (Testa et al. 2023). The PTCs positive (*n* = 12) or negative (*n* = 33) to WT Rdthr, spread on different domains, overall are present in 579 HA patients, among whom 147 with inhibitor (Supplementary Table S1). FL-FVIII from PTCs WT Rdthr + would include a mixture of FL FVIII WT and missense variants and, FL-FVIII from PTCs WT Rdthr – would include only missense variants.

**Fig. 4:**
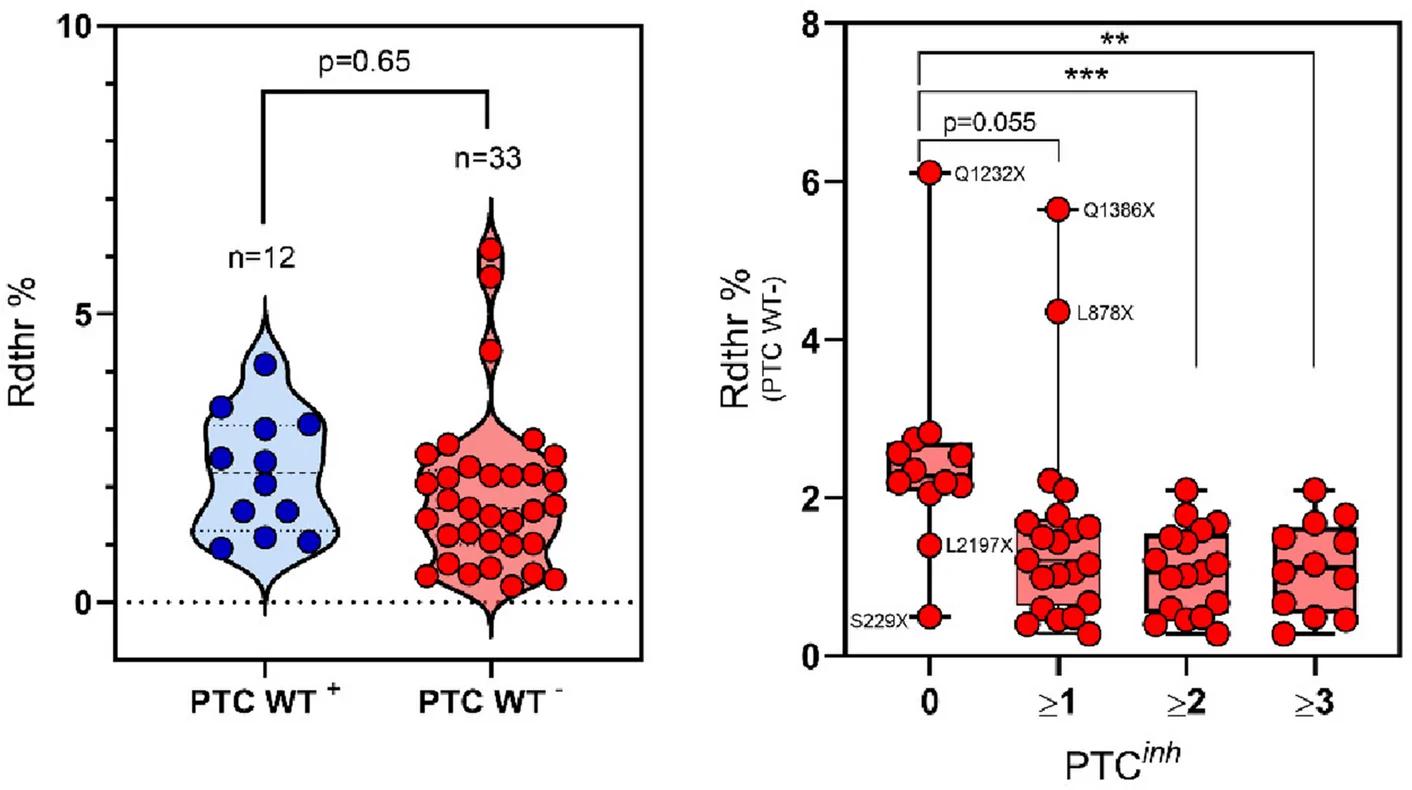
In vitro expression of FL-FVIII Gaussia luciferase in WT + and WT – PTCs. *Left panel*. Comparison of expression from PTC variants predicted to undergo WT Rdthr (WT +) or not (WT-); *Right panel*. Expression from PTC WT- variants according to their association with inhibitors; PTC^inh=0^, not associated with inhibitors; PTC^inh≥1^, PTC^inh≥2^, PTC^inh≥3^, PTCs for which inhibitor was reported in at least 1, 2 or 3 patients. Rdthr over WT + PTCs is expected to produce a mixture of FL-FVIII with reinsertion of WT amino acid and FL-FVIII missense variants, whereas that over WT – PTCs only FL-FVIII missense variants

Quantitative differences in Rdthr output were not detected between PTC groups (Fig. 4, PTC WT +, 2.24 ± 1.02% FVIII-Gaussia luciferase; PTC WT -, 1.92 ± 1.36%, *p* = 0.65). This analysis suggests that qualitative differences in Rdthr output, namely the predicted presence of FL-FVIII WT, would preferentially contribute to the inverse relation with the inhibitor occurrence in patients (Fig. 2A).

Although the Gaussia-luciferase assay quantifies translational readthrough but does not identify the incorporated amino acid, we predicted among recombinants, the WT Rdthr – PTCs. Among WT Rdthr – PTCs, higher expression of FL-FVIII in the PTC^inh=0^ (mean 2.4 ± 0.38% FVIII-Gaussia luciferase) than in the PTCs^inh≥1–3^ groups (mean 1.53 ± 1.29%, 1.08 ± 0.54% and 1.13 ± 0.58%) was observed, albeit with wide variability in the PTC^inh=0^ and PTCs^inh≥1^ groups (Fig. 4). Furthermore, in a subgroup of recurrent WT Rdthr – PTCs, not located in the B domain because it is more prone to productive Rdthr than other domains (Testa et al. 2023), an inverse correlation between Rdthr output and the ratio between inhibitor positive/total patient number was suggested (Supplementary Figure S7). These recombinant data in predicted WT Rdthr – PTCs indirectly agree with the seminal notion (Gouw et al. 2013) that higher amounts of endogenous FVIII antigen are associated with a lower risk of inhibitor development.

### The differential affinity between HLA-DRB alleles and tFVIII or eFVIII readthrough-derived missense variants contributes to explaining the PTCs inhibitor association

**Fig. 5:**
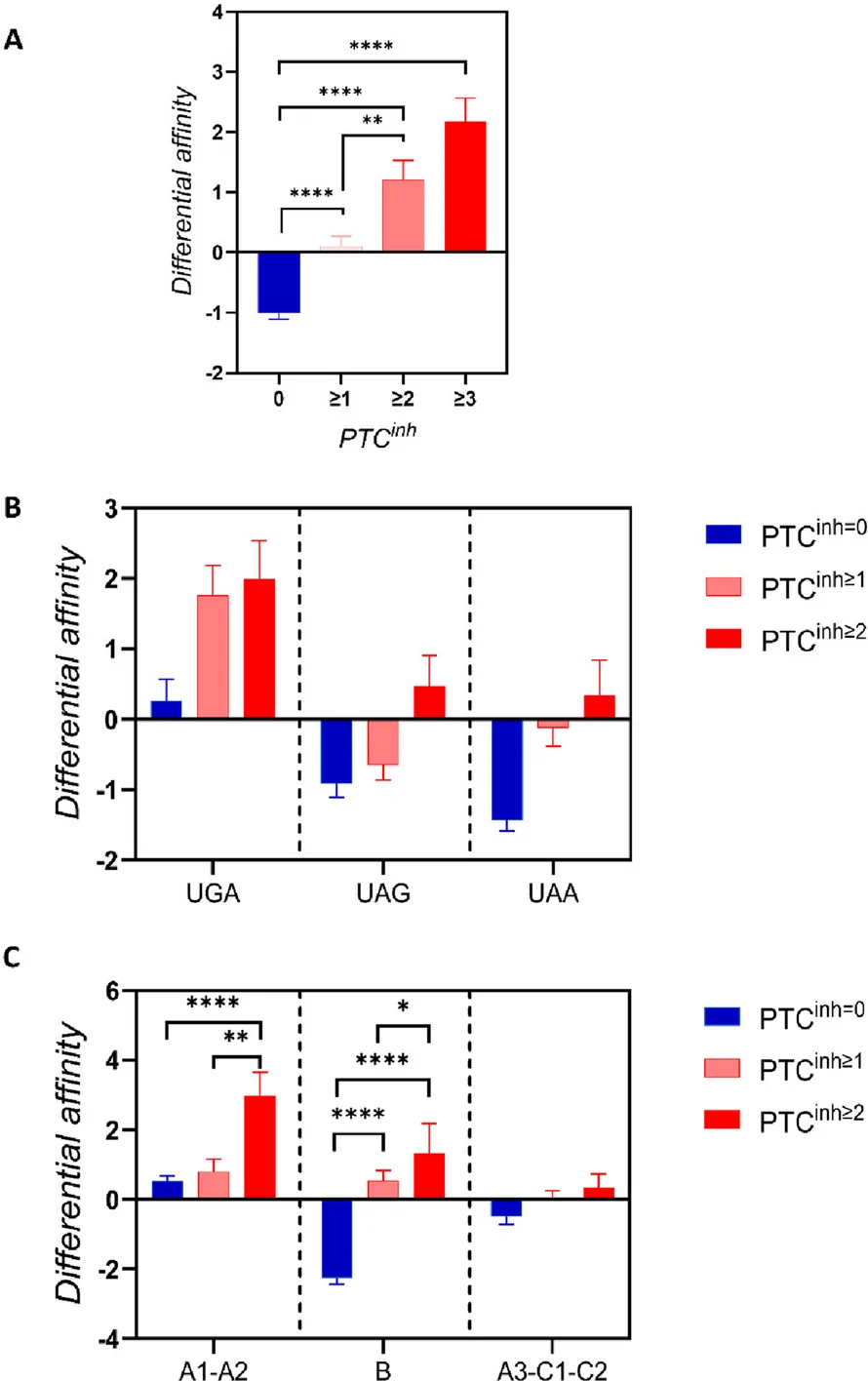
Differential affinity between missense readthrough FVIII peptides and HLA-DRB alleles. **A** Mean differential affinity predicted between FVIII peptides bearing the amino acid substitutions expected to arise from WT Rdthr - PTCs (*n* = 297) and the most prevalent HLA-DRB alleles (*n* = 26). A total of 7,722 calculated affinities were compared with those of the WT FVIII sequence (tFVIII) at the same position. The analysis was conducted by percentile ranking to improve comparison. The negative values observed for PTCs found in patients without inhibitors (PTC^inh=0^) increased in inhibitor positive PTCs (PTC^inh≥1^), and in those detected in two or more patients (PTC^inh≥2^) or three or more patients (PTC^inh≥3^); **B** comparison of differential affinity in PTCs classes (UGA, UAG, UAA) and (**C**) FVIII domains (A1-A2 domains; A3-C1-C2 domains; B-domain). Values do not include the p.Arg15Ter PTC located in the FVIII signal peptide and thus not considered in the peptide affinity analyses

Higher affinity of the administered tFVIII to HLA-DRB alleles as compared with that of the eFVIII counterpart may increase the risk of immune response (Fig. 1B). Based on this premise, we calculated, for PTCs not expected to undergo WT Rdthr (*n* = 297 from 227 triplets), the predicted affinity between the most prevalent HLA-DRB alleles (*n* = 26) (Greenbaum et al. 2011), and FVIII peptides bearing the amino acid substitutions arising from Rdthr. The resulting values, a total of 7,722 calculated affinities, were compared with the affinity of the WT tFVIII sequence at the same position (Fig. 5). To this purpose, the mean difference of the affinity between tFVIII and predicted missense variants (eFVIII) were analyzed by percentile ranking to improve comparison (Reynisson et al. 2020). Largely negative values were observed for PTCs in patients without inhibitors (PTC^inh=0^), which progressively increased for variants originating from inhibitor positive PTCs (PTC^inh≥1^), in those detected in two or more patients (PTC^inh≥2^) or three or more patients (PTC^inh≥3^) (Fig. 5A). Differences were maintained after correction of affinity values for HLA allele frequencies (data not shown).

Noticeable differences among PTC classes (Fig. 5B), leading to insertion of different residues in the FVIII peptides, contribute to these patterns. For all PTC classes the differential mean affinities of Rdthr-derived missense variants increased with the number of inhibitor positive patients. Peptides stemming from UGA contributed to the largely positive affinity values associated with inhibitor onset, and those from UAA to those largely negative associated with absence of inhibitors. Differently, peptides stemming from UAG contributed to negative affinity values both in the absence and presence (PTC^inh≥1^) of inhibitors.

We further explored the predicted differential affinity based on FVIII domains patterns (Fig. 5C and Supplementary Table S14). In the A3-C1-C2 domains, modest mean differences in affinity were observed among PTCs found in patients with or without inhibitors. In the A1-A2 domains, positive differences significantly increased for PTCs associated with inhibitors. In the B domain, differences were negative for PTCs not associated with inhibitors, and significantly increased with positive values for PTCs associated with inhibitors. All FVIII domains showed an increased differential affinity for PTCs associated with inhibitors, and the extent of increase from PTC^inh=0^ to PTC^inh≥2^ was lower for A3-C1-C2 (0.82) than A1-A2 (2.47) and B (3.57) domains (Supplementary Table S14).

These domain-dependent differences in affinity were detailed in relation to the PTC classes. The UAA PTCs, associated with largely negative affinity (Fig. 5B), were more represented in the B domain, displaying the most negative affinity values for PTCs not associated with inhibitor (Fig. 5C). Whereas for the UGA codon the mean differential affinity clearly differentiates, with positive values, from inhibitor negative (PTC^inh=0^) to inhibitor positive (both PTC^inh≥1^ and PTC^inh≥2^) PTC, for the UAG codon negative and positive values only differentiates from PTC^inh=0^ to PTC^inh≥2^.

**Fig. 6:**
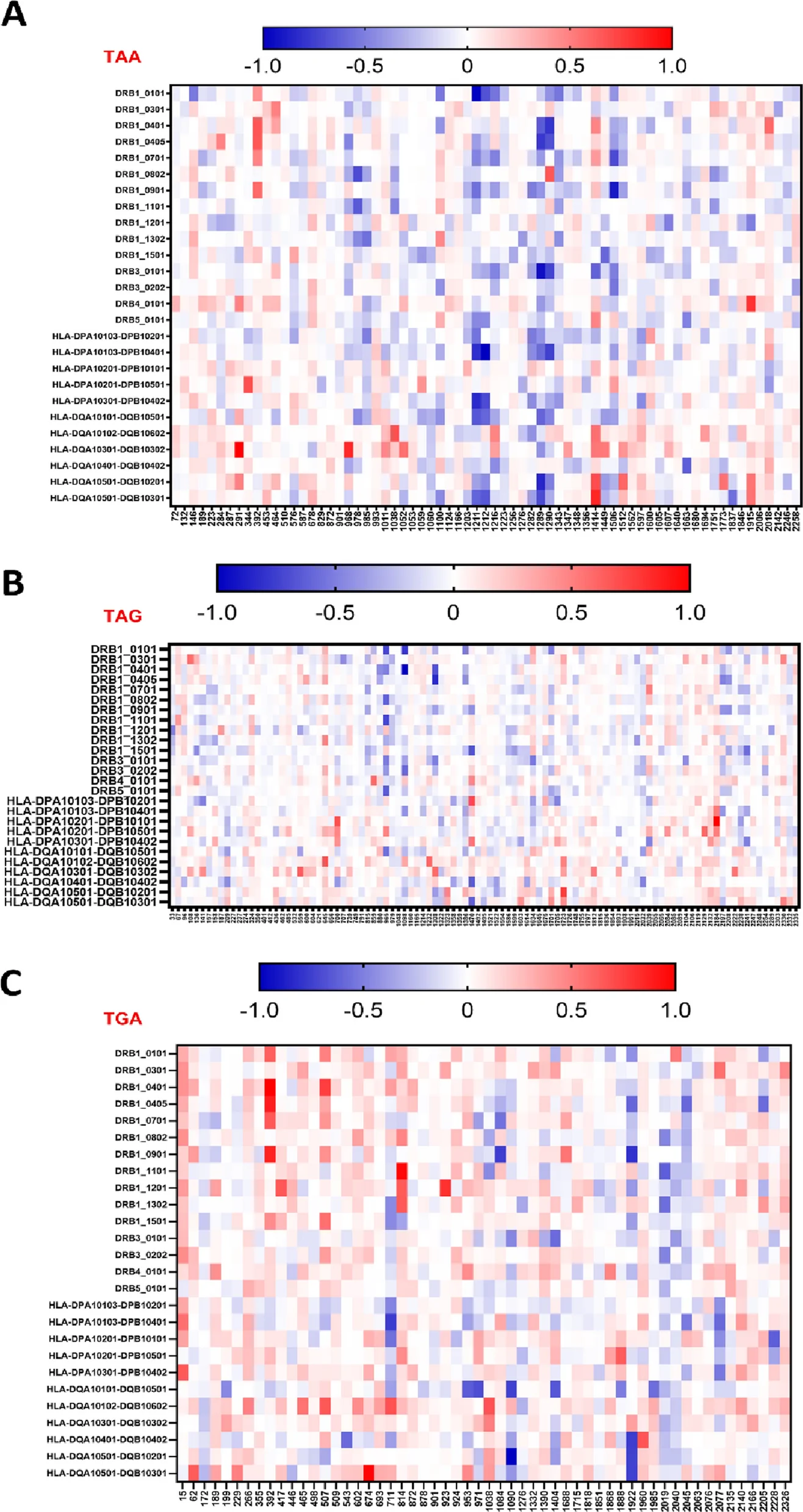
Heatmap of differential affinity between MHCII alleles and FVIII readthrough missense peptides. The TAA, TAG, TGA stop triplets are separately numbered along the X-axis in Fig. 6 A, B and C. The Y-axis lists the 26 HLA-DRB alleles. Only PTCs with no predicted WT Rdthr were included in the analysis. All non-zero differential affinity values were scaled to the range from –1 to + 1 to facilitate comparison across variants and alleles. The red color range indicates high differential peptide affinity (PTCs potentially associated with inhibitors). The blue color range indicates low differential peptide affinity (PTCs potentially not associated with inhibitors). For further details refer to Supplementary Table S15_sheet2B

The differential affinity between 26 HLA-DRB alleles and 297 PTCs is represented as heatmap (Fig. 6), which visually illustrates the extreme heterogeneity of combinations between affinity of peptides predicted by missense readthrough of *F8 PTCs* and DRB/HLA alleles.

Supplementary Table S15 includes observed and predicted PTCs with (sheet 1) and without (sheet 2A-B) WT Rdthr, respectively. Sheet 2B reports the differential HLA-DRB allele-specific affinity for missense variants arising from observed (EAHAD) and predicted PTCs without WT Rdthr, to be explored for specific combinations in genotyped patients.

## Discussion

Premature termination codons (PTCs) are commonly associated with “null” phenotypes. However, molecular genetics mechanisms, such as translational readthrough may produce traces of full‑length WT or missense proteins, rendering PTC as a continuum in protein levels and “immunological self”. This in turn may modulate the propensity to develop neutralizing antibodies when the missing antigen is restored by protein replacement therapy or genetic medicines. Genotypes of patients affected by Hemophilia A, Pompe and classic Fabry diseases indicate that true “null” alleles can predispose to clinically meaningful inhibitors development against infused proteins.

Based on several decades of genetic and phenotype investigation, Hemophilia A offers an ideal framework to test relationship among *F8* mutation type, ribosome readthrough and inhibitor development. Whereas extensive *F8* gene deletions have been identified as strong inhibitor risk predictors, PTCs displayed a wide variation, supporting the main hypothesis of this study by in vitro and in vivo observations (Testa et al. 2023),(Branchini et al. 2016). Furthermore, large-scale comparison of predicted antigen presentation between the endogenous peptides, spanning all *F8* missense variants, and therapeutic FVIII-derived peptides have clearly indicated the importance of interpreting *F8* genotype in the context of individual HLA profiles (Eckhardt et al. 2013; Hart 2020; Miranda et al. 2024; Pashov et al. 2013; Shepherd et al. 2014). Although direct evidence for the presence in patients’ plasma of endogenous WT FVIII peptides is not provided, predicting re-insertion by Rdthr of WT residues represents an original contribution of this study. Patients with inhibitors were characterized by a lower proportion of PTCs permitting WT Rdthr as compared with patients without inhibitor. Noticeably, for PTCs detected in at least three patients with inhibitor the presence of WT Rdthr was not predicted, which underlines a highly significant *F8* genetic association that interacts with other genetic and acquired components.

With the limitation that the Gaussia-luciferase assay quantifies translational Rdthr but does not identify the incorporated amino acid, recombinant data suggest that the contribution of traces of WT FVIII, predicted as a qualitative feature of PTCs WT Rdthr +, may not depend on higher quantitative expression. Differently, higher amounts of missense FVIII peptides supports the hypothesis to lower inhibitor risk for PTCs WT Rdthr +. While waiting for experimental validation of our observations, dichotomic prediction of WT Rdthr + and WT Rdthr—PTCs could be explored to improve clinical risk prediction models.

A second original contribution of our study consists in the “in silico” analysis of affinity between around 300 *F8* missense variants, expected to arise from Rdthr of > 200 PTCs without prediction of WT Rdthr, and 26 HLA alleles covering more than 98% of worldwide population (Greenbaum et al. 2011). Although these predictions estimate binding affinity and only indirectly suggest immunogenicity, the differential affinity of missense peptides was substantially higher for mutations found in patients with inhibitors than in those without. Of note, differences progressively became more pronounced for mutations associated with a higher number of inhibitor positive patients. This strongly supports combination of Rdthr-derived missense variants and MHC affinity predictions to interpret the wide variability of inhibitor risk conferred by PTCs in patients.

We believe that quantitative ranking of PTCs without WT Rdthr, based on the affinity of Rdthr-derived missense variants (Supplementary Table S15), deserves to be explored to improve performance of clinical risk prediction models for inhibitor formation in HA.

The presence of PTC class-specific differences (Fig. 5B) permits further classification to be exploited in the presence of HLA genotyping. For UGA, affinity values close to 0 will indicate PTCs with potentially favorable features. Differently, UAA values close to or higher than 0 will identify PTCs with less favorable features. Noticeably, UAA and UGA PTCs displayed differential mean affinity, highly positive for UGA PTCs associated with inhibitors and highly negative for UAA PTCs not associated with inhibitors that resembled the classification of their tetranucleotides into the inverse relation between presence of WT Rdthr and inhibitor occurrence. With respect to this, also a logistic regression, including the stop codon type, domains, 4th nucleotide, and WT readthrough susceptibility (data not shown), highlighted the UAGC as the tetranucleotide potentially associated with inhibitor occurrence. Ranking of PTC tetranucleotides, which may permit to infer a degree of risk in the 10–30% range (Fig. 2B) needs to be explored for individual (*F8* and HLA) genetic features.

Our findings contribute to interpreting previous observations concerning FVIII domain differences in higher inhibitor risk. Whereas the half-reduced incidence of the WT Rdthr in the A3-C1-C2 domains contributes to explain the increased inhibitor risk observed for PTCs located in this FVIII portion, the modest mean differences in affinity between A3-C1-C2 PTCs associated or not with inhibitor may complicate the exploitation of Rdthr-derived missense variants -MHC affinity for inhibitor risk prediction. On the other hand, fully negative or positive values may pinpoint, after HLA genotyping, individual *F8*/*HLA-*genotypes associated with low or high inhibitor risk, respectively, even for the LC. PTCs in the B domain, whose biosynthesis and processing features favor Rdthr output (Ferrarese et al. 2018; Testa et al. 2023) and may lower inhibitor risk (Testa et al. 2023), displayed the lowest negative differential affinity in patients without inhibitors. Overall, the prediction based on *F8* and DRB/HLA genotypes depicts the wide heterogeneity of combinations. These predictions could be implemented by PTC-specific information about nonsense -mediated mRNA decay, expected to modulate endogenous antigen availability (David et al. 2003) and to be partially counteracted by the translational Rdthr (Keeling et al. 2014).

The analysis of WT Rdthr and differential affinity, extended to all PTCs predicted from SNV in the whole *F8* coding region, may also contribute to estimate the inhibitor risk in the numerous, heterogeneously distributed and rare PTCs that could potentially arise in the human population as cause of severe HA.

We are conscious that this work has limitations: i) the inhibitor information may be incomplete in databases, but clinically significant high-titer inhibitors would more likely be present, and our analysis was substantially strengthened by exploring *F8* PTCs found in two or more patients with inhibitors as well as by considering in the group of Pts^inh=0^ only patients for whom the absence of inhibitor was explicitly reported in the database; ii) a small proportion of patients with HA has additional structural variants or deletion/duplication events not captured by standard sequencing (Manderstedt et al. 2026). These genetic conditions would randomly affect both WT Rdthr + and Rdthr- PTCs, impede expression of both FL- WT FVIII and FL missense FVIII variants, and increase the inhibitor risk. However, we expect that detection of these additional gene lesions (3%) would improve the observed PTC Rdthr and inhibitor occurrence association. iii) The evaluation of FVIII peptide affinity for the MHC alleles conducted by mean values does not permit to evaluate the impact of MHC variability in single patients, feasible only after HLA genotyping; iv) The evaluation of FVIII missense peptide affinity for the MHC alleles was conducted by the most represented amino acid residues arising from Rdthr. Amino acids inserted with lower frequency could further modulate the reported inhibitor association. Nevertheless, our study provides a significant relation between inhibitors and a genetically predicted main Rdthr output. v) Although “in silico” prediction may over call the risk of inhibitor occurrence (Hart 2020) for Rdthr-derived missense variants, our analysis newly estimates the WT Rdthr as a potentially protective genetic determinant.

## Conclusions

In conclusion, our exploratory and hypothesis-generating studies provide a new classification of PTCs based on predicted Rdthr output and its affinity for HLA alleles. They support the presence of subtle differences in *F8* nonsense variants expression associated with a replacement treatment-related HA phenotype. Overall, we provide the hemophilia community with novel information for around 1000 PTCs, to be profitably utilized after prospective validation and genotyping for HLA alleles. The genotype-based results of our studies deserve to be explored in other inherited human diseases characterized by protein replacement treatment.

## Supplementary Information

Supplementary Material 1.

Supplementary Material 2.

Supplementary Material 3.

Supplementary Material 4.

Supplementary Material 5.

## Data Availability

All the data generated or analysed during this study are included in this published article and its additional files.

## Abbreviations

PTC: Premature termination codon
Rdthr: Ribosome readthrough
FVIII: Factor VIII
HA: Hemophilia A
WT: Wild-type
eFVIII: Endogenous FVIII
tFVIII: Therapeutic FVIII
CHAMP: CDC Hemophilia A Mutation Project
HC: Heavy chain
LC: Light chain
MHC: Major Histocompatibility Complex
Pts: Patients
PTC^inh=0^: PTCs for which inhibitor was absent or not reported
PTC^inh^^≥^^1^: PTCs for which at least 1 patient with inhibitor was reported
PTC^inh^^≥^^2^: PTCs for which at least 2 patients with inhibitors were reported
PTC^inh^^≥^^3^: PTCs for which at least 3 patients with inhibitors were reported
PCA: Principal Component Analysis
FL: Full-length
