# Supplementary material for: Immunogenic implications of translational readthrough modulate the association of F8 nonsense mutations with inhibitors in Hemophilia A
Source: Mol Med. 2026 Jun 27;32:144. doi: 10.1186/s10020-026-01536-y (PMC13576119; doi:10.1186/s10020-026-01536-y)
Supplement: Supplementary file 1 [file 10020_2026_1536_MOESM1_ESM.pdf]

Supplementary Table S1

Features of *F8* nonsense variants (n=335) with data from 1048 patients reported in EAHAD database. Pts:patients; PTC: Premature Termination Codon; Pts[inh]:patients with inhibitor; WT Rdthr: Wild-Type Readthrough; NR: not reported. *Luciferase assay + indicated recombinantly expressed *F8* PTCs;

A. PTCs in heavy chain (A1-A2); B. PTCs in B domain; C. PTCs in light chain (A3-C1-C2)

A

n Pts	Protein variant	Domain	PTC	4th nucleotide	Inhibitor	Pts[inh]	WT Rdthr	WT amino acid	Predicted missense change	Luciferase assay*
20	p.Arg15Ter	ignal Pepti	TGA	T	NR	1	-	R	W	
1	p.Tyr24Ter	A1	TAG	T	-	0	+	Y	Y/Q	
2	p.Trp33Ter	A1	TAG	G	NR	0	-	W	Y/Q	
1	p.Ser62Ter	A1	TGA	G	NR	0	-	S	W	
1	p.Tyr65Ter	A1	TAA	A	-	0	+	Y	Y	
1	p.Lys67Ter	A1	TAG	A	-	0	-	K	Y/Q	
1	p.Glu72Ter	A1	TAA	T	-	0	-	E	Y	
1	p.Trp87Ter	A1	TGA	A	-	0	+	W	W	
1	p.Gln96Ter	A1	TAG	G	-	0	-	Q	Y/Q	
1	p.Tyr100Ter	A1	TAG	G	+	1	+	Y	Y/Q	
1	p.Lys108Ter	A1	TAG	A	+	1	-	K	Y/Q	
1	p.Tyr124Ter	A1	TAG	T	-	0	+	Y	Y/Q	
1	p.Glu132Ter	A1	TAA	T	-	0	-	E	Y	
2	p.Gln136Ter	A1	TAG	A	NR	1	-	Q	Y/Q	
2	p.Gln139Ter	A1	TAA	A	NR	0	+	Q	Y	
2	p.Glu141Ter	A1	TAG	A	NR	0	-	E	Y/Q	
1	p.Lys146Ter	A1	TAA	G	-	0	-	K	Y	
1	p.Trp157Ter	A1	TAG	C	+	1	-	W	Y/Q	
1	p.Trp157Ter	A1	TGA	C	-	0	+	W	W	
4	p.Gln158Ter	A1	TAG	G	NR	0	-	Q	Y/Q	
2	p.Cys172Ter	A1	TGA	C	NR	0	-	C	W	
1	p.Tyr175Ter	A1	TAA	T	-	0	+	Y	Y	
2	p.Leu187Ter	A1	TAG	A	NR	0	-	L	Y/Q	
1	p.Ser189Ter	A1	TAA	G	-	0	-	S	Y	
1	p.Ser189Ter	A1	TGA	G	-	0	-	S	W	
1	p.Arg199Ter	A1	TGA	G	-	0	-	R	W	
3	p.Gln209Ter	A1	TAG	A	NR	0	-	Q	Y/Q	
1	p.Glu223Ter	A1	TAA	G	-	0	-	E	Y	
1	p.Trp227Ter	A1	TAG	C	-	0	-	W	Y/Q	
2	p.Trp227Ter	A1	TGA	C	NR	0	+	W	W	
2	p.Ser229Ter	A1	TGA	G	NR	0	-	S	W	0,50
2	p.Gln237Ter	A1	TAG	G	-	0	-	Q	Y/Q	
2	p.Trp247Ter	A1	TGA	C	NR	0	+	W	W	1.13
1	p.Tyr256Ter	A1	TAG	G	NR	0	+	Y	Y/Q	
1	p.Gly266Ter	A1	TGA	T	-	0	-	G	W	
5	p.Trp274Ter	A1	TAG	C	NR	2	-	W	Y/Q	
5	p.Trp274Ter	A1	TGA	C	NR	1	+	W	W	
1	p.Glu284Ter	A1	TAA	G	-	0	-	E	Y	
1	p.Ser287Ter	A1	TAA	A	-	0	-	S	Y	
1	p.Glu291Ter	A1	TAA	G	-	0	-	E	Y	
1	p.Gln324Ter	A1	TAG	T	-	0	-	Q	Y/Q	
3	p.Gln335Ter	A1	TAA	C	NR	0	+	Q	Y	
3	p.Tyr342Ter	A1	TAA	G	NR	0	+	Y	Y	
1	p.Lys344Ter	A1	TAA	G	-	0	-	K	Y	
1	p.Glu350Ter	A1	TAG	G	-	0	-	E	Y/Q	
1	p.Gln353Ter	A1	TAA	C	-	0	+	Q	Y	
44	p.Arg355Ter	A1	TGA	A	NR	4	-	R	W	1.68
1	p.Tyr365Ter	a1	TAG	G	NR	0	+	Y	Y/Q	
1	p.Gln389Ter	a1	TAA	A	-	0	+	Q	Y	
1	p.Ser392Ter	a1	TAA	G	-	0	-	S	Y	
1	p.Ser392Ter	a1	TGA	G	-	0	-	S	W	
1	p.Trp401Ter	A2	TAG	G	NR	0	-	W	Y/Q	
2	p.Trp401Ter	A2	TGA	G	NR	1	+	W	W	
2	p.Trp412Ter	A2	TAG	G	NR	0	-	W	Y/Q	
2	p.Trp412Ter	A2	TGA	G	NR	0	+	W	W	
1	p.Leu417Ter	A2	TGA	G	-	0	-	L	W	
1	p.Gln429Ter	A2	TAA	T	NR	0	+	Q	Y	
1	p.Gln436Ter	A2	TAG	C	+	1	-	Q	Y/Q	
33	p.Arg446Ter	A2	TGA	T	NR	9	-	R	W	1.06
1	p.Glu453Ter	A2	TAA	A	-	0	-	E	Y	
1	p.Gln462Ter	A2	TAG	C	+	1	-	Q	Y/Q	
1	p.Glu464Ter	A2	TAA	T	NR	0	-	E	Y	
1	p.Ser465Ter	A2	TGA	G	-	0	-	S	W	
2	p.Tyr473Ter	A2	TAG	G	NR	0	+	Y	Y/Q	
1	p.Tyr473Ter	A2	TAA	G	+	1	+	Y	Y	
1	p.Lys485Ter	A2	TAG	A	-	0	-	K	Y/Q	
1	p.Gln487Ter	A2	TAA	G	-	0	+	Q	Y	
4	p.Tyr495Ter	A2	TAG	C	NR	2	+	Y	Y/Q	
2	p.Gly498Ter	A2	TGA	A	NR	0	-	G	W	
5	p.Ser507Ter	A2	TGA	A	NR	2	-	S	W	1.02
1	p.Arg509Ter	A2	TGA	T	-	0	-	R	W	
1	p.Leu510Ter	A2	TAA	C	NR	0	-	L	Y	
3	p.Trp532Ter	A2	TAG	A	NR	1	-	W	Y/Q	
1	p.Trp532Ter	A2	TGA	A	-	0	+	W	W	
1	p.Ser543Ter	A2	TGA	G	-	0	-	S	W	
1	p.Tyr551Ter	A2	TAG	T	-	0	+	Y	Y/Q	
2	p.Tyr552Ter	A2	TAA	T	NR	1	+	Y	Y	
1	p.Glu559Ter	A2	TAG	A	-	0	-	E	Y/Q	
1	p.Glu576Ter	A2	TAA	T	-	0	-	E	Y	
2	p.Gln580Ter	A2	TAA	A	NR	0	+	Q	Y	
1	p.Ser587Ter	A2	TAA	G	NR	0	-	S	Y	
1	p.Glu600Ter	A2	TAG	A	-	0	-	E	Y/Q	
37	p.Arg602Ter	A2	TGA	A	NR	6	-	R	W	1.16
1	p.Trp604Ter	A2	TAG	T	-	0	-	W	Y/Q	
3	p.Trp604Ter	A2	TGA	T	NR	1	+	W	W	
1	p.Tyr605Ter	A2	TAA	C	-	0	+	Y	Y	
1	p.Tyr605Ter	A2	TAG	C	-	0	+	Y	Y/Q	
5	p.Gln611Ter	A2	TAA	C	NR	2	+	Q	Y	1.05
2	p.Gln621Ter	A2	TAG	C	NR	0	-	Q	Y/Q	
1	p.Gln628Ter	A2	TAA	G	-	0	+	Q	Y	
1	p.Tyr639Ter	A2	TAA	G	-	0	+	Y	Y	
5	p.Gln645Ter	A2	TAG	T	NR	2	-	Q	Y/Q	1.21
4	p.Tyr655Ter	A2	TAG	T	NR	1	+	Y	Y/Q	
4	p.Trp656Ter	A2	TAG	T	NR	0	-	W	Y/Q	
4	p.Trp656Ter	A2	TGA	T	NR	0	+	W	W	
2	p.Gly674Ter	A2	TGA	T	NR	0	-	G	W	
1	p.Lys678Ter	A2	TAA	C	-	0	-	K	Y	
1	p.Ser693Ter	A2	TGA	G	NR	0	-	S	W	
3	p.Ser700Ter	A2	TAG	A	NR	0	-	S	Y/Q	
3	p.Trp707Ter	A2	TAG	A	NR	0	-	W	Y/Q	
1	p.Trp707Ter	A2	TGA	A	-	0	+	W	W	
1	p.Cys711Ter	A2	TGA	C	-	0	-	C	W	
2	p.Tyr737Ter	a2	TAA	T	NR	0	+	Y	Y	
1	p.Tyr738Ter	a2	TAG	G	NR	0	+	Y	Y/Q	
1	p.Glu739Ter	a2	TAG	G	-	0	-	E	Y/Q	
1	p.Tyr748Ter	a2	TAA	T	NR	0	+	Y	Y	
1	p.Leu749Ter	a2	TAG	C	-	0	-	L	Y/Q	

B

n Pts	Protein variant	Domain	PTC	4th nucleotide	Inhibitor	Pts[inh]	WT Rdthr	WT amino acid	Predicted missense change	Luciferase assay*
3	p.Gln772Ter	B	TAA	A	NR	0	+	Q	Y	1,58
3	p.Trp791Ter	B	TAG	T	-	0	-	W	Y/Q	
4	p.Trp791Ter	B	TGA	T	NR	0	+	W	W	2,05
1	p.Gln802Ter	B	TAA	A	-	0	+	Q	Y	
51	p.Arg814Ter	B	TGA	C	NR	4	-	R	W	1,78
4	p.Gln815Ter	B	TAG	A	NR	1	-	Q	Y/Q	2,22
1	p.Gln828Ter	B	TAA	G	-	0	+	Q	Y	
2	p.Glu829Ter	B	TAA	G	NR	0	-	E	Y	
2	p.Gln859Ter	B	TAG	C	NR	1	-	Q	Y/Q	
4	p.Ser872Ter	B	TGA	G	NR	0	-	S	W	2,74
1	p.Ser872Ter	B	TAA	G	-	0	-	S	Y	
1	p.Gln875Ter	B	TAA	T	+	1	+	Q	Y	
2	p.Leu878Ter	B	TGA	A	-	1	-	L	W	4,36
1	p.Glu880Ter	B	TAG	A	NR	0	-	E	Y/Q	
2	p.Ser901Ter	B	TAA	A	NR	0	-	S	Y	
1	p.Ser901Ter	B	TGA	A	+	1	-	S	W	
1	p.Leu923Ter	B	TGA	G	+	1	-	L	W	
1	p.Gly924Ter	B	TGA	C	NR	0	-	G	W	
1	p.Gly953Ter	B	TGA	C	+	1	-	G	W	
1	p.Lys965Ter	B	TAG	T	NR	0	-	K	Y/Q	
1	p.Glu968Ter	B	TAA	T	-	0	-	E	Y	
1	p.Leu971Ter	B	TGA	A	-	0	-	L	W	
1	p.Ser978Ter	B	TAA	T	-	0	-	S	Y	
5	p.Trp979Ter	B	TAG	G	NR	0	-	W	Y/Q	2,82
3	p.Trp979Ter	B	TGA	G	NR	0	+	W	W	2,5
1	p.Ser985Ter	B	TAA	A	-	0	-	S	Y	
1	p.Lys993Ter	B	TAA	G	NR	0	-	K	Y	
1	p.Lys1011Ter	B	TAA	G	-	0	-	K	Y	
1	p.Ser1038Ter	B	TGA	T	+	1	-	S	W	
1	p.Ser1038Ter	B	TAA	T	+	1	-	S	Y	
2	p.Trp1048Ter	B	TAG	C	NR	0	-	W	Y/Q	2,54
2	p.Trp1048Ter	B	TGA	C	NR	0	+	W	W	
1	p.Leu1052Ter	B	TAA	G	NR	0	-	L	Y	
1	p.Glu1053Ter	B	TAA	A	-	0	-	E	Y	
10	p.Lys1059Ter	B	TAA	A	NR	2	-	K	Y	1,59
1	p.Lys1060Ter	B	TAA	G	-	0	-	K	Y	
1	p.Ser1084Ter	B	TGA	A	-	0	-	S	W	
1	p.Ser1090Ter	B	TGA	A	-	0	-	S	W	
1	p.Gln1097Ter	B	TAA	C	NR	0	+	Q	Y	
4	p.Gln1098Ter	B	TAG	A	NR	0	-	Q	Y/Q	2,57
1	p.Lys1100Ter	B	TAA	G	+	1	-	K	Y	
4	p.Gln1109Ter	B	TAA	A	NR	0	+	Q	Y	3,01
1	p.Ser1124Ter	B	TAA	G	+	1	-	S	Y	
5	p.Trp1127Ter	B	TGA	A	NR	1	+	W	W	2,44
1	p.Gln1129Ter	B	TAA	A	-	0	+	Q	Y	
2	p.Gln1141Ter	B	TAA	G	NR	0	+	Q	Y	
2	p.Gln1160Ter	B	TAG	A	NR	0	-	Q	Y/Q	
1	p.Glu1165Ter	B	TAG	A	-	0	-	E	Y/Q	2,16
1	p.Lys1166Ter	B	TAA	A	+	1	-	K	Y	
2	p.Glu1203Ter	B	TAA	A	NR	0	-	E	Y	
1	p.Lys1211Ter	B	TAA	A	NR	0	-	K	Y	
1	p.Lys1212Ter	B	TAA	A	-	0	-	K	Y	
2	p.Gln1214Ter	B	TAG	G	NR	0	-	Q	Y/Q	
3	p.Glu1216Ter	B	TAA	A	NR	0	-	E	Y	
1	p.Leu1223Ter	B	TAA	A	-	0	-	L	Y	
2	p.Gln1225Ter	B	TAA	G	NR	0	+	Q	Y	
3	p.Gln1232Ter	B	TAG	A	NR	0	-	Q	Y/Q	6,12
1	p.Glu1256Ter	B	TAA	G	NR	0	-	E	Y	
3	p.Gln1268Ter	B	TAA	G	NR	0	+	Q	Y	
1	p.Ser1276Ter	B	TGA	A	NR	0	-	S	W	
1	p.Ser1276Ter	B	TAA	A	-	0	-	S	Y	
1	p.Lys1282Ter	B	TAA	C	-	0	-	K	Y	
1	p.Lys1289Ter	B	TAA	A	NR	0	-	K	Y	2,06
1	p.Lys1290Ter	B	TAA	G	NR	0	-	K	Y	
1	p.Gln1302Ter	B	TAA	A	+	1	+	Q	Y	
3	p.Gln1305Ter	B	TAA	A	NR	0	+	Q	Y	
1	p.Glu1308Ter	B	TAG	A	-	0	-	E	Y/Q	
1	p.Gln1322Ter	B	TAG	C	-	0	-	Q	Y/Q	
2	p.Gln1323Ter	B	TAG	A	-	1	-	Q	Y/Q	
2	p.Gln1328Ter	B	TAA	C	NR	0	+	Q	Y	
1	p.Arg1332Ter	B	TGA	G	NR	0	-	R	W	
4	p.Gln1336Ter	B	TAA	T	NR	0	+	Q	Y	4,13
1	p.Glu1343Ter	B	TAA	A	-	0	-	E	Y	
2	p.Glu1347Ter	B	TAA	A	NR	0	-	E	Y	
2	p.Lys1348Ter	B	TAA	A	NR	0	-	K	Y	
1	p.Ser1356Ter	B	TAA	A	-	0	-	S	Y	
3	p.Gln1358Ter	B	TAG	T	NR	0	-	Q	Y/Q	
3	p.Trp1359Ter	B	TAG	T	NR	1	-	W	Y/Q	
3	p.Trp1359Ter	B	TGA	T	NR	0	+	W	W	
1	p.Tyr1376Ter	B	TAA	A	-	0	+	Y	Y	
4	p.Gln1386Ter	B	TAG	T	NR	1	-	Q	Y/Q	5,65
1	p.Ser1390Ter	B	TGA	G	-	0	-	S	W	
1	p.Gln1401Ter	B	TAA	G	-	0	+	Q	Y	
1	p.Arg1404Ter	B	TGA	T	+	1	-	R	W	
1	p.Ser1414Ter	B	TAA	T	-	0	-	S	Y	
1	p.Glu1449Ter	B	TAA	A	-	0	-	E	Y	
3	p.Gln1455Ter	B	TAA	G	NR	0	+	Q	Y	
1	p.Glu1470Ter	B	TAG	A	-	0	-	E	Y/Q	
2	p.Gln1475Ter	B	TAA	A	NR	0	+	Q	Y	
1	p.Tyr1491Ter	B	TAG	A	-	0	+	Y	Y/Q	
2	p.Tyr1491Ter	B	TAA	A	NR	1	+	Y	Y	
1	p.Lys1492Ter	B	TAG	A	+	1	-	K	Y/Q	
1	p.Glu1495Ter	B	TAG	A	+	1	-	E	Y/Q	
1	p.Lys1506Ter	B	TAA	A	NR	0	-	K	Y	
1	p.Glu1512Ter	B	TAA	T	-	0	-	E	Y	
1	p.Gln1521Ter	B	TAG	A	-	0	-	Q	Y/Q	
1	p.Lys1522Ter	B	TAG	G	-	0	-	K	Y/Q	
1	p.Trp1554Ter	B	TAG	A	NR	0	-	W	Y/Q	
4	p.Trp1554Ter	B	TGA	A	NR	2	+	W	W	
1	p.Lys1562Ter	B	TAA	G	NR	0	-	K	Y	
3	p.Trp1586Ter	B	TAG	G	NR	1	-	W	Y/Q	
2	p.Trp1586Ter	B	TGA	G	NR	1	+	W	W	
1	p.Tyr1590Ter	B	TAA	G	-	0	+	Y	Y	
4	p.Glu1597Ter	B	TAA	G	NR	0	-	E	Y	2,35
2	p.Trp1599Ter	B	TAG	A	NR	1	-	W	Y/Q	
1	p.Trp1599Ter	B	TGA	A	-	0	+	W	W	
1	p.Lys1600Ter	B	TAA	T	-	0	-	K	Y	
1	p.Glu1603Ter	B	TAG	A	-	0	-	E	Y/Q	
1	p.Ser1605Ter	B	TAA	C	NR	0	-	S	Y	
1	p.Glu1607Ter	B	TAA	A	-	0	-	E	Y	
2	p.Lys1614Ter	B	TAG	G	NR	0	-	K	Y/Q	
3	p.Glu1634Ter	B	TAG	G	NR	0	-	E	Y/Q	2,2
2	p.Gln1636Ter	B	TAA	A	NR	0	+	Q	Y	
1	p.Glu1640Ter	B	TAA	A	-	0	-	E	Y	
1	p.Trp1645Ter	B	TAG	G	+	1	-	W	Y/Q	
3	p.Trp1645Ter	B	TGA	G	NR	1	+	W	W	3,09
1	p.Gln1648Ter	B	TAA	G	NR	0	+	Q	Y	
1	p.Gln1657Ter	B	TAA	A	-	0	+	Q	Y	
1	p.Lys1663Ter	B	TAA	C	-	0	-	K	Y	
1	p.Gln1666Ter	B	TAA	C	-	0	+	Q	Y	

C

n Pts	Protein variant	Domain	PTC	4th nucleotide	Inhibitor	Pts[inh]	WT Rdthr	WT amino acid	Predicted missense change	Luciferase assay*
1	p.Gln1675Ter	a3	TAG	T	-	0	-	Q	Y/Q	
1	p.Glu1680Ter	a3	TAA	A	+	1	-	E	Y	
1	p.Ser1688Ter	a3	TGA	G	NR	0	-	S	W	
1	p.Glu1694Ter	a3	TAA	G	+	1	-	E	Y	
2	p.Glu1701Ter	a3	TAG	G	-	0	-	E	Y/Q	
7	p.Gln1705Ter	a3	TAG	A	NR	2	-	Q	Y/Q	0,6
23	p.Arg1715Ter	A3	TGA	C	NR	7	-	R	W	2,1
1	p.Tyr1717Ter	A3	TAG	T	-	0	+	Y	Y/Q	
1	p.Glu1723Ter	A3	TAG	A	-	0	-	E	Y/Q	
4	p.Trp1726Ter	A3	TAG	G	NR	2	-	W	Y/Q	0,4
2	p.Trp1726Ter	A3	TGA	G	NR	0	+	W	W	1,58
1	p.Tyr1728Ter	A3	TAA	G	-	0	+	Y	Y	
1	p.Gln1748Ter	A3	TAG	T	NR	0	-	Q	Y/Q	
1	p.Lys1751Ter	A3	TAA	G	NR	0	-	K	Y	
1	p.Gln1755Ter	A3	TAG	G	-	0	-	Q	Y/Q	
3	p.Tyr1767Ter	A3	TAA	C	NR	0	+	Y	Y	
3	p.Tyr1767Ter	A3	TAG	C	NR	1	+	Y	Y/Q	
2	p.Glu1773Ter	A3	TAA	C	NR	0	-	E	Y	
1	p.Tyr1781Ter	A3	TAA	A	NR	0	+	Y	Y	
3	p.Gln1797Ter	A3	TAG	G	NR	0	-	Q	Y/Q	
1	p.Tyr1802Ter	A3	TAG	T	NR	0	+	Y	Y/Q	
1	p.Tyr1805Ter	A3	TAA	T	-	0	+	Y	Y	
1	p.Tyr1811Ter	A3	TAA	G	+	1	+	Y	Y	
1	p.Glu1812Ter	A3	TAG	G	+	1	-	E	Y/Q	
2	p.Gln1815Ter	A3	TAG	A	NR	1	-	Q	Y/Q	
2	p.Gly1818Ter	A3	TGA	G	NR	0	-	G	W	
2	p.Trp1836Ter	A3	TAG	A	NR	0	-	W	Y/Q	
2	p.Trp1836Ter	A3	TGA	A	-	0	+	W	W	3,38
1	p.Lys1837Ter	A3	TAA	G	+	1	-	K	Y	
3	p.Gln1839Ter	A3	TAA	C	NR	1	+	Q	Y	
[illegible]	[illegible]	[illegible]	[illegible]	[illegible]	[illegible]	[illegible]	[illegible]	[illegible]	[illegible]	[illegible]
