# Supplementary material for: Immunogenic implications of translational readthrough modulate the association of F8 nonsense mutations with inhibitors in Hemophilia A
Source: Mol Med. 2026 Jun 27;32:144. doi: 10.1186/s10020-026-01536-y (PMC13576119; doi:10.1186/s10020-026-01536-y)
Supplement: Supplementary file 2 [file 10020_2026_1536_MOESM2_ESM.pdf]

Change_HGVS	Protein variant	WT triplet	Stop Triplet	Domain	HC-B-LC	Observed/Predicted
c.4C>T	p.Q2X	CAA	TAA	Pre-peptide	Pre-peptide	Predicted
c.72C>A	p.Y24X	TAC	TAA	A1	HC	Predicted
c.72C>G	p.Y24X	TAC	TAG	A1	HC	**Observed**
c.75C>A	p.Y25X	TAC	TAA	A1	HC	Predicted
c.75C>G	p.Y25X	TAC	TAG	A1	HC	Predicted
c.99G>A	p.W33X	TGG	TGA	A1	HC	Predicted
c.105T>A	p.Y35X	TAT	TAA	A1	HC	Predicted
c.105T>G	p.Y35X	TAT	TAG	A1	HC	Predicted
c.109C>T	p.Q37X	CAA	TAA	A1	HC	Predicted
c.195C>A	p.Y65X	TAC	TAA	A1	HC	**Observed**
c.195C>G	p.Y65X	TAC	TAG	A1	HC	Predicted
c.261G>A	p.W87X	TGG	TGA	A1	HC	**Observed**
c.300T>A	p.Y100X	TAT	TAA	A1	HC	Predicted
c.300T>G	p.Y100X	TAT	TAG	A1	HC	**Observed**
c.372C>A	p.Y124X	TAC	TAA	A1	HC	Predicted
c.372C>G	p.Y124X	TAC	TAG	A1	HC	**Observed**
c.375G>A	p.W125X	TGG	TGA	A1	HC	Predicted
c.399T>A	p.Y133X	TAT	TAA	A1	HC	Predicted
c.399T>G	p.Y133X	TAT	TAG	A1	HC	Predicted
c.415C>T	p.Q139X	CAA	TAA	A1	HC	**Observed**
c.465T>A	p.Y155X	TAT	TAA	A1	HC	Predicted
c.465T>G	p.Y155X	TAT	TAG	A1	HC	Predicted
c.471G>A	p.W157X	TGG	TGA	A1	HC	**Observed**
c.525C>A	p.Y175X	TAC	TAA	A1	HC	**Observed**
c.525C>G	p.Y175X	TAC	TAG	A1	HC	Predicted
c.531T>A	p.Y177X	TAT	TAA	A1	HC	Predicted
c.531T>G	p.Y177X	TAT	TAG	A1	HC	Predicted
c.681G>A	p.W227X	TGG	TGA	A1	HC	**Observed**
c.741G>A	p.W247X	TGG	TGA	A1	HC	**Observed**
c.768T>A	p.Y256X	TAT	TAA	A1	HC	Predicted
c.768T>G	p.Y256X	TAT	TAG	A1	HC	**Observed**
c.819T>A	p.Y273X	TAT	TAA	A1	HC	Predicted
c.819T>G	p.Y273X	TAT	TAG	A1	HC	Predicted
c.822G>A	p.W274X	TGG	TGA	A1	HC	**Observed**
c.946C>T	p.Q316X	CAA	TAA	A1	HC	Predicted
c.1003C>T	p.Q335X	CAA	TAA	A1	HC	**Observed**
c.1026T>A	p.Y342X	TAT	TAA	A1	HC	**Observed**
c.1026T>G	p.Y342X	TAT	TAG	A1	HC	Predicted
c.1057C>T	p.Q353X	CAA	TAA	A1	HC	**Observed**
c.1095T>G	p.Y365X	TAT	TAG	a1	HC	**Observed**
c.1095T>A	p.Y365X	TAT	TAA	a1	HC	Predicted
c.1165C>T	p.Q389X	CAA	TAA	a1	HC	**Observed**
c.1203G>A	p.W401X	TGG	TGA	A2	HC	**Observed**
c.1212C>A	p.Y404X	TAC	TAA	A2	HC	Predicted
c.1212C>G	p.Y404X	TAC	TAG	A2	HC	Predicted
c.1236G>A	p.W412X	TGG	TGA	A2	HC	**Observed**
c.1242T>A	p.Y414X	TAT	TAA	A2	HC	Predicted
c.1242T>G	p.Y414X	TAT	TAG	A2	HC	Predicted
c.1278T>A	p.Y426X	TAT	TAA	A2	HC	Predicted
c.1278T>G	p.Y426X	TAT	TAG	A2	HC	Predicted
c.1285C>T	p.Q429X	CAA	TAA	A2	HC	**Observed**
c.1290T>A	p.Y430X	TAT	TAA	A2	HC	Predicted
c.1290T>G	p.Y430X	TAT	TAG	A2	HC	Predicted
c.1326C>G	p.Y442X	TAC	TAG	A2	HC	Predicted
c.1326C>A	p.Y442X	TAC	TAA	A2	HC	Predicted
c.1350C>A	p.Y450X	TAC	TAA	A2	HC	Predicted
c.1350C>G	p.Y450X	TAC	TAG	A2	HC	Predicted
c.1419T>A	p.Y473X	TAT	TAA	A2	HC	**Observed**
c.1419T>G	p.Y473X	TAT	TAG	A2	HC	**Observed**
c.1459C>T	p.Q487X	CAA	TAA	A2	HC	**Observed**
c.1476T>A	p.Y492X	TAT	TAA	A2	HC	Predicted
c.1476T>G	p.Y492X	TAT	TAG	A2	HC	Predicted
c.1485C>A	p.Y495X	TAC	TAA	A2	HC	Predicted
c.1485C>G	p.Y495X	TAC	TAG	A2	HC	**Observed**
c.1518T>A	p.Y506X	TAT	TAA	A2	HC	Predicted
c.1518T>G	p.Y506X	TAT	TAG	A2	HC	Predicted
c.1590T>G	p.Y530X	TAT	TAG	A2	HC	Predicted
c.1590T>A	p.Y530X	TAT	TAA	A2	HC	Predicted
c.1596G>A	p.W532X	TGG	TGA	A2	HC	**Observed**
c.1653T>A	p.Y551X	TAT	TAA	A2	HC	Predicted
c.1653T>G	p.Y551X	TAT	TAG	A2	HC	**Observed**
c.1656C>A	p.Y552X	TAC	TAA	A2	HC	**Observed**
c.1656C>G	p.Y552X	TAC	TAG	A2	HC	Predicted
c.1722C>A	p.Y574X	TAC	TAA	A2	HC	Predicted
c.1722C>G	p.Y574X	TAC	TAG	A2	HC	Predicted
c.1739C>T	p.Q580X	CAA	TAA	A2	HC	**Observed**
c.1812G>A	p.W604X	TGG	TGA	A2	HC	**Observed**
c.1815C>A	p.Y605X	TAC	TAA	A2	HC	**Observed**
c.1815C>G	p.Y605X	TAC	TAG	A2	HC	**Observed**
c.1831C>T	p.Q611X	CAA	TAA	A2	HC	**Observed**
c.1882C>T	p.Q628X	CAA	TAA	A2	HC	**Observed**
c.1917T>A	p.Y639X	TAT	TAA	A2	HC	**Observed**
c.1917T>G	p.Y639X	TAT	TAG	A2	HC	Predicted
c.1965C>A	p.Y655X	TAC	TAA	A2	HC	Predicted
c.1965C>G	p.Y655X	TAC	TAG	A2	HC	**Observed**
c.1968G>A	p.W656X	TGG	TGA	A2	HC	**Observed**
c.1971C>A	p.Y657X	TAC	TAA	A2	HC	Predicted
c.1971C>G	p.Y657X	TAC	TAG	A2	HC	Predicted
c.2025T>G	p.Y675X	TAT	TAG	A2	HC	Predicted
c.2025T>A	p.Y675X	TAT	TAA	A2	HC	Predicted
c.2049T>A	p.Y683X	TAT	TAA	A2	HC	Predicted
c.2049T>G	p.Y683X	TAT	TAG	A2	HC	Predicted
c.2121G>A	p.W707X	TGG	TGA	A2	HC	**Observed**
c.2211T>A	p.Y737X	TAT	TAA	a2	HC	**Observed**
c.2211T>G	p.Y737X	TAT	TAG	a2	HC	Predicted
c.2214C>A	p.Y738X	TAC	TAA	a2	HC	Predicted
c.2214C>G	p.Y738X	TAC	TAG	a2	HC	**Observed**
c.2226T>A	p.Y742X	TAT	TAA	a2	HC	Predicted
c.2226T>G	p.Y742X	TAT	TAG	a2	HC	Predicted
c.2244C>A	p.Y748X	TAC	TAA	a2	HC	**Observed**
c.2244C>G	p.Y748X	TAC	TAG	a2	HC	Predicted
c.2314C>T	p.Q772X	CAA	TAA	B	B	Predicted
c.2320C>T	p.Q774X	CAA	TAA	B	B	**Observed**
c.2373G>A	p.W791X	TGG	TGA	B	B	**Observed**
c.2404C>T	p.Q802X	CAA	TAA	B	B	**Observed**
c.2482C>T	p.Q828X	CAA	TAA	B	B	**Observed**
c.2496T>A	p.Y832X	TAT	TAA	B	B	Predicted
c.2496T>G	p.Y832X	TAT	TAG	B	B	Predicted
c.2623C>T	p.Q875X	CAA	TAA	B	B	**Observed**
c.2796T>A	p.Y932X	TAT	TAA	B	B	Predicted
c.2796T>G	p.Y932X	TAT	TAG	B	B	Predicted
c.2803C>T	p.Q935X	CAA	TAA	B	B	Predicted
c.2923C>T	p.Q975X	CAA	TAA	B	B	Predicted
c.2937G>A	p.W979X	TGG	TGA	B	B	**Observed**
c.3144G>A	p.W1048X	TGG	TGA	B	B	**Observed**
c.3145C>T	p.Q1049X	CAA	TAA	B	B	Predicted
c.3289C>T	p.Q1097X	CAA	TAA	B	B	**Observed**
c.3325C>T	p.Q1109X	CAA	TAA	B	B	**Observed**
c.3381G>A	p.W1127X	TGG	TGA	B	B	**Observed**
c.3385C>T	p.Q1129X	CAA	TAA	B	B	**Observed**
c.3421C>T	p.Q1141X	CAA	TAA	B	B	**Observed**
c.3439C>T	p.Q1147X	CAA	TAA	B	B	Predicted
c.3625C>T	p.Q1209X	CAA	TAA	B	B	Predicted
c.3673C>T	p.Q1225X	CAA	TAA	B	B	**Observed**
c.3757C>T	p.Q1253X	CAA	TAA	B	B	Predicted
c.3777T>A	p.Y1259X	TAT	TAA	B	B	Predicted
c.3777T>G	p.Y1259X	TAT	TAG	B	B	Predicted
c.3789T>A	p.Y1263X	TAT	TAA	B	B	Predicted
c.3789T>G	p.Y1263X	TAT	TAG	B	B	Predicted
c.3802C>T	p.Q1268X	CAA	TAA	B	B	**Observed**
c.3904C>T	p.Q1302X	CAA	TAA	B	B	**Observed**
c.3913C>T	p.Q1305X	CAA	TAA	B	B	**Observed**
c.3930T>A	p.Y1310X	TAT	TAA	B	B	Predicted
c.3930T>G	p.Y1310X	TAT	TAG	B	B	Predicted
c.3982C>T	p.Q1328X	CAA	TAA	B	B	**Observed**
c.4006C>T	p.Q1336X	CAA	TAA	B	B	**Observed**
c.4077G>A	p.W1359X	TGG	TGA	B	B	**Observed**
c.4128C>A	p.Y1376X	TAC	TAA	B	B	**Observed**
c.4128C>G	p.Y1376X	TAC	TAG	B	B	Predicted
c.4201C>T	p.Q1401X	CAA	TAA	B	B	**Observed**
c.4266T>A	p.Y1422X	TAT	TAA	B	B	Predicted
c.4266T>G	p.Y1422X	TAT	TAG	B	B	Predicted
c.4285C>T	p.Q1429X	CAA	TAA	B	B	Predicted
c.4320T>A	p.Y1440X	TAT	TAA	B	B	Predicted
c.4320T>G	p.Y1440X	TAT	TAG	B	B	Predicted
c.4342C>T	p.Q1448X	CAA	TAA	B	B	Predicted
c.4363C>T	p.Q1455X	CAA	TAA	B	B	**Observed**
c.4423C>T	p.Q1475X	CAA	TAA	B	B	**Observed**
c.4473C>A	p.Y1491X	TAC	TAA	B	B	**Observed**
c.4473C>G	p.Y1491X	TAC	TAG	B	B	**Observed**
c.4560T>G	p.Y1520X	TAT	TAG	B	B	Predicted
c.4560T>A	p.Y1520X	TAT	TAA	B	B	Predicted
c.4662G>A	p.W1554X	TGG	TGA	B	B	**Observed**
c.4758G>A	p.W1586X	TGG	TGA	B	B	**Observed**
c.4770T>A	p.Y1590X	TAT	TAA	B	B	**Observed**
c.4770T>G	p.Y1590X	TAT	TAG	B	B	Predicted
c.4797G>A	p.W1599X	TGG	TGA	B	B	**Observed**
c.4804C>T	p.Q1602X	CAA	TAA	B	B	Predicted
c.4906C>T	p.Q1636X	CAA	TAA	B	B	**Observed**
c.4935G>A	p.W1645X	TGG	TGA	B	B	**Observed**
c.4942C>T	p.Q1648X	CAA	TAA	B	B	**Observed**
c.4969C>T	p.Q1657X	CAA	TAA	B	B	**Observed**
c.4996C>T	p.Q1666X	CAA	TAA	B	B	**Observed**
c.5032C>T	p.Q1678X	CAA	TAA	a3	LC	Predicted
c.5049T>A	p.Y1683X	TAT	TAA	a3	LC	Predicted
c.5049T>G	p.Y1683X	TAT	TAG	a3	LC	Predicted
c.5097T>G	p.Y1699X	TAT	TAG	a3	LC	Predicted
c.5097T>A	p.Y1699X	TAT	TAA	a3	LC	Predicted
c.5131C>T	p.Q1711X	CAA	TAA	A3	LC	Predicted
c.5151T>A	p.Y1717X	TAT	TAA	A3	LC	Predicted
c.5151T>G	p.Y1717X	TAT	TAG	A3	LC	**Observed**
c.5178G>A	p.W1726X	TGG	TGA	A3	LC	**Observed**
c.5184T>G	p.Y1728X	TAT	TAG	A3	LC	Predicted
c.5184T>A	p.Y1728X	TAT	TAA	A3	LC	**Observed**
c.5301C>A	p.Y1767X	TAC	TAA	A3	LC	**Observed**
c.5301C>G	p.Y1767X	TAC	TAG	A3	LC	**Observed**
c.5343T>A	p.Y1781X	TAT	TAA	A3	LC	**Observed**
c.5343T>G	p.Y1781X	TAT	TAG	A3	LC	Predicted
c.5406T>A	p.Y1802X	TAT	TAA	A3	LC	Predicted
c.5406T>G	p.Y1802X	TAT	TAG	A3	LC	**Observed**
c.5415T>A	p.Y1805X	TAT	TAA	A3	LC	**Observed**
c.5415T>G	p.Y1805X	TAT	TAG	A3	LC	Predicted
c.5433T>A	p.Y1811X	TAT	TAA	A3	LC	**Observed**
c.5433T>G	p.Y1811X	TAT	TAG	A3	LC	Predicted
c.5449C>T	p.Q1817X	CAA	TAA	A3	LC	Predicted
c.5502C>A	p.Y1834X	TAC	TAA	A3	LC	Predicted
c.5502C>G	p.Y1834X	TAC	TAG	A3	LC	Predicted
c.5508G>A	p.W1836X	TGG	TGA	A3	LC	**Observed**
c.5515C>T	p.Q1839X	CAA	TAA	A3	LC	**Observed**
c.5562G>A	p.W1854X	TGG	TGA	A3	LC	**Observed**
c.5568T>A	p.Y1856X	TAT	TAA	A3	LC	Predicted
c.5568T>G	p.Y1856X	TAT	TAG	A3	LC	**Observed**
c.5665C>T	p.Q1889X	CAA	TAA	A3	LC	**Observed**
c.5724G>A	p.W1908X	TGG	TGA	A3	LC	**Observed**
c.5727C>A	p.Y1909X	TAC	TAA	A3	LC	Predicted
c.5727C>G	p.Y1909X	TAC	TAG	A3	LC	Predicted
c.5805T>A	p.Y1935X	TAT	TAA	A3	LC	**Observed**
c.5805T>G	p.Y1935X	TAT	TAG	A3	LC	Predicted
c.5829C>A	p.Y1943X	TAC	TAA	A3	LC	Predicted
c.5829C>G	p.Y1943X	TAC	TAG	A3	LC	Predicted
c.5869C>T	p.Q1957X	CAA	TAA	A3	LC	**Observed**
c.5883G>A	p.W1961X	TGG	TGA	A3	LC	**Observed**
c.5886T>A	p.Y1962X	TAT	TAA	A3	LC	**Observed**
c.5886T>G	p.Y1962X	TAT	TAG	A3	LC	Predicted
c.5970T>A	p.Y1990X	TAT	TAA	A3	LC	Predicted
c.5970T>G	p.Y1990X	TAT	TAG	A3	LC	Predicted
c.5985C>A	p.Y1995X	TAC	TAA	A3	LC	**Observed**
c.5985C>G	p.Y1995X	TAC	TAG	A3	LC	Predicted
c.5994T>A	p.Y1998X	TAT	TAA	A3	LC	**Observed**
c.5994T>G	p.Y1998X	TAT	TAG	A3	LC	Predicted
c.6045G>A	p.W2015X	TGG	TGA	A3	LC	Predicted
c.6108C>A	p.Y2036X	TAC	TAA	A3	LC	Predicted
c.6108C>G	p.Y2036X	TAC	TAG	A3	LC	Predicted
c.6181C>T	p.Q2061X	CAA	TAA	C1	LC	Predicted
c.6186T>A	p.Y2062X	TAT	TAA	C1	LC	Predicted
c.6186T>G	p.Y2062X	TAT	TAG	C1	LC	Predicted
c.6195G>A	p.W2065X	TGG	TGA	C1	LC	Predicted
c.6222T>A	p.Y2074X	TAT	TAA	C1	LC	Predicted
c.6222T>G	p.Y2074X	TAT	TAG	C1	LC	Predicted
c.6243G>A	p.W2081X	TGG	TGA	C1	LC	**Observed**
c.6267G>A	p.W2089X	TGG	TGA	C1	LC	**Observed**
c.6348C>G	p.Y2116X	TAC	TAG	C1	LC	Predicted
c.6348C>A	p.Y2116X	TAC	TAA	C1	LC	Predicted
c.6372T>A	p.Y2124X	TAT	TAA	C1	LC	Predicted
c.6372T>G	p.Y2124X	TAT	TAG	C1	LC	Predicted
c.6393G>A	p.W2131X	TGG	TGA	C1	LC	**Observed**
c.6402T>A	p.Y2134X	TAT	TAA	C1	LC	Predicted
c.6402T>G	p.Y2134X	TAT	TAG	C1	LC	Predicted
c.6501C>A	p.Y2167X	TAC	TAA	C1	LC	Predicted
c.6501C>G	p.Y2167X	TAC	TAG	C1	LC	Predicted
c.6525T>A	p.Y2175X	TAT	TAA	C1	LC	Predicted
c.6525T>G	p.Y2175X	TAT	TAG	C1	LC	Predicted
c.6642C>A	p.Y2214X	TAC	TAA	C2	LC	Predicted
c.6642C>G	p.Y2214X	TAC	TAG	C2	LC	Predicted
c.6666G>A	p.W2222X	TGG	TGA	C2	LC	Predicted
c.6694C>T	p.Q2232X	CAA	TAA	C2	LC	**Observed**
c.6714G>A	p.W2238X	TGG	TGA	C2	LC	Predicted
c.6744G>A	p.W2248X	TGG	TGA	C2	LC	**Observed**
c.6748C>T	p.Q2250X	CAA	TAA	C2	LC	**Observed**
c.6825T>A	p.Y2275X	TAT	TAA	C2	LC	**Observed**
c.6825T>G	p.Y2275X	TAT	TAG	C2	LC	Predicted
c.6853C>T	p.Q2285X	CAA	TAA	C2	LC	Predicted
c.6870G>A	p.W2290X	TGG	TGA	C2	LC	**Observed**
c.6916C>T	p.Q2306X	CAA	TAA	C2	LC	Predicted
c.6972C>A	p.Y2324X	TAC	TAA	C2	LC	Predicted
c.6972C>G	p.Y2324X	TAC	TAG	C2	LC	**Observed**
c.6996G>A	p.W2332X	TGG	TGA	C2	LC	**Observed**
c.7053C>A	p.Y2351X	TAC	TAA	C2	LC	Predicted
c.7053C>G	p.Y2351X	TAC	TAG	C2	LC	Predicted
