# Supplementary material for: Immunogenic implications of translational readthrough modulate the association of F8 nonsense mutations with inhibitors in Hemophilia A
Source: Mol Med. 2026 Jun 27;32:144. doi: 10.1186/s10020-026-01536-y (PMC13576119; doi:10.1186/s10020-026-01536-y)
Supplement: Supplementary file 3 [file 10020_2026_1536_MOESM3_ESM.pdf]

[illegible]										
c.1063C>T	p.R355X	CGA	TGA	A1	HC	**Observed**	Trp		p.R355W	
c.1069A>T	p.K357X	AAA	TAA	a1	HC	Predicted	Tyr	Gln	p.K357Y	p.K357Q
c.1078G>T	p.E360X	GAA	TAA	a1	HC	Predicted	Tyr	Gln	p.E360Y	p.E360Q
c.1081G>T	p.E361X	GAA	TAA	a1	HC	Predicted	Tyr	Gln	p.E361Y	p.E361Q
c.1087G>T	p.E363X	GAA	TAA	a1	HC	Predicted	Tyr	Gln	p.E363Y	p.E363Q
c.1117G>T	p.E373X	GAA	TAA	a1	HC	Predicted	Tyr	Gln	p.E373Y	p.E373Q
c.1175C>A	p.S392X	TCA	TAA	a1	HC	**Observed**	Tyr	Gln	p.S392Y	p.S392Q
c.1175C>G	p.S392X	TCA	TGA	a1	HC	**Observed**	Trp		p.S392W	
c.1183A>T	p.K395X	AAG	TAG	A2	HC	Predicted	Tyr		p.K395Y	
c.1186A>T	p.K396X	AAG	TAG	A2	HC	Predicted	Tyr		p.K396Y	
c.1195A>T	p.K399X	AAA	TAA	A2	HC	Predicted	Tyr	Gln	p.K399Y	p.K399Q
c.1202G>A	p.W401X	TGG	TAG	A2	HC	**Observed**	Tyr		p.W401Y	
c.1222G>T	p.E408X	GAA	TAA	A2	HC	Predicted	Tyr	Gln	p.E408Y	p.E408Q
c.1225G>T	p.E409X	GAG	TAG	A2	HC	Predicted	Tyr		p.E409Y	
c.1228G>T	p.E410X	GAG	TAG	A2	HC	Predicted	Tyr		p.E410Y	
c.1235G>A	p.W412X	TGG	TAG	A2	HC	**Observed**	Tyr		p.W412Y	
c.1250T>A	p.L417X	TTA	TAA	A2	HC	Predicted	Tyr	Gln	p.L417Y	p.L417Q
c.1250T>G	p.L417X	TTA	TGA	A2	HC	**Observed**	Trp		p.L417W	
c.1270A>T	p.R424X	AGA	TGA	A2	HC	Predicted	Trp		p.R424W	
c.1279A>T	p.K427X	AAA	TAA	A2	HC	Predicted	Tyr	Gln	p.K427Y	p.K427Q
c.1292T>A	p.L431X	TTG	TAG	A2	HC	Predicted	Tyr		p.L431Y	
c.1306C>T	p.Q436X	CAG	TAG	A2	HC	**Observed**	Tyr		p.Q436Y	
c.1321A>T	p.K441X	AAG	TAG	A2	HC	Predicted	Tyr		p.K441Y	
c.1327A>T	p.K443X	AAA	TAA	A2	HC	Predicted	Tyr	Gln	p.K443Y	p.K443Q
c.1330A>T	p.K444X	AAA	TAA	A2	HC	Predicted	Tyr	Gln	p.K444Y	p.K444Q
c.1336C>T	p.R446X	CGA	TGA	A2	HC	**Observed**	Trp		p.R446W	
c.1357G>T	p.E453X	GAA	TAA	A2	HC	**Observed**	Tyr	Gln	p.E453Y	p.E453Q
c.1366A>T	p.K456X	AAG	TAG	A2	HC	Predicted	Tyr		p.K456Y	
c.1375G>T	p.E459X	GAA	TAA	A2	HC	Predicted	Tyr	Gln	p.E459Y	p.E459Q
c.1384C>T	p.Q462X	CAG	TAG	A2	HC	**Observed**	Tyr		p.Q462Y	
c.1390G>T	p.E464X	GAA	TAA	A2	HC	**Observed**	Tyr	Gln	p.E464Y	p.E464Q
c.1394C>A	p.S465X	TCA	TAA	A2	HC	Predicted	Tyr	Gln	p.S465Y	p.S465Q
c.1394C>G	p.S465X	TCA	TGA	A2	HC	**Observed**	Trp		p.S465W	
c.1396G>T	p.G466X	GGA	TGA	A2	HC	Predicted	Trp		p.G466W	
c.1403T>A	p.L468X	TTG	TAG	A2	HC	Predicted	Tyr		p.L468Y	
c.1405G>T	p.G469X	GGA	TGA	A2	HC	Predicted	Trp		p.G469W	
c.1412T>A	p.L471X	TTA	TAA	A2	HC	Predicted	Tyr	Gln	p.L471Y	p.L471Q
c.1412T>G	p.L471X	TTA	TGA	A2	HC	Predicted	Trp		p.L471W	
c.1423G>T	p.E475X	GAA	TAA	A2	HC	Predicted	Tyr	Gln	p.E475Y	p.E475Q
c.1429G>T	p.G477X	GGA	TGA	A2	HC	Predicted	Trp		p.G477W	
c.1442T>A	p.L481X	TTG	TAG	A2	HC	Predicted	Tyr		p.L481Y	
c.1453A>T	p.K485X	AAG	TAG	A2	HC	**Observed**	Tyr		p.K485Y	
c.1468A>T	p.R490X	AGA	TGA	A2	HC	Predicted	Trp		p.R490W	
c.1492G>T	p.G498X	GGA	TGA	A2	HC	**Observed**	Trp		p.G498W	
c.1514T>A	p.L505X	TTG	TAG	A2	HC	Predicted	Tyr		p.L505Y	
c.1520C>A	p.S507X	TCA	TAA	A2	HC	Predicted	Tyr	Gln	p.S507Y	p.S507Q
c.1520C>G	p.S507X	TCA	TGA	A2	HC	**Observed**	Trp		p.S507W	
c.1525A>T	p.R509X	AGA	TGA	A2	HC	**Observed**	Trp		p.R509W	
c.1529T>A	p.L510X	TTA	TAA	A2	HC	**Observed**	Tyr	Gln	p.L510Y	p.L510Q
c.1529T>G	p.L510X	TTA	TGA	A2	HC	Predicted	Trp		p.L510W	
c.1534A>T	p.K512X	AAA	TAA	A2	HC	Predicted	Tyr	Gln	p.K512Y	p.K512Q
c.1543A>T	p.K515X	AAA	TAA	A2	HC	Predicted	Tyr	Gln	p.K515Y	p.K515Q
c.1550T>A	p.L517X	TTG	TAG	A2	HC	Predicted	Tyr		p.L517Y	
c.1552A>T	p.K518X	AAG	TAG	A2	HC	Predicted	Tyr		p.K518Y	
c.1573G>T	p.G525X	GGA	TGA	A2	HC	Predicted	Trp		p.G525W	
c.1576G>T	p.E526X	GAA	TAA	A2	HC	Predicted	Tyr	Gln	p.E526Y	p.E526Q
c.1585A>T	p.K529X	AAA	TAA	A2	HC	Predicted	Tyr	Gln	p.K529Y	p.K529Q
c.1591A>T	p.K531X	AAA	TAA	A2	HC	Predicted	Tyr	Gln	p.K531Y	p.K531Q
c.1595G>A	p.W532X	TGG	TAG	A2	HC	**Observed**	Tyr		p.W532Y	
c.1609G>T	p.E537X	GAA	TAA	A2	HC	Predicted	Tyr	Gln	p.E537Y	p.E537Q
c.1624A>T	p.K542X	AAA	TAA	A2	HC	Predicted	Tyr	Gln	p.K542Y	p.K542Q
c.1628C>A	p.S543X	TCA	TAA	A2	HC	Predicted	Tyr	Gln	p.S543Y	p.S543Q
c.1628C>G	p.S543X	TCA	TGA	A2	HC	**Observed**	Trp		p.S543W	
c.1641C>A	p.C547X	TGC	TGA	A2	HC	Predicted	Trp		p.C547W	
c.1675G>T	p.E559X	GAG	TAG	A2	HC	**Observed**	Tyr		p.E559Y	
c.1678A>T	p.R560X	AGA	TGA	A2	HC	Predicted	Trp		p.R560W	
c.1691C>G	p.S564X	TCA	TGA	A2	HC	Predicted	Trp		p.S564W	
c.1691C>A	p.S564X	TCA	TAA	A2	HC	Predicted	Tyr	Gln	p.S564Y	p.S564Q
c.1693G>T	p.G565X	GGA	TGA	A2	HC	Predicted	Trp		p.G565W	
c.1719C>A	p.C573X	TGC	TGA	A2	HC	Predicted	Trp		p.C573W	
c.1723A>T	p.K575X	AAA	TAA	A2	HC	Predicted	Tyr	Gln	p.K575Y	p.K575Q
c.1726G>T	p.E576X	GAA	TAA	A2	HC	**Observed**	Tyr	Gln	p.E576Y	p.E576Q
c.1741A>T	p.R581X	AGA	TGA	A2	HC	Predicted	Trp		p.R581W	
c.1744G>T	p.G582X	GGA	TGA	A2	HC	Predicted	Trp		p.G582W	
c.1750C>T	p.Q584X	CAG	TAG	A2	HC	Predicted	Tyr		p.Q584Y	
c.1760C>A	p.S587X	TCA	TAA	A2	HC	**Observed**	Tyr	Gln	p.S587Y	p.S587Q
c.1760C>G	p.S587X	TCA	TGA	A2	HC	Predicted	Trp		p.S587W	
c.1765A>T	p.K589X	AAG	TAG	A2	HC	Predicted	Tyr		p.K589Y	
c.1798G>T	p.E600X	GAG	TAG	A2	HC	**Observed**	Tyr		p.E600Y	
c.1804C>T	p.R602X	CGA	TGA	A2	HC	**Observed**	Trp		p.R602W	
c.1811G>A	p.W604X	TGG	TAG	A2	HC	**Observed**	Tyr		p.W604Y	
c.1822G>T	p.E608X	GAG	TAG	A2	HC	Predicted	Tyr		p.E608Y	
c.1855G>T	p.G619X	GGA	TGA	A2	HC	Predicted	Trp		p.G619W	
c.1861C>T	p.Q621X	CAG	TAG	A2	HC	**Observed**	Tyr		p.Q621Y	
c.1867G>T	p.E623X	GAG	TAG	A2	HC	Predicted	Tyr		p.E623Y	
c.1876G>T	p.E626X	GAG	TAG	A2	HC	Predicted	Tyr		p.E626Y	
c.1931T>A	p.L644X	TTG	TAG	A2	HC	Predicted	Tyr		p.L644Y	
c.1933C>T	p.Q645X	CAG	TAG	A2	HC	**Observed**	Tyr		p.Q645Y	
c.1937T>A	p.L646X	TTG	TAG	A2	HC	Predicted	Tyr		p.L646Y	
c.1940C>A	p.S647X	TCA	TAA	A2	HC	Predicted	Tyr	Gln	p.S647Y	p.S647Q
c.1940C>G	p.S647X	TCA	TGA	A2	HC	Predicted	Trp		p.S647W	
c.1947T>A	p.C649X	TGT	TGA	A2	HC	Predicted	Trp		p.C649W	
c.1949T>A	p.L650X	TTG	TAG	A2	HC	Predicted	Tyr		p.L650Y	
c.1954G>T	p.E652X	GAG	TAG	A2	HC	Predicted	Tyr		p.E652Y	
c.1967G>A	p.W656X	TGG	TAG	A2	HC	**Observed**	Tyr		p.W656Y	
c.1984G>T	p.G662X	GGA	TGA	A2	HC	Predicted	Trp		p.G662W	
c.1990C>T	p.Q664X	CAG	TAG	A2	HC	Predicted	Tyr		p.Q664Y	
c.2020G>T	p.G674X	GGA	TGA	A2	HC	**Observed**	Trp		p.G674W	
c.2032A>T	p.K678X	AAA	TAA	A2	HC	**Observed**	Tyr	Gln	p.K678Y	p.K678Q
c.2038A>T	p.K680X	AAA	TAA	A2	HC	Predicted	Tyr	Gln	p.K680Y	p.K680Q
c.2050G>T	p.E684X	GAA	TAA	A2	HC	Predicted	Tyr	Gln	p.E684Y	p.E684Q
c.2078C>A	p.S693X	TCA	TAA	A2	HC	Predicted	Tyr	Gln	p.S693Y	p.S693Q
c.2078C>G	p.S693X	TCA	TGA	A2	HC	**Observed**	Trp		p.S693W	
c.2080G>T	p.G694X	GGA	TGA	A2	HC	Predicted	Trp		p.G694W	
c.2083G>T	p.E695X	GAA	TAA	A2	HC	Predicted	Tyr	Gln	p.E695Y	p.E695Q
c.2099C>A	p.S700X	TCG	TAG	A2	HC	**Observed**	Tyr		p.S700Y	
c.2104G>T	p.E702X	GAA	TAA	A2	HC	Predicted	Tyr	Gln	p.E702Y	p.E702Q
c.2120G>A	p.W707X	TGG	TAG	A2	HC	**Observed**	Tyr		p.W707Y	
c.2133C>A	p.C711X	TGC	TGA	A2	HC	**Observed**	Trp		p.C711W	
c.2141C>G	p.S714X	TCA	TGA	A2	HC	Predicted	Trp		p.S714W	
c.2141C>A	p.S714X	TCA	TAA	A2	HC	Predicted	Tyr	Gln	p.S714Y	p.S714Q
c.2155A>T	p.R719X	AGA	TGA	A2	HC	Predicted	Trp		p.R719W	
c.2171T>A	p.L724X	TTA	TAA	A2	HC	Predicted	Tyr	Gln	p.L724Y	p.L724Q
c.2171T>G	p.L724X	TTA	TGA	A2	HC	Predicted	Trp		p.L724W	
c.2176A>T	p.K726X	AAG	TAG	A2	HC	Predicted	Tyr		p.K726Y	
c.2190T>A	p.C730X	TGT	TGA	a2	HC	Predicted	Trp		p.C730W	
c.2194A>T	p.K732X	AAG	TAG	a2	HC	Predicted	Tyr		p.K732Y	
c.2215G>T	p.E739X	GAG	TAG	a2	HC	**Observed**	Tyr		p.E739Y	
c.2227G>T	p.E743X	GAA	TAA	a2	HC	Predicted	Tyr	Gln	p.E743Y	p.E743Q
c.2237C>A	p.S746X	TCA	TAA	a2	HC	Predicted	Tyr	Gln	p.S746Y	p.S746Q
c.2237C>G	p.S746X	TCA	TGA	a2	HC	Predicted	Trp		p.S746W	
c.2246T>A	p.L749X	TTG	TAG	a2	HC	**Observed**	Tyr		p.L749Y	
c.2254A>T	p.K752X	AAA	TAA	a2	HC	Predicted	Tyr	Gln	p.K752Y	p.K752Q
c.2269G>T	p.E757X	GAA	TAA	a2	HC	Predicted	Tyr	Gln	p.E757Y	p.E757Q
c.2275A>T	p.R759X	AGA	TGA	a2	HC	Predicted	Trp		p.R759W	
c.2287C>T	p.Q763X	CAG	TAG	B	B	Predicted	Tyr		p.Q763Y	
c.2294C>A	p.S765X	TCA	TAA	B	B	Predicted	Tyr	Gln	p.S765Y	p.S765Q
c.2294C>G	p.S765X	TCA	TGA	B	B	Predicted	Trp		p.S765W	
c.2296A>T	p.R766X	AGA	TGA	B	B	Predicted	Trp		p.R766W	
c.2317A>T	p.K773X	AAG	TAG	B	B	Predicted	Tyr		p.K773Y	
c.2344G>T	p.E782X	GAA	TAA	B	B	Predicted	Tyr	Gln	p.E782Y	p.E782Q
c.2356G>T	p.E786X	GAG	TAG	B	B	Predicted	Tyr		p.E786Y	
c.2359A>T	p.K787X	AAG	TAG	B	B	Predicted	Tyr		p.K787Y	
c.2372G>A	p.W791X	TGG	TAG	B	B	**Observed**	Tyr		p.W791Y	
c.2383A>T	p.R795X	AGA	TGA	B	B	Predicted	Trp		p.R795W	
c.2398A>T	p.K800X	AAA	TAA	B	B	Predicted	Tyr	Gln	p.K800Y	p.K800Q
c.2426T>A	p.L809X	TTG	TAG	B	B	Predicted	Tyr		p.L809Y	
c.2429T>A	p.L810X	TTG	TAG	B	B	Predicted	Tyr		p.L810Y	
c.2438T>A	p.L813X	TTG	TAG	B	B	Predicted	Tyr		p.L813Y	
c.2440C>T	p.R814X	CGA	TGA	B	B	**Observed**	Trp		p.R814W	
c.2443C>T	p.Q815X	CAG	TAG	B	B	**Observed**	Tyr		p.Q815Y	
c.2471T>A	p.L824X	TTA	TAA	B	B	Predicted	Tyr	Gln	p.L824Y	p.L824Q
c.2471T>G	p.L824X	TTA	TGA	B	B	Predicted	Trp		p.L824W	
c.2485G>T	p.E829X	GAA	TAA	B	B	**Observed**	Tyr	Gln	p.E829Y	p.E829Q
c.2491A>T	p.K831X	AAA	TAA	B	B	Predicted	Tyr	Gln	p.K831Y	p.K831Q
c.2497G>T	p.E833X	GAG	TAG	B	B	Predicted	Tyr		p.E833Y	
c.2519C>A	p.S840X	TCA	TAA	B	B	Predicted	Tyr	Gln	p.S840Y	p.S840Q
c.2519C>G	p.S840X	TCA	TGA	B	B	Predicted	Trp		p.S840W	
c.2524G>T	p.G842X	GGA	TGA	B	B	Predicted	Trp		p.G842W	
c.2554G>T	p.E852X	GAA	TAA	B	B	Predicted	Tyr	Gln	p.E852Y	p.E852Q
c.2575C>T	p.Q859X	CAG	TAG	B	B	**Observed**	Tyr		p.Q859Y	
c.2611G>T	p.E871X	GAG	TAG	B	B	Predicted	Tyr		p.E871Y	
c.2615C>A	p.S872X	TCA	TAA	B	B	**Observed**	Tyr	Gln	p.S872Y	p.S872Q
c.2615C>G	p.S872X	TCA	TGA	B	B	**Observed**	Trp		p.S872W	
c.2627T>G	p.L876X	TTA	TGA	B	B	Predicted	Trp		p.L876W	
c.2627T>A	p.L876X	TTA	TAA	B	B	Predicted	Tyr	Gln	p.L876Y	p.L876Q
c.2629A>T	p.R877X	AGA	TGA	B	B	Predicted	Trp		p.R877W	
c.2633T>A	p.L878X	TTA	TAA	B	B	Predicted	Tyr	Gln	p.L878Y	p.L878Q
c.2633T>G	p.L878X	TTA	TGA	B	B	**Observed**	Trp		p.L878W	
c.2638G>T	p.E880X	GAG	TAG	B	B	**Observed**	Tyr		p.E880Y	
c.2641A>T	p.K881X	AAA	TAA	B	B	Predicted	Tyr	Gln	p.K881Y	p.K881Q
c.2665G>T	p.E889X	GAG	TAG	B	B	Predicted	Tyr		p.E889Y	
c.2669T>A	p.L890X	TTG	TAG	B	B	Predicted	Tyr		p.L890Y	
c.2671A>T	p.K891X	AAG	TAG	B	B	Predicted	Tyr		p.K891Y	
c.2674A>T	p.K892X	AAA	TAA	B	B	Predicted	Tyr	Gln	p.K892Y	p.K892Q
c.2686A>T	p.K896X	AAA	TAA	B	B	Predicted	Tyr	Gln	p.K896Y	p.K896Q
c.2702C>A	p.S901X	TCA	TAA	B	B	**Observed**	Tyr	Gln	p.S901Y	p.S901Q
c.2702C>G	p.S901X	TCA	TGA	B	B	**Observed**	Trp		p.S901W	
c.2717C>G	p.S906X	TCA	TGA	B	B	Predicted	Trp		p.S906W	
c.2717C>A	p.S906X	TCA	TAA	B	B	Predicted	Tyr	Gln	p.S906Y	p.S906Q
c.2729C>G	p.S910X	TCA	TGA	B	B	Predicted	Trp		p.S910W	
c.2729C>A	p.S910X	TCA	TAA	B	B	Predicted	Tyr	Gln	p.S910Y	p.S910Q
c.2738T>A	p.L913X	TTG	TAG	B	B	Predicted	Tyr		p.L913Y	
c.2768T>A	p.L923X	TTA	TAA	B	B	Predicted	Tyr	Gln	p.L923Y	p.L923Q
c.2768T>G	p.L923X	TTA	TGA	B	B	**Observed**	Trp		p.L923W	
c.2770G>T	p.G924X	GGA	TGA	B	B	**Observed**	Trp		p.G924W	
c.2807T>A	p.L936X	TTA	TAA	B	B	Predicted	Tyr	Gln	p.L936Y	p.L936Q
c.2807T>G	p.L936X	TTA	TGA	B	B	Predicted	Trp		p.L936W	
c.2827A>T	p.K943X	AAA	TAA	B	B	Predicted	Tyr	Gln	p.K943Y	p.K943Q
c.2830A>T	p.K944X	AAG	TAG	B	B	Predicted	Tyr		p.K944Y	
c.2834C>A	p.S945X	TCA	TAA	B	B	Predicted	Tyr	Gln	p.S945Y	p.S945Q
c.2834C>G	p.S945X	TCA	TGA	B	B	Predicted	Trp		p.S945W	
c.2848G>T	p.E950X	GAG	TAG	B	B	Predicted	Tyr		p.E950Y	
c.2857G>T	p.G953X	GGA	TGA	B	B	**Observed**	Trp		p.G953W	
c.2870T>A	p.L957X	TTG	TAG	B	B	Predicted	Tyr		p.L957Y	
c.2875G>T	p.E959X	GAA	TAA	B	B	Predicted	Tyr	Gln	p.E959Y	p.E959Q
c.2878G>T	p.E960X	GAA	TAA	B	B	Predicted	Tyr	Gln	p.E960Y	p.E960Q
c.2891C>A	p.S964X	TCA	TAA	B	B	Predicted	Tyr	Gln	p.S964Y	p.S964Q
c.2891C>G	p.S964X	TCA	TGA	B	B	Predicted	Trp		p.S964W	
c.2893A>T	p.K965X	AAG	TAG	B	B	**Observed**	Tyr		p.K965Y	
c.2897T>A	p.L966X	TTG	TAG	B	B	Predicted	Tyr		p.L966Y	
c.2900T>A	p.L967X	TTA	TAA	B	B	Predicted	Tyr	Gln	p.L967Y	p.L967Q
c.2900T>G	p.L967X	TTA	TGA	B	B	Predicted	Trp		p.L967W	
c.2902G>T	p.E968X	GAA	TAA	B	B	**Observed**	Tyr	Gln	p.E968Y	p.E968Q
c.2906C>A	p.S969X	TCA	TAA	B	B	Predicted	Tyr	Gln	p.S969Y	p.S969Q
c.2906C>G	p.S969X	TCA	TGA	B	B	Predicted	Trp		p.S969W	
c.2912T>A	p.L971X	TTA	TAA	B	B	Predicted	Tyr	Gln	p.L971Y	p.L971Q
c.2912T>G	p.L971X	TTA	TGA	B	B	**Observed**	Trp		p.L971W	
c.2926G>T	p.E976X	GAA	TAA	B	B	Predicted	Tyr	Gln	p.E976Y	p.E976Q
c.2933C>A	p.S978X	TCA	TAA	B	B	**Observed**	Tyr	Gln	p.S978Y	p.S978Q
c.2933C>G	p.S978X	TCA	TGA	B	B	Predicted	Trp		p.S978W	
c.2936G>A	p.W979X	TGG	TAG	B	B	**Observed**	Tyr		p.W979Y	
c.2938G>T	p.G980X	GGA	TGA	B	B	Predicted	Trp		p.G980W	
c.2941A>T	p.K981X	AAA	TAA	B	B	Predicted	Tyr	Gln	p.K981Y	p.K981Q
c.2951C>A	p.S984X	TCG	TAG	B	B	Predicted	Tyr		p.S984Y	
c.2954C>A	p.S985X	TCA	TAA	B	B	**Observed**	Tyr	Gln	p.S985Y	p.S985Q
c.2954C>G	p.S985X	TCA	TGA	B	B	Predicted	Trp		p.S985W	
c.2959G>T	p.E987X	GAG	TAG	B	B	Predicted	Tyr		p.E987Y	
c.2972T>A	p.L991X	TTA	TAA	B	B	Predicted	Tyr	Gln	p.L991Y	p.L991Q
c.2972T>G	p.L991X	TTA	TGA	B	B	Predicted	Trp		p.L991W	
c.2977A>T	p.K993X	AAA	TAA	B	B	**Observed**	Tyr	Gln	p.K993Y	p.K993Q
c.2983A>T	p.K995X	AAA	TAA	B	B	Predicted	Tyr	Gln	p.K995Y	p.K995Q
c.2986A>T	p.R996X	AGA	TGA	B	B	Predicted	Trp		p.R996W	
c.2995G>T	p.G999X	GGA	TGA	B	B	Predicted	Trp		p.G999W	
c.3005T>A	p.L1002X	TTG	TAG	B	B	Predicted	Tyr		p.L1002Y	
c.3008T>A	p.L1003X	TTG	TAG	B	B	Predicted	Tyr		p.L1003Y	
c.3013A>T	p.K1005X	AAA	TAA	B	B	Predicted	Tyr	Gln	p.K1005Y	p.K1005Q
c.3026T>A	p.L1009X	TTA	TAA	B	B	Predicted	Tyr	Gln	p.L1009Y	p.L1009Q
c.3026T>G	p.L1009X	TTA	TGA	B	B	Predicted	Trp		p.L1009W	
c.3031A>T	p.K1011X	AAA	TAA	B	B	**Observed**	Tyr	Gln	p.K1011Y	p.K1011Q
c.3047T>A	p.L1016X	TTG	TAG	B	B	Predicted	Tyr		p.L1016Y	
c.3050T>A	p.L1017X	TTA	TAA	B	B	Predicted	Tyr	Gln	p.L1017Y	p.L1017Q
c.3050T>G	p.L1017X	TTA	TGA	B	B	Predicted	Trp		p.L1017W	
c.3052A>T	p.K1018X	AAG	TAG	B	B	Predicted	Tyr		p.K1018Y	
c.3061A>T	p.K1021X	AAA	TAA	B	B	Predicted	Tyr	Gln	p.K1021Y	p.K1021Q
c.3077C>A	p.S1026X	TCA	TAA	B	B	Predicted	Tyr	Gln	p.S1026Y	p.S1026Q
c.3077C>G	p.S1026X	TCA	TGA	B	B	Predicted	Trp		p.S1026W	
c.3088A>T	p.R1030X	AGA	TGA	B	B	Predicted	Trp		p.R1030W	
c.3091A>T	p.K1031X	AAG	TAG	B	B	Predicted	Tyr		p.K1031Y	
c.3113C>A	p.S1038X	TCA	TAA	B	B	**Observed**	Tyr	Gln	p.S1038Y	p.S1038Q
c.3113C>G	p.S1038X	TCA	TGA	B	B	**Observed**	Trp		p.S1038W	
c.3116T>A	p.L1039X	TTA	TAA	B	B	Predicted	Tyr	Gln	p.L1039Y	p.L1039Q
c.3116T>G	p.L1039X	TTA	TGA	B	B	Predicted	Trp		p.L1039W	
c.3119T>A	p.L1040X	TTA	TAA	B	B	Predicted	Tyr	Gln	p.L1040Y	p.L1040Q
c.3119T>G	p.L1040X	TTA	TGA	B	B	Predicted	Trp		p.L1040W	
c.3124G>T	p.E1042X	GAG	TAG	B	B	Predicted	Tyr		p.E1042Y	
c.3137C>A	p.S1046X	TCA	TAA	B	B	Predicted	Tyr	Gln	p.S1046Y	p.S1046Q
c.3137C>G	p.S1046X	TCA	TGA	B	B	Predicted	Trp		p.S1046W	
c.3143G>A	p.W1048X	TGG	TAG	B	B	**Observed**	Tyr		p.W1048Y	
c.3155T>A	p.L1052X	TTA	TAA	B	B	**Observed**	Tyr	Gln	p.L1052Y	p.L1052Q
c.3155T>G	p.L1052X	TTA	TGA	B	B	Predicted	Trp		p.L1052W	
c.3157G>T	p.E1053X	GAA	TAA	B	B	**Observed**	Tyr	Gln	p.E1053Y	p.E1053Q
c.3169G>T	p.E1057X	GAG	TAG	B	B	Predicted	Tyr		p.E1057Y	
c.3175A>T	p.K1059X	AAA	TAA	B	B	**Observed**	Tyr	Gln	p.K1059Y	p.K1059Q
c.3178A>T	p.K1060X	AAA	TAA	B	B	**Observed**	Tyr	Gln	p.K1060Y	p.K1060Q
c.3191T>A	p.L1064X	TTG	TAG	B	B	Predicted	Tyr		p.L1064Y	
c.3202A>T	p.R1068X	AGA	TGA	B	B	Predicted	Trp		p.R1068W	
c.3217A>T	p.K1073X	AAA	TAA	B	B	Predicted	Tyr	Gln	p.K1073Y	p.K1073Q
c.3233T>A	p.L1078X	TTG	TAG	B	B	Predicted	Tyr		p.L1078Y	
c.3251C>A	p.S1084X	TCA	TAA	B	B	Predicted	Tyr	Gln	p.S1084Y	p.S1084Q
c.3251C>G	p.S1084X	TCA	TGA	B	B	**Observed**	Trp		p.S1084W	
c.3256A>T	p.K1086X	AAA	TAA	B	B	Predicted	Tyr	Gln	p.K1086Y	p.K1086Q
c.3266C>A	p.S1089X	TCA	TAA	B	B	Predicted	Tyr	Gln	p.S1089Y	p.S1089Q
c.3266C>G	p.S1089X	TCA	TGA	B	B	Predicted	Trp		p.S1089W	
c.3269C>A	p.S1090X	TCA	TAA	B	B	Predicted	Tyr	Gln	p.S1090Y	p.S1090Q
c.3269C>G	p.S1090X	TCA	TGA	B	B	**Observed**	Trp		p.S1090W	
c.3271A>T	p.K1091X	AAA	TAA	B	B	Predicted	Tyr	Gln	p.K1091Y	p.K1091Q
c.3280G>T	p.E1094X	GAA	TAA	B	B	Predicted	Tyr	Gln	p.E1094Y	p.E1094Q
c.3292C>T	p.Q1098X	CAG	TAG	B	B	**Observed**	Tyr		p.Q1098Y	
c.3295A>T	p.K1099X	AAA	TAA	B	B	Predicted	Tyr	Gln	p.K1099Y	p.K1099Q
c.3298A>T	p.K1100X	AAA	TAA	B	B	**Observed**	Tyr	Gln	p.K1100Y	p.K1100Q
c.3301G>T	p.E1101X	GAG	TAG	B	B	Predicted	Tyr		p.E1101Y	
c.3341C>A	p.S1114X	TCG	TAG	B	B	Predicted	Tyr		p.S1114Y	
c.3349A>T	p.K1117X	AAG	TAG	B	B	Predicted	Tyr		p.K1117Y	
c.3362T>A	p.L1121X	TTG	TAG	B	B	Predicted	Tyr		p.L1121Y	
c.3367G>T	p.E1123X	GAA	TAA	B	B	Predicted	Tyr	Gln	p.E1123Y	p.E1123Q
c.3371C>A	p.S1124X	TCA	TAA	B	B	**Observed**	Tyr	Gln	p.S1124Y	p.S1124Q
c.3371C>G	p.S1124X	TCA	TGA	B	B	Predicted	Trp		p.S1124W	
c.3380G>A	p.W1127X	TGG	TAG	B	B	Predicted	Tyr		p.W1127Y	
c.3397G>T	p.G1133X	GGA	TGA	B	B	Predicted	Trp		p.G1133W	
c.3400A>T	p.K1134X	AAG	TAG	B	B	Predicted	Tyr		p.K1134Y	
c.3436A>T	p.K1146X	AAG	TAG	B	B	Predicted	Tyr		p.K1146Y	
c.3443T>A	p.L1148X	TTA	TAA	B	B	Predicted	Tyr	Gln	p.L1148Y	p.L1148Q
c.3443T>G	p.L1148X	TTA	TGA	B	B	Predicted	Trp		p.L1148W	
c.3452T>A	p.L1151X	TTA	TAA	B	B	Predicted	Tyr	Gln	p.L1151Y	p.L1151Q
c.3452T>G	p.L1151X	TTA	TGA	B	B	Predicted	Trp		p.L1151W	
c.3454G>T	p.G1152X	GGA	TGA	B	B	Predicted	Trp		p.G1152W	
c.3460G>T	p.E1154X	GAA	TAA	B	B	Predicted	Tyr	Gln	p.E1154Y	p.E1154Q
c.3463A>T	p.K1155X	AAA	TAA	B	B	Predicted	Tyr	Gln	p.K1155Y	p.K1155Q
c.3472G>T	p.E1158X	GAA	TAA	B	B	Predicted	Tyr	Gln	p.E1158Y	p.E1158Q
c.3478C>T	p.Q1160X	CAG	TAG	B	B	**Observed**	Tyr		p.Q1160Y	
c.3488T>A	p.L1163X	TTG	TAG	B	B	Predicted	Tyr		p.L1163Y	
c.3493G>T	p.E1165X	GAG	TAG	B	B	**Observed**	Tyr		p.E1165Y	
c.3496A>T	p.K1166X	AAA	TAA	B	B	**Observed**	Tyr	Gln	p.K1166Y	p.K1166Q
c.3502A>T	p.K1168X	AAA	TAA	B	B	Predicted	Tyr	Gln	p.K1168Y	p.K1168Q
c.3514G>T	p.G1172X	GGA	TGA	B	B	Predicted	Trp		p.G1172W	
c.3517A>T	p.K1173X	AAG	TAG	B	B	Predicted	Tyr		p.K1173Y	
c.3523G>T	p.E1175X	GAA	TAA	B	B	Predicted	Tyr	Gln	p.E1175Y	p.E1175Q
c.3532A>T	p.K1178X	AAG	TAG	B	B	Predicted	Tyr		p.K1178Y	
c.3541G>T	p.G1181X	GGA	TGA	B	B	Predicted	Trp		p.G1181W	
c.3547A>T	p.K1183X	AAA	TAA	B	B	Predicted	Tyr	Gln	p.K1183Y	p.K1183Q
c.3550G>T	p.E1184X	GAG	TAG	B	B	Predicted	Tyr		p.E1184Y	
c.3571A>T	p.R1191X	AGA	TGA	B	B	Predicted	Trp		p.R1191W	
c.3593T>A	p.L1198X	TTG	TAG	B	B	Predicted	Tyr		p.L1198Y	
c.3602T>A	p.L1201X	TTA	TAA	B	B	Predicted	Tyr	Gln	p.L1201Y	p.L1201Q
c.3602T>G	p.L1201X	TTA	TGA	B	B	Predicted	Trp		p.L1201W	
c.3607G>T	p.E1203X	GAA	TAA	B	B	**Observed**	Tyr	Gln	p.E1203Y	p.E1203Q
c.3628G>T	p.E1210X	GAA	TAA	B	B	Predicted	Tyr	Gln	p.E1210Y	p.E1210Q
c.3631A>T	p.K1211X	AAA	TAA	B	B	**Observed**	Tyr	Gln	p.K1211Y	p.K1211Q
c.3634A>T	p.K1212X	AAA	TAA	B	B	**Observed**	Tyr	Gln	p.K1212Y	p.K1212Q
c.3640C>T	p.Q1214X	CAG	TAG	B	B	**Observed**	Tyr		p.Q1214Y	
c.3643G>T	p.E1215X	GAA	TAA	B	B	Predicted	Tyr	Gln	p.E1215Y	p.E1215Q
c.3646G>T	p.E1216X	GAA	TAA	B	B	**Observed**	Tyr	Gln	p.E1216Y	p.E1216Q
c.3652G>T	p.E1218X	GAA	TAA	B	B	Predicted	Tyr	Gln	p.E1218Y	p.E1218Q
c.3655A>T	p.K1219X	AAG	TAG	B	B	Predicted	Tyr		p.K1219Y	
c.3658A>T	p.K1220X	AAG	TAG	B	B	Predicted	Tyr		p.K1220Y	
c.3661G>T	p.E1221X	GAA	TAA	B	B	Predicted	Tyr	Gln	p.E1221Y	p.E1221Q
c.3668T>A	p.L1223X	TTA	TAA	B	B	**Observed**	Tyr	Gln	p.L1223Y	p.L1223Q
c.3668T>G	p.L1223X	TTA	TGA	B	B	Predicted	Trp		p.L1223W	
c.3676G>T	p.E1226X	GAG	TAG	B	B	Predicted	Tyr		p.E1226Y	
c.3689T>A	p.L1230X	TTG	TAG	B	B	Predicted	Tyr		p.L1230Y	
c.3694C>T	p.Q1232X	CAG	TAG	B	B	**Observed**	Tyr		p.Q1232Y	
c.3718A>T	p.K1240X	AAG	TAG	B	B	Predicted	Tyr		p.K1240Y	
c.3730A>T	p.K1244X	AAG	TAG	B	B	Predicted	Tyr		p.K1244Y	
c.3743T>A	p.L1248X	TTA	TAA	B	B	Predicted	Tyr	Gln	p.L1248Y	p.L1248Q
c.3743T>G	p.L1248X	TTA	TGA	B	B	Predicted	Trp		p.L1248W	
c.3766G>T	p.E1256X	GAA	TAA	B	B	**Observed**	Tyr	Gln	p.E1256Y	p.E1256Q
c.3773C>A	p.S1258X	TCA	TAA	B	B	Predicted	Tyr	Gln	p.S1258Y	p.S1258Q
c.3773C>G	p.S1258X	TCA	TGA	B	B	Predicted	Trp		p.S1258W	
c.3815C>G	p.S1272X	TCA	TGA	B	B	Predicted	Trp		p.S1272W	
c.3815C>A	p.S1272X	TCA	TAA	B	B	Predicted	Tyr	Gln	p.S1272Y	p.S1272Q
c.3818T>A	p.L1273X	TTA	TAA	B	B	Predicted	Tyr	Gln	p.L1273Y	p.L1273Q
c.3818T>G	p.L1273X	TTA	TGA	B	B	Predicted	Trp		p.L1273W	
c.3827C>A	p.S1276X	TCA	TAA	B	B	**Observed**	Tyr	Gln	p.S1276Y	p.S1276Q
c.3827C>G	p.S1276X	TCA	TGA	B	B	**Observed**	Trp		p.S1276W	
c.3835A>T	p.R1279X	AGA	TGA	B	B	Predicted	Trp		p.R1279W	
c.3841A>T	p.K1281X	AAG	TAG	B	B	Predicted	Tyr		p.K1281Y	
c.3844A>T	p.K1282X	AAA	TAA	B	B	**Observed**	Tyr	Gln	p.K1282Y	p.K1282Q
c.3863C>A	p.S1288X	TCA	TAA	B	B	Predicted	Tyr	Gln	p.S1288Y	p.S1288Q
c.3863C>G	p.S1288X	TCA	TGA	B	B	Predicted	Trp		p.S1288W	
c.3865A>T	p.K1289X	AAA	TAA	B	B	**Observed**	Tyr	Gln	p.K1289Y	p.K1289Q
c.3868A>T	p.K1290X	AAA	TAA	B	B	**Observed**	Tyr	Gln	p.K1290Y	p.K1290Q
c.3874G>T	p.E1292X	GAG	TAG	B	B	Predicted	Tyr		p.E1292Y	
c.3877G>T	p.E1293X	GAA	TAA	B	B	Predicted	Tyr	Gln	p.E1293Y	p.E1293Q
c.3880G>T	p.E1294X	GAA	TAA	B	B	Predicted	Tyr	Gln	p.E1294Y	p.E1294Q
c.3887T>A	p.L1296X	TTG	TAG	B	B	Predicted	Tyr		p.L1296Y	
c.3889G>T	p.E1297X	GAA	TAA	B	B	Predicted	Tyr	Gln	p.E1297Y	p.E1297Q
c.3896T>A	p.L1299X	TTG	TAG	B	B	Predicted	Tyr		p.L1299Y	
c.3898G>T	p.G1300X	GGA	TGA	B	B	Predicted	Trp		p.G1300W	
c.3910A>T	p.K1304X	AAG	TAG	B	B	Predicted	Tyr		p.K1304Y	
c.3922G>T	p.E1308X	GAG	TAG	B	B	**Observed**	Tyr		p.E1308Y	

c.3925A>T	p.K1309X	AAA	TAA	B	B	Predicted	Tyr	Gln	p.K1309Y	p.K1309Q
c.3936C>A	p.C1312X	TGC	TGA	B	B	Predicted	Trp		p.C1312W	
c.3964C>T	p.Q1322X	CAG	TAG	B	B	**Observed**	Tyr		p.Q1322Y	
c.3967C>T	p.Q1323X	CAG	TAG	B	B	**Observed**	Tyr		p.Q1323Y	
c.3991A>T	p.K1331X	AAG	TAG	B	B	Predicted	Tyr		p.K1331Y	
c.3994A>T	p.R1332X	AGA	TGA	B	B	**Observed**	Trp		p.R1332W	
c.4001T>A	p.L1334X	TTG	TAG	B	B	Predicted	Tyr		p.L1334Y	
c.4003A>T	p.K1335X	AAA	TAA	B	B	Predicted	Tyr	Gln	p.K1335Y	p.K1335Q
c.4012A>T	p.R1338X	AGA	TGA	B	B	Predicted	Trp		p.R1338W	
c.4024G>T	p.E1342X	GAA	TAA	B	B	Predicted	Tyr	Gln	p.E1342Y	p.E1342Q
c.4027G>T	p.E1343X	GAA	TAA	B	B	**Observed**	Tyr	Gln	p.E1343Y	p.E1343Q
c.4033G>T	p.E1345X	GAA	TAA	B	B	Predicted	Tyr	Gln	p.E1345Y	p.E1345Q
c.4039G>T	p.E1347X	GAA	TAA	B	B	**Observed**	Tyr	Gln	p.E1347Y	p.E1347Q
c.4042A>T	p.K1348X	AAA	TAA	B	B	**Observed**	Tyr	Gln	p.K1348Y	p.K1348Q
c.4067C>A	p.S1356X	TCA	TAA	B	B	**Observed**	Tyr	Gln	p.S1356Y	p.S1356Q
c.4067C>G	p.S1356X	TCA	TGA	B	B	Predicted	Trp		p.S1356W	
c.4072C>T	p.Q1358X	CAG	TAG	B	B	**Observed**	Tyr		p.Q1358Y	
c.4076G>A	p.W1359X	TGG	TAG	B	B	**Observed**	Tyr		p.W1359Y	
c.4081A>T	p.K1361X	AAA	TAA	B	B	Predicted	Tyr	Gln	p.K1361Y	p.K1361Q
c.4090A>T	p.K1364X	AAA	TAA	B	B	Predicted	Tyr	Gln	p.K1364Y	p.K1364Q
c.4097T>A	p.L1366X	TTG	TAG	B	B	Predicted	Tyr		p.L1366Y	
c.4117C>T	p.Q1373X	CAG	TAG	B	B	Predicted	Tyr		p.Q1373Y	
c.4132G>T	p.E1378X	GAG	TAG	B	B	Predicted	Tyr		p.E1378Y	
c.4135A>T	p.K1379X	AAG	TAG	B	B	Predicted	Tyr		p.K1379Y	
c.4138G>T	p.E1380X	GAG	TAG	B	B	Predicted	Tyr		p.E1380Y	
c.4141A>T	p.K1381X	AAA	TAA	B	B	Predicted	Tyr	Gln	p.K1381Y	p.K1381Q
c.4156C>T	p.Q1386X	CAG	TAG	B	B	**Observed**	Tyr		p.Q1386Y	
c.4166T>A	p.L1389X	TTA	TAA	B	B	Predicted	Tyr	Gln	p.L1389Y	p.L1389Q
c.4166T>G	p.L1389X	TTA	TGA	B	B	Predicted	Trp		p.L1389W	
c.4169C>A	p.S1390X	TCA	TAA	B	B	Predicted	Tyr	Gln	p.S1390Y	p.S1390Q
c.4169C>G	p.S1390X	TCA	TGA	B	B	**Observed**	Trp		p.S1390W	
c.4176C>A	p.C1392X	TGC	TGA	B	B	Predicted	Trp		p.C1392W	
c.4210A>T	p.R1404X	AGA	TGA	B	B	**Observed**	Trp		p.R1404W	
c.4220T>A	p.L1407X	TTA	TAA	B	B	Predicted	Tyr	Gln	p.L1407Y	p.L1407Q
c.4220T>G	p.L1407X	TTA	TGA	B	B	Predicted	Trp		p.L1407W	
c.4231A>T	p.K1411X	AAG	TAG	B	B	Predicted	Tyr		p.K1411Y	
c.4238C>A	p.S1413X	TCA	TAA	B	B	Predicted	Tyr	Gln	p.S1413Y	p.S1413Q
c.4238C>G	p.S1413X	TCA	TGA	B	B	Predicted	Trp		p.S1413W	
c.4241C>A	p.S1414X	TCA	TAA	B	B	**Observed**	Tyr	Gln	p.S1414Y	p.S1414Q
c.4241C>G	p.S1414X	TCA	TGA	B	B	Predicted	Trp		p.S1414W	
c.4255A>T	p.R1419X	AGA	TGA	B	B	Predicted	Trp		p.R1419W	
c.4321A>T	p.R1441X	AGA	TGA	B	B	Predicted	Trp		p.R1441W	
c.4324A>T	p.K1442X	AAG	TAG	B	B	Predicted	Tyr		p.K1442Y	
c.4327A>T	p.K1443X	AAA	TAA	B	B	Predicted	Tyr	Gln	p.K1443Y	p.K1443Q
c.4345G>T	p.E1449X	GAA	TAA	B	B	**Observed**	Tyr	Gln	p.E1449Y	p.E1449Q
c.4361T>A	p.L1454X	TTA	TAA	B	B	Predicted	Tyr	Gln	p.L1454Y	p.L1454Q
c.4361T>G	p.L1454X	TTA	TGA	B	B	Predicted	Trp		p.L1454W	
c.4366G>T	p.G1456X	GGA	TGA	B	B	Predicted	Trp		p.G1456W	
c.4372A>T	p.K1458X	AAA	TAA	B	B	Predicted	Tyr	Gln	p.K1458Y	p.K1458Q
c.4375A>T	p.K1459X	AAA	TAA	B	B	Predicted	Tyr	Gln	p.K1459Y	p.K1459Q
c.4391T>A	p.L1464X	TTA	TAA	B	B	Predicted	Tyr	Gln	p.L1464Y	p.L1464Q
c.4391T>G	p.L1464X	TTA	TGA	B	B	Predicted	Trp		p.L1464W	
c.4406T>A	p.L1469X	TTG	TAG	B	B	Predicted	Tyr		p.L1469Y	
c.4408G>T	p.E1470X	GAG	TAG	B	B	**Observed**	Tyr		p.E1470Y	
c.4426A>T	p.R1476X	AGA	TGA	B	B	Predicted	Trp		p.R1476W	
c.4429G>T	p.E1477X	GAG	TAG	B	B	Predicted	Tyr		p.E1477Y	
c.4463C>A	p.S1488X	TCA	TAA	B	B	Predicted	Tyr	Gln	p.S1488Y	p.S1488Q
c.4463C>G	p.S1488X	TCA	TGA	B	B	Predicted	Trp		p.S1488W	
c.4474A>T	p.K1492X	AAG	TAG	B	B	**Observed**	Tyr		p.K1492Y	
c.4477A>T	p.K1493X	AAA	TAA	B	B	Predicted	Tyr	Gln	p.K1493Y	p.K1493Q
c.4483G>T	p.E1495X	GAG	TAG	B	B	**Observed**	Tyr		p.E1495Y	
c.4501A>T	p.K1501X	AAA	TAA	B	B	Predicted	Tyr	Gln	p.K1501Y	p.K1501Q
c.4511T>A	p.L1504X	TTG	TAG	B	B	Predicted	Tyr		p.L1504Y	
c.4516A>T	p.K1506X	AAA	TAA	B	B	**Observed**	Tyr	Gln	p.K1506Y	p.K1506Q
c.4528A>T	p.K1510X	AAA	TAA	B	B	Predicted	Tyr	Gln	p.K1510Y	p.K1510Q
c.4534G>T	p.E1512X	GAA	TAA	B	B	**Observed**	Tyr	Gln	p.E1512Y	p.E1512Q
c.4538T>A	p.L1513X	TTG	TAG	B	B	Predicted	Tyr		p.L1513Y	
c.4546A>T	p.K1516X	AAA	TAA	B	B	Predicted	Tyr	Gln	p.K1516Y	p.K1516Q
c.4561C>T	p.Q1521X	CAG	TAG	B	B	**Observed**	Tyr		p.Q1521Y	
c.4564A>T	p.K1522X	AAG	TAG	B	B	**Observed**	Tyr		p.K1522Y	
c.4582G>T	p.E1528X	GAA	TAA	B	B	Predicted	Tyr	Gln	p.E1528Y	p.E1528Q
c.4621G>T	p.E1541X	GAA	TAA	B	B	Predicted	Tyr	Gln	p.E1541Y	p.E1541Q
c.4636C>T	p.Q1546X	CAG	TAG	B	B	Predicted	Tyr		p.Q1546Y	
c.4639G>T	p.G1547X	GGA	TGA	B	B	Predicted	Trp		p.G1547W	
c.4645G>T	p.E1549X	GAG	TAG	B	B	Predicted	Tyr		p.E1549Y	
c.4648G>T	p.G1550X	GGA	TGA	B	B	Predicted	Trp		p.G1550W	
c.4657A>T	p.K1553X	AAG	TAG	B	B	Predicted	Tyr		p.K1553Y	
c.4661G>A	p.W1554X	TGG	TAG	B	B	**Observed**	Tyr		p.W1554Y	
c.4666G>T	p.E1556X	GAA	TAA	B	B	Predicted	Tyr	Gln	p.E1556Y	p.E1556Q
c.4675A>T	p.R1559X	AGA	TGA	B	B	Predicted	Trp		p.R1559W	
c.4681G>T	p.G1561X	GGA	TGA	B	B	Predicted	Trp		p.G1561W	
c.4684A>T	p.K1562X	AAA	TAA	B	B	**Observed**	Tyr	Gln	p.K1562Y	p.K1562Q
c.4699A>T	p.R1567X	AGA	TGA	B	B	Predicted	Trp		p.R1567W	
c.4711G>T	p.E1571X	GAA	TAA	B	B	Predicted	Tyr	Gln	p.E1571Y	p.E1571Q
c.4723A>T	p.K1575X	AAG	TAG	B	B	Predicted	Tyr		p.K1575Y	
c.4735A>T	p.K1579X	AAG	TAG	B	B	Predicted	Tyr		p.K1579Y	
c.4742T>A	p.L1581X	TTG	TAG	B	B	Predicted	Tyr		p.L1581Y	
c.4757G>A	p.W1586X	TGG	TAG	B	B	**Observed**	Tyr		p.W1586Y	
c.4777C>T	p.Q1593X	CAG	TAG	B	B	Predicted	Tyr		p.Q1593Y	
c.4786A>T	p.K1596X	AAA	TAA	B	B	Predicted	Tyr	Gln	p.K1596Y	p.K1596Q
c.4789G>T	p.E1597X	GAA	TAA	B	B	**Observed**	Tyr	Gln	p.E1597Y	p.E1597Q
c.4792G>T	p.E1598X	GAG	TAG	B	B	Predicted	Tyr		p.E1598Y	
c.4796G>A	p.W1599X	TGG	TAG	B	B	**Observed**	Tyr		p.W1599Y	
c.4798A>T	p.K1600X	AAA	TAA	B	B	**Observed**	Tyr	Gln	p.K1600Y	p.K1600Q
c.4807G>T	p.E1603X	GAG	TAG	B	B	**Observed**	Tyr		p.E1603Y	
c.4810A>T	p.K1604X	AAG	TAG	B	B	Predicted	Tyr		p.K1604Y	
c.4814C>A	p.S1605X	TCA	TAA	B	B	**Observed**	Tyr	Gln	p.S1605Y	p.S1605Q
c.4814C>G	p.S1605X	TCA	TGA	B	B	Predicted	Trp		p.S1605W	
c.4819G>T	p.E1607X	GAA	TAA	B	B	**Observed**	Tyr	Gln	p.E1607Y	p.E1607Q
c.4822A>T	p.K1608X	AAA	TAA	B	B	Predicted	Tyr	Gln	p.K1608Y	p.K1608Q
c.4834A>T	p.K1612X	AAG	TAG	B	B	Predicted	Tyr		p.K1612Y	
c.4837A>T	p.K1613X	AAA	TAA	B	B	Predicted	Tyr	Gln	p.K1613Y	p.K1613Q
c.4840A>T	p.K1614X	AAG	TAG	B	B	**Observed**	Tyr		p.K1614Y	
c.4853T>A	p.L1618X	TTG	TAG	B	B	Predicted	Tyr		p.L1618Y	
c.4869T>A	p.C1623X	TGT	TGA	B	B	Predicted	Trp		p.C1623W	
c.4870G>T	p.E1624X	GAA	TAA	B	B	Predicted	Tyr	Gln	p.E1624Y	p.E1624Q
c.4900G>T	p.E1634X	GAG	TAG	B	B	**Observed**	Tyr		p.E1634Y	
c.4903G>T	p.G1635X	GGA	TGA	B	B	Predicted	Trp		p.G1635W	
c.4912A>T	p.K1638X	AAG	TAG	B	B	Predicted	Tyr		p.K1638Y	
c.4918G>T	p.E1640X	GAA	TAA	B	B	**Observed**	Tyr	Gln	p.E1640Y	p.E1640Q
c.4924G>T	p.E1642X	GAA	TAA	B	B	Predicted	Tyr	Gln	p.E1642Y	p.E1642Q
c.4934G>A	p.W1645X	TGG	TAG	B	B	**Observed**	Tyr		p.W1645Y	
c.4939A>T	p.K1647X	AAG	TAG	B	B	Predicted	Tyr		p.K1647Y	
c.4954G>T	p.E1652X	GAA	TAA	B	B	Predicted	Tyr	Gln	p.E1652Y	p.E1652Q
c.4965C>A	p.C1655X	TGC	TGA	B	B	Predicted	Trp		p.C1655W	
c.4985T>A	p.L1662X	TTG	TAG	B	B	Predicted	Tyr		p.L1662Y	
c.4987A>T	p.K1663X	AAA	TAA	B	B	**Observed**	Tyr	Gln	p.K1663Y	p.K1663Q
c.5002G>T	p.E1668X	GAA	TAA	a3	LC	Predicted	Tyr	Gln	p.E1668Y	p.E1668Q
c.5023C>T	p.Q1675X	CAG	TAG	a3	LC	**Observed**	Tyr		p.Q1675Y	
c.5027C>A	p.S1676X	TCA	TAA	a3	LC	Predicted	Tyr	Gln	p.S1676Y	p.S1676Q
c.5027C>G	p.S1676X	TCA	TGA	a3	LC	Predicted	Trp		p.S1676W	
c.5035G>T	p.E1679X	GAG	TAG	a3	LC	Predicted	Tyr		p.E1679Y	
c.5038G>T	p.E1680X	GAA	TAA	a3	LC	**Observed**	Tyr	Gln	p.E1680Y	p.E1680Q
c.5063C>A	p.S1688X	TCA	TAA	a3	LC	Predicted	Tyr	Gln	p.S1688Y	p.S1688Q
c.5063C>G	p.S1688X	TCA	TGA	a3	LC	**Observed**	Trp		p.S1688W	
c.5068G>T	p.E1690X	GAA	TAA	a3	LC	Predicted	Tyr	Gln	p.E1690Y	p.E1690Q
c.5074A>T	p.K1692X	AAG	TAG	a3	LC	Predicted	Tyr		p.K1692Y	
c.5077A>T	p.K1693X	AAG	TAG	a3	LC	Predicted	Tyr		p.K1693Y	
c.5080G>T	p.E1694X	GAA	TAA	a3	LC	**Observed**	Tyr	Gln	p.E1694Y	p.E1694Q
c.5101G>T	p.E1701X	GAG	TAG	a3	LC	**Observed**	Tyr		p.E1701Y	
c.5107G>T	p.E1703X	GAA	TAA	a3	LC	Predicted	Tyr	Gln	p.E1703Y	p.E1703Q
c.5113C>T	p.Q1705X	CAG	TAG	a3	LC	**Observed**	Tyr		p.Q1705Y	
c.5134A>T	p.K1712X	AAG	TAG	A3	LC	Predicted	Tyr		p.K1712Y	
c.5137A>T	p.K1713X	AAA	TAA	A3	LC	Predicted	Tyr	Gln	p.K1713Y	p.K1713Q
c.5143C>T	p.R1715X	CGA	TGA	A3	LC	**Observed**	Trp		p.R1715W	
c.5167G>T	p.E1723X	GAG	TAG	A3	LC	**Observed**	Tyr		p.E1723Y	
c.5177G>A	p.W1726X	TGG	TAG	A3	LC	**Observed**	Tyr		p.W1726Y	
c.5212A>T	p.R1738X	AGA	TGA	A3	LC	Predicted	Trp		p.R1738W	
c.5224C>T	p.Q1742X	CAG	TAG	A3	LC	Predicted	Tyr		p.Q1742Y	
c.5242C>T	p.Q1748X	CAG	TAG	A3	LC	**Observed**	Tyr		p.Q1748Y	
c.5248A>T	p.K1750X	AAG	TAG	A3	LC	Predicted	Tyr		p.K1750Y	
c.5251A>T	p.K1751X	AAA	TAA	A3	LC	**Observed**	Tyr	Gln	p.K1751Y	p.K1751Q
c.5263C>T	p.Q1755X	CAG	TAG	A3	LC	**Observed**	Tyr		p.Q1755Y	
c.5266G>T	p.E1756X	GAA	TAA	A3	LC	Predicted	Tyr	Gln	p.E1756Y	p.E1756Q
c.5290C>T	p.Q1764X	CAG	TAG	A3	LC	Predicted	Tyr		p.Q1764Y	
c.5297T>A	p.L1766X	TTA	TAA	A3	LC	Predicted	Tyr	Gln	p.L1766Y	p.L1766Q
c.5297T>G	p.L1766X	TTA	TGA	A3	LC	Predicted	Trp		p.L1766W	
c.5305G>T	p.G1769X	GGA	TGA	A3	LC	Predicted	Trp		p.G1769W	
c.5308G>T	p.E1770X	GAA	TAA	A3	LC	Predicted	Tyr	Gln	p.E1770Y	p.E1770Q
c.5317G>T	p.E1773X	GAA	TAA	A3	LC	**Observed**	Tyr	Gln	p.E1773Y	p.E1773Q
c.5324T>A	p.L1775X	TTG	TAG	A3	LC	Predicted	Tyr		p.L1775Y	
c.5326G>T	p.G1776X	GGA	TGA	A3	LC	Predicted	Trp		p.G1776W	
c.5347A>T	p.R1783X	AGA	TGA	A3	LC	Predicted	Trp		p.R1783W	
c.5353G>T	p.E1785X	GAA	TAA	A3	LC	Predicted	Tyr	Gln	p.E1785Y	p.E1785Q
c.5359G>T	p.E1787X	GAA	TAA	A3	LC	Predicted	Tyr	Gln	p.E1787Y	p.E1787Q
c.5383A>T	p.R1795X	AGA	TGA	A3	LC	Predicted	Trp		p.R1795W	
c.5389C>T	p.Q1797X	CAG	TAG	A3	LC	**Observed**	Tyr		p.Q1797Y	
c.5434G>T	p.E1812X	GAG	TAG	A3	LC	**Observed**	Tyr		p.E1812Y	
c.5437G>T	p.E1813X	GAA	TAA	A3	LC	Predicted	Tyr	Gln	p.E1813Y	p.E1813Q
c.5443C>T	p.Q1815X	CAG	TAG	A3	LC	**Observed**	Tyr		p.Q1815Y	
c.5452G>T	p.G1818X	GGA	TGA	A3	LC	**Observed**	Trp		p.G1818W	
c.5458G>T	p.E1820X	GAA	TAA	A3	LC	Predicted	Tyr	Gln	p.E1820Y	p.E1820Q
c.5464A>T	p.R1822X	AGA	TGA	A3	LC	Predicted	Trp		p.R1822W	
c.5467A>T	p.K1823X	AAA	TAA	A3	LC	Predicted	Tyr	Gln	p.K1823Y	p.K1823Q
c.5479A>T	p.K1827X	AAG	TAG	A3	LC	Predicted	Tyr		p.K1827Y	
c.5488G>T	p.E1830X	GAA	TAA	A3	LC	Predicted	Tyr	Gln	p.E1830Y	p.E1830Q
c.5494A>T	p.K1832X	AAA	TAA	A3	LC	Predicted	Tyr	Gln	p.K1832Y	p.K1832Q
c.5507G>A	p.W1836X	TGG	TAG	A3	LC	**Observed**	Tyr		p.W1836Y	
c.5509A>T	p.K1837X	AAA	TAA	A3	LC	**Observed**	Tyr	Gln	p.K1837Y	p.K1837Q
c.5536A>T	p.K1846X	AAA	TAA	A3	LC	**Observed**	Tyr	Gln	p.K1846Y	p.K1846Q
c.5542G>T	p.E1848X	GAG	TAG	A3	LC	Predicted	Tyr		p.E1848Y	
c.5553C>A	p.C1851X	TGC	TGA	A3	LC	**Observed**	Trp		p.C1851W	
c.5554A>T	p.K1852X	AAA	TAA	A3	LC	Predicted	Tyr	Gln	p.K1852Y	p.K1852Q
c.5561G>A	p.W1854X	TGG	TAG	A3	LC	**Observed**	Tyr		p.W1854Y	
c.5587G>T	p.E1863X	GAA	TAA	A3	LC	Predicted	Tyr	Gln	p.E1863Y	p.E1863Q
c.5590A>T	p.K1864X	AAA	TAA	A3	LC	Predicted	Tyr	Gln	p.K1864Y	p.K1864Q
c.5603C>A	p.S1868X	TCA	TAA	A3	LC	Predicted	Tyr	Gln	p.S1868Y	p.S1868Q
c.5603C>G	p.S1868X	TCA	TGA	A3	LC	**Observed**	Trp		p.S1868W	
c.5614G>T	p.G1872X	GGA	TGA	A3	LC	Predicted	Trp		p.G1872W	
c.5631C>A	p.C1877X	TGC	TGA	A3	LC	Predicted	Trp		p.C1877W	
c.5662A>T	p.R1888X	AGA	TGA	A3	LC	**Observed**	Trp		p.R1888W	
c.5677C>T	p.Q1893X	CAG	TAG	A3	LC	**Observed**	Tyr		p.Q1893Y	
c.5680G>T	p.E1894X	GAA	TAA	A3	LC	Predicted	Tyr	Gln	p.E1894Y	p.E1894Q
c.5710G>T	p.E1904X	GAG	TAG	A3	LC	Predicted	Tyr		p.E1904Y	
c.5716A>T	p.K1906X	AAA	TAA	A3	LC	Predicted	Tyr	Gln	p.K1906Y	p.K1906Q
c.5723G>A	p.W1908X	TGG	TAG	A3	LC	**Observed**	Tyr		p.W1908Y	
c.5734G>T	p.E1912X	GAA	TAA	A3	LC	Predicted	Tyr	Gln	p.E1912Y	p.E1912Q
c.5743G>T	p.E1915X	GAA	TAA	A3	LC	**Observed**	Tyr	Gln	p.E1915Y	p.E1915Q
c.5746A>T	p.R1916X	AGA	TGA	A3	LC	Predicted	Trp		p.R1916W	
c.5754C>A	p.C1918X	TGC	TGA	A3	LC	Predicted	Trp		p.C1918W	
c.5766C>A	p.C1922X	TGC	TGA	A3	LC	**Observed**	Trp		p.C1922W	
c.5773C>T	p.Q1925X	CAG	TAG	A3	LC	Predicted	Tyr		p.Q1925Y	
c.5779G>T	p.E1927X	GAA	TAA	A3	LC	Predicted	Tyr	Gln	p.E1927Y	p.E1927Q
c.5794A>T	p.K1932X	AAA	TAA	A3	LC	Predicted	Tyr	Gln	p.K1932Y	p.K1932Q
c.5797G>T	p.E1933X	GAG	TAG	A3	LC	Predicted	Tyr		p.E1933Y	
c.5852T>A	p.L1951X	TTA	TAA	A3	LC	Predicted	Tyr	Gln	p.L1951Y	p.L1951Q
c.5852T>G	p.L1951X	TTA	TGA	A3	LC	Predicted	Trp		p.L1951W	
c.5863C>T	p.Q1955X	CAG	TAG	A3	LC	Predicted	Tyr		p.Q1955Y	
c.5878C>T	p.R1960X	CGA	TGA	A3	LC	**Observed**	Trp		p.R1960W	
c.5882G>A	p.W1961X	TGG	TAG	A3	LC	**Observed**	Tyr		p.W1961Y	
c.5908G>T	p.E1970X	GAA	TAA	A3	LC	Predicted	Tyr	Gln	p.E1970Y	p.E1970Q
c.5935G>T	p.G1979X	GGA	TGA	A3	LC	Predicted	Trp		p.G1979W	
c.5953C>T	p.R1985X	CGA	TGA	A3	LC	**Observed**	Trp		p.R1985W	
c.5956A>T	p.K1986X	AAA	TAA	A3	LC	Predicted	Tyr	Gln	p.K1986Y	p.K1986Q
c.5959A>T	p.K1987X	AAA	TAA	A3	LC	Predicted	Tyr	Gln	p.K1987Y	p.K1987Q
c.5962G>T	p.E1988X	GAG	TAG	A3	LC	Predicted	Tyr		p.E1988Y	
c.5965G>T	p.E1989X	GAG	TAG	A3	LC	Predicted	Tyr		p.E1989Y	
c.5971A>T	p.K1991X	AAA	TAA	A3	LC	Predicted	Tyr	Gln	p.K1991Y	p.K1991Q
c.6007G>T	p.E2003X	GAG	TAG	A3	LC	Predicted	Tyr		p.E2003Y	
c.6016G>T	p.E2006X	GAA	TAA	A3	LC	**Observed**	Tyr	Gln	p.E2006Y	p.E2006Q
c.6023T>G	p.L2008X	TTA	TGA	A3	LC	Predicted	Trp		p.L2008W	
c.6023T>A	p.L2008X	TTA	TAA	A3	LC	Predicted	Tyr	Gln	p.L2008Y	p.L2008Q
c.6031A>T	p.K2011X	AAA	TAA	A3	LC	Predicted	Tyr	Gln	p.K2011Y	p.K2011Q
c.6037G>T	p.G2013X	GGA	TGA	A3	LC	Predicted	Trp		p.G2013W	
c.6044G>A	p.W2015X	TGG	TAG	A3	LC	**Observed**	Tyr		p.W2015Y	
c.6052G>T	p.E2018X	GAA	TAA	A3	LC	**Observed**	Tyr	Gln	p.E2018Y	p.E2018Q
c.6057C>A	p.C2019X	TGC	TGA	A3	LC	**Observed**	Trp		p.C2019W	
c.6067G>T	p.E2023X	GAG	TAG	A3	LC	Predicted	Tyr		p.E2023Y	
c.6115A>T	p.K2039X	AAG	TAG	C1	LC	**Observed**	Tyr		p.K2039Y	
c.6120T>A	p.C2040X	TGT	TGA	C1	LC	**Observed**	Trp		p.C2040W	
c.6121C>T	p.Q2041X	CAG	TAG	C1	LC	Predicted	Tyr		p.Q2041Y	
c.6133G>T	p.G2045X	GGA	TGA	C1	LC	**Observed**	Trp		p.G2045W	
c.6145G>T	p.G2049X	GGA	TGA	C1	LC	Predicted	Trp		p.G2049W	
c.6154A>T	p.R2052X	AGA	TGA	C1	LC	Predicted	Trp		p.R2052W	
c.6163C>T	p.Q2055X	CAG	TAG	C1	LC	**Observed**	Tyr		p.Q2055Y	
c.6176C>A	p.S2059X	TCA	TAA	C1	LC	Predicted	Tyr	Gln	p.S2059Y	p.S2059Q
c.6176C>G	p.S2059X	TCA	TGA	C1	LC	Predicted	Trp		p.S2059W	
c.6178G>T	p.G2060X	GGA	TGA	C1	LC	Predicted	Trp		p.G2060W	
c.6187G>T	p.G2063X	GGA	TGA	C1	LC	**Observed**	Trp		p.G2063W	
c.6190C>T	p.Q2064X	CAG	TAG	C1	LC	Predicted	Tyr		p.Q2064Y	
c.6194G>A	p.W2065X	TGG	TAG	C1	LC	**Observed**	Tyr		p.W2065Y	
c.6202A>T	p.K2068X	AAG	TAG	C1	LC	Predicted	Tyr		p.K2068Y	
c.6211A>T	p.R2071X	AGA	TGA	C1	LC	Predicted	Trp		p.R2071W	
c.6226G>T	p.G2076X	GGA	TGA	C1	LC	**Observed**	Trp		p.G2076W	
c.6230C>A	p.S2077X	TCA	TAA	C1	LC	Predicted	Tyr	Gln	p.S2077Y	p.S2077Q
c.6230C>G	p.S2077X	TCA	TGA	C1	LC	**Observed**	Trp		p.S2077W	
c.6242G>A	p.W2081X	TGG	TAG	C1	LC	Predicted	Tyr		p.W2081Y	
c.6250A>T	p.K2084X	AAG	TAG	C1	LC	**Observed**	Tyr		p.K2084Y	
c.6253G>T	p.E2085X	GAG	TAG	C1	LC	**Observed**	Tyr		p.E2085Y	
c.6266G>A	p.W2089X	TGG	TAG	C1	LC	**Observed**	Tyr		p.W2089Y	
c.6271A>T	p.K2091X	AAG	TAG	C1	LC	Predicted	Tyr		p.K2091Y	
c.6284T>A	p.L2095X	TTG	TAG	C1	LC	Predicted	Tyr		p.L2095Y	
c.6310A>T	p.K2104X	AAG	TAG	C1	LC	**Observed**	Tyr		p.K2104Y	
c.6316C>T	p.Q2106X	CAG	TAG	C1	LC	**Observed**	Tyr		p.Q2106Y	
c.6328C>T	p.Q2110X	CAG	TAG	C1	LC	Predicted	Tyr		p.Q2110Y	
c.6331A>T	p.K2111X	AAG	TAG	C1	LC	Predicted	Tyr		p.K2111Y	
c.6355C>T	p.Q2119X	CAG	TAG	C1	LC	**Observed**	Tyr		p.Q2119Y	
c.6385A>T	p.K2129X	AAG	TAG	C1	LC	**Observed**	Tyr		p.K2129Y	
c.6388A>T	p.K2130X	AAG	TAG	C1	LC	Predicted	Tyr		p.K2130Y	
c.6392G>A	p.W2131X	TGG	TAG	C1	LC	Predicted	Tyr		p.W2131Y	
c.6394C>T	p.Q2132X	CAG	TAG	C1	LC	**Observed**	Tyr		p.Q2132Y	
c.6403C>T	p.R2135X	CGA	TGA	C1	LC	**Observed**	Trp		p.R2135W	
c.6406G>T	p.G2136X	GGA	TGA	C1	LC	Predicted	Trp		p.G2136W	
c.6418G>T	p.G2140X	GGA	TGA	C1	LC	**Observed**	Trp		p.G2140W	
c.6425T>G	p.L2142X	TTA	TGA	C1	LC	Predicted	Trp		p.L2142W	
c.6425T>A	p.L2142X	TTA	TAA	C1	LC	**Observed**	Tyr	Gln	p.L2142Y	p.L2142Q
c.6452C>A	p.S2151X	TCA	TAA	C1	LC	Predicted	Tyr	Gln	p.S2151Y	p.S2151Q
c.6452C>G	p.S2151X	TCA	TGA	C1	LC	Predicted	Trp		p.S2151W	
c.6463A>T	p.K2155X	AAA	TAA	C1	LC	Predicted	Tyr	Gln	p.K2155Y	p.K2155Q
c.6496C>T	p.R2166X	CGA	TGA	C1	LC	**Observed**	Trp		p.R2166W	
c.6509T>A	p.L2170X	TTG	TAG	C1	LC	Predicted	Tyr		p.L2170Y	
c.6550G>T	p.E2184X	GAG	TAG	C1	LC	**Observed**	Tyr		p.E2184Y	
c.6554T>A	p.L2185X	TTG	TAG	C1	LC	Predicted	Tyr		p.L2185Y	
c.6564T>A	p.C2188X	TGT	TGA	C1	LC	Predicted	Trp		p.C2188W	
c.6569T>G	p.L2190X	TTA	TGA	C1	LC	Predicted	Trp		p.L2190W	
c.6569T>A	p.L2190X	TTA	TAA	C1	LC	Predicted	Tyr	Gln	p.L2190Y	p.L2190Q
c.6579C>A	p.C2193X	TGC	TGA	C1	LC	Predicted	Trp		p.C2193W	
c.6590T>A	p.L2197X	TTG	TAG	C1	LC	**Observed**	Tyr		p.L2197Y	
c.6592G>T	p.G2198X	GGA	TGA	C2	LC	Predicted	Trp		p.G2198W	
c.6598G>T	p.E2200X	GAG	TAG	C2	LC	Predicted	Tyr		p.E2200Y	
c.6604A>T	p.K2202X	AAA	TAA	C2	LC	Predicted	Tyr	Gln	p.K2202Y	p.K2202Q
c.6614C>A	p.S2205X	TCA	TAA	C2	LC	Predicted	Tyr	Gln	p.S2205Y	p.S2205Q
c.6614C>G	p.S2205X	TCA	TGA	C2	LC	**Observed**	Trp		p.S2205W	
c.6622C>T	p.Q2208X	CAG	TAG	C2	LC	**Observed**	Tyr		p.Q2208Y	
c.6635C>A	p.S2212X	TCA	TAA	C2	LC	Predicted	Tyr	Gln	p.S2212Y	p.S2212Q
c.6635C>G	p.S2212X	TCA	TGA	C2	LC	Predicted	Trp		p.S2212W	
c.6665G>A	p.W2222X	TGG	TAG	C2	LC	**Observed**	Tyr		p.W2222Y	
c.6674C>A	p.S2225X	TCA	TAA	C2	LC	Predicted	Tyr	Gln	p.S2225Y	p.S2225Q
c.6674C>G	p.S2225X	TCA	TGA	C2	LC	Predicted	Trp		p.S2225W	
c.6676A>T	p.K2226X	AAA	TAA	C2	LC	Predicted	Tyr	Gln	p.K2226Y	p.K2226Q
c.6682C>T	p.R2228X	CGA	TGA	C2	LC	**Observed**	Trp		p.R2228W	
c.6713G>A	p.W2238X	TGG	TAG	C2	LC	**Observed**	Tyr		p.W2238Y	
c.6715A>T	p.R2239X	AGA	TGA	C2	LC	Predicted	Trp		p.R2239W	
c.6721C>T	p.Q2241X	CAG	TAG	C2	LC	**Observed**	Tyr		p.Q2241Y	
c.6736A>T	p.K2246X	AAA	TAA	C2	LC	**Observed**	Tyr	Gln	p.K2246Y	p.K2246Q
c.6739G>T	p.E2247X	GAG	TAG	C2	LC	**Observed**	Tyr		p.E2247Y	
c.6743G>A	p.W2248X	TGG	TAG	C2	LC	**Observed**	Tyr		p.W2248Y	
c.6760C>T	p.Q2254X	CAG	TAG	C2	LC	**Observed**	Tyr		p.Q2254Y	
c.6763A>T	p.K2255X	AAG	TAG	C2	LC	Predicted	Tyr		p.K2255Y	
c.6772A>T	p.K2258X	AAA	TAA	C2	LC	**Observed**	Tyr	Gln	p.K2258Y	p.K2258Q
c.6781G>T	p.G2261X	GGA	TGA	C2	LC	Predicted	Trp		p.G2261W	
c.6793C>T	p.Q2265X	CAG	TAG	C2	LC	Predicted	Tyr		p.Q2265Y	
c.6796G>T	p.G2266X	GGA	TGA	C2	LC	Predicted	Trp		p.G2266W	
c.6802A>T	p.K2268X	AAA	TAA	C2	LC	Predicted	Tyr	Gln	p.K2268Y	p.K2268Q
c.6829A>T	p.K2277X	AAG	TAG	C2	LC	Predicted	Tyr		p.K2277Y	
c.6832G>T	p.E2278X	GAG	TAG	C2	LC	Predicted	Tyr		p.E2278Y	
c.6865C>T	p.Q2289X	CAG	TAG	C2	LC	**Observed**	Tyr		p.Q2289Y	
c.6869G>A	p.W2290X	TGG	TAG	C2	LC	Predicted	Tyr		p.W2290Y	
c.6883C>T	p.Q2295X	CAG	TAG	C2	LC	Predicted	Tyr		p.Q2295Y	
c.6892A>T	p.K2298X	AAA	TAA	C2	LC	Predicted	Tyr	Gln	p.K2298Y	p.K2298Q
c.6898A>T	p.K2300X	AAG	TAG	C2	LC	Predicted	Tyr		p.K2300Y	
c.6907C>T	p.Q2303X	CAG	TAG	C2	LC	**Observed**	Tyr		p.Q2303Y	
c.6910G>T	p.G2304X	GGA	TGA	C2	LC	Predicted	Trp		p.G2304W	
c.6959T>A	p.L2320X	TTA	TAA	C2	LC	Predicted	Tyr	Gln	p.L2320Y	p.L2320Q
c.6959T>G	p.L2320X	TTA	TGA	C2	LC	Predicted	Trp		p.L2320W	
c.6976C>T	p.R2326X	CGA	TGA	C2	LC	**Observed**	Trp		p.R2326W	
c.6988C>T	p.Q2330X	CAG	TAG	C2	LC	**Observed**	Tyr		p.Q2330Y	
c.6995G>A	p.W2332X	TGG	TAG	C2	LC	**Observed**	Tyr		p.W2332Y	
c.7003C>T	p.Q2335X	CAG	TAG	C2	LC	**Observed**	Tyr		p.Q2335Y	
c.7021G>T	p.E2341X	GAG	TAG	C2	LC	**Observed**	Tyr		p.E2341Y	
c.7035C>A	p.C2345X	TGC	TGA	C2	LC	**Observed**	Trp		p.C2345W	
c.7036G>T	p.E2346X	GAG	TAG	C2	LC	Predicted	Tyr		p.E2346Y	
c.7042C>T	p.Q2348X	CAG	TAG	C2	LC	Predicted	Tyr		p.Q2348Y	
