# Supplementary material for: Immunogenic implications of translational readthrough modulate the association of F8 nonsense mutations with inhibitors in Hemophilia A
Source: Mol Med. 2026 Jun 27;32:144. doi: 10.1186/s10020-026-01536-y (PMC13576119; doi:10.1186/s10020-026-01536-y)
Supplement: Supplementary file 4 [file 10020_2026_1536_MOESM4_ESM.pdf]

Protein variant	Missense Change	DRB1 0101	DRB1 0301	DRB1 0401	DRB1 0405	DRB1 0701	DRB1 0801	DRB1 0901	DRB1 1101	DRB1 1201	DRB1 1301	DRB1 1501	DRB3 0101	DRB3 0202	DRB4 0101	DRB5 0101	HLA-DPA10103-DPB10201	HLA-DPA10103-DPB10401	HLA-DPA10201-DPB10101	HLA-DPA10201-DPB10501	HLA-DPA10301-DPB10402	HLA-DQA10101-DQB10501	HLA-DQA10102-DQB10602	HLA-DQA10301-DQB10302	HLA-DQA10401-DQB10402	HLA-DQA10501-DQB10201	HLA-DQA10501-DQB10301	min	max	mean	median
p.R15X	p.R15W	14,01	18,65	18,59	14,23	9,53	18,88	7,87	8,38	16,92	10,06	10,91	11,69	19,53	5,62	-4,97	14,63	24,31	15,50	-1,27	28,24	0,47	2,59	-0,05	0,57	0,38	-2,92	-4,97	28,24	10,09	10,68
p.C17X	p.C17W	-26,40	-13,05	-21,99	-25,65	-9,67	-15,03	-9,26	-5,67	-30,93	-7,88	-31,05	-11,60	-16,16	-21,50	-13,94	-14,69	-29,70	0,00	-0,02	-18,70	0,00	-16,78	0,00	-16,82	-25,80	-52,91	-52,91	0,00	-16,74	-15,60
p.R22X	p.R22W	6,50	22,74	4,60	-5,83	-1,59	11,02	6,10	17,71	8,64	18,72	-30,93	5,57	8,03	15,81	6,74	17,68	3,63	19,29	9,60	15,63	-9,79	6,94	8,33	-5,18	4,75	1,31	-30,93	22,74	6,39	6,84
p.R23X	p.R23W	-9,30	34,64	4,59	-4,90	-10,64	4,08	2,95	6,42	-44,27	20,50	-31,60	-3,27	1,40	15,87	-9,38	-11,57	-10,49	-0,24	6,68	-5,26	-8,47	13,58	-5,07	1,32	-0,80	1,25	-44,27	34,64	-1,61	-0,52
p.E30X	p.E30Y	1,78	4,00	0,87	1,48	4,61	5,60	2,88	4,59	-14,94	-10,21	-0,99	9,50	3,85	1,89	-2,12	-5,98	-1,37	1,85	5,14	1,03	3,98	1,36	1,87	-12,73	4,29	1,63	-14,94	9,50	0,53	1,82
p.E30X	p.E30Q	0,11	0,32	0,05	0,12	0,57	-2,65	0,08	0,29	-4,78	-8,78	0,53	1,50	-1,84	0,13	-2,63	0,35	0,12	0,21	-1,18	0,34	0,69	-13,62	0,57	-8,69	1,00	-0,83	-13,62	1,50	-1,46	0,12
p.S32X	p.S32W	2,32	9,12	1,16	1,90	9,41	8,55	3,72	8,65	11,37	13,93	-20,82	1,37	19,04	2,39	7,18	-0,63	-0,31	-0,61	-0,94	0,15	-9,49	-8,83	10,34	5,72	4,18	25,70	-20,82	25,70	4,02	3,06
p.S32X	p.S32Y	1,23	-1,92	0,57	0,97	5,24	4,68	0,39	4,66	-14,07	0,23	-27,59	-6,37	12,82	1,22	3,90	-2,55	-0,85	-1,22	-1,50	-2,12	-8,91	-4,90	5,84	0,76	2,20	17,20	-27,59	17,20	-0,39	0,48
p.S32X	p.S32Q	-0,23	-2,95	-0,12	-0,19	-0,99	-1,09	-0,35	-1,21	-4,55	0,28	-2,27	-0,46	5,51	-0,24	-0,85	-2,50	-0,89	-1,35	-0,97	-2,05	-0,71	-21,73	-0,87	-1,88	-0,46	7,79	-21,73	7,79	-1,36	-0,88
p.W33X	p.W33Y	-1,22	-4,42	-0,59	-0,85	-4,72	-6,18	-3,25	-6,78	-29,79	-16,20	-12,33	-4,39	-1,30	-0,17	-4,90	0,20	0,06	-0,38	-3,00	-1,50	-2,12	-6,59	-4,27	-5,39	-1,90	-0,39	-29,79	0,20	-4,71	-3,13
p.E42X	p.E42Y	0,96	2,24	0,66	0,95	-12,00	-5,68	0,89	-3,76	-11,17	-17,47	4,90	-0,34	-3,66	-1,78	-8,86	2,17	0,76	1,29	1,91	2,18	0,68	-5,25	4,29	-2,65	2,91	5,71	-17,47	5,71	-1,55	0,72
p.R48X	p.R48W	2,98	20,28	-8,24	-1,22	9,21	12,33	0,08	-3,74	0,04	-12,53	-18,09	0,01	-23,12	9,49	13,70	0,00	-15,73	0,50	28,51	-14,65	-15,52	17,51	4,22	-8,16	-14,66	-20,07	-23,12	28,51	-1,62	0,01
p.R52X	p.R52W	28,03	4,02	0,10	0,69	0,52	-8,48	0,01	-23,68	3,21	0,02	10,01	0,00	0,00	0,00	0,19	-8,74	0,51	-11,13	0,00	0,35	-1,44	15,45	1,92	3,50	-0,29	5,44	-23,68	28,03	0,79	0,14
p.K55X	p.K55Y	5,73	0,01	5,12	5,46	3,66	6,99	0,77	0,04	3,01	3,83	5,43	4,91	1,50	10,64	0,00	4,50	3,33	1,59	0,58	3,00	8,06	0,11	5,34	5,95	3,15	0,01	0,00	10,64	3,56	3,40
p.K55X	p.K55Q	2,49	0,01	1,39	1,76	0,93	2,97	0,26	0,02	2,88	1,28	2,54	1,00	0,39	3,74	0,00	1,57	0,95	-6,57	0,26	0,83	1,65	0,02	1,60	3,33	0,88	0,00	-6,57	3,74	1,01	0,97
p.S62X	p.S62Y	5,86	3,79	14,12	1,56	2,81	0,95	16,73	-3,29	1,32	-2,51	1,45	0,41	11,00	10,53	4,23	0,92	0,11	-1,16	-5,97	0,61	-1,92	8,30	0,32	-3,91	6,58	27,88	-5,97	27,88	3,87	1,38
p.S62X	p.S62W	10,71	9,10	14,63	8,72	4,88	1,84	22,56	-0,04	3,76	-1,86	2,54	2,85	19,75	17,58	5,11	1,53	0,17	1,54	-5,15	1,10	5,54	10,66	0,65	-4,68	10,64	34,37	-5,15	34,37	6,85	4,32
p.S62X	p.S62Q	-1,31	-1,52	3,90	-1,89	-0,47	-0,17	10,94	-0,68	-0,72	-0,37	-0,27	0,69	4,59	-7,62	-0,96	0,04	-0,02	-0,29	-2,23	-0,07	-7,17	-7,55	-2,77	-0,22	-1,37	25,63	-7,62	25,63	0,31	-0,42
p.K66X	p.K66Y	-0,65	7,56	3,29	0,05	0,00	19,01	0,01	13,43	0,84	1,89	-2,73	0,15	1,27	1,82	3,09	1,76	0,22	2,95	3,55	0,68	0,00	6,76	-7,84	0,01	1,00	7,07	-7,84	19,01	2,51	1,13
p.K66X	p.K66Q	-10,02	-5,31	-6,20	0,03	0,00	10,59	0,01	7,33	-4,93	1,89	-0,93	0,06	0,42	0,36	1,86	1,04	0,00	-0,28	-1,04	-0,02	0,00	-11,86	-22,14	0,00	0,22	1,46	-22,14	10,59	-1,64	0,00
p.K67X	p.K67Y	5,46	7,89	7,65	3,91	-0,45	7,99	-5,88	17,63	7,81	3,60	1,65	1,31	1,29	1,82	9,18	-0,54	-0,12	-4,36	-4,09	-0,68	0,46	2,22	-8,18	4,25	5,34	-6,76	-8,18	17,63	2,24	1,74
p.E72X	p.E72Y	-4,39	0,45	-2,82	-3,13	-2,32	1,07	-1,86	0,00	-0,27	-1,38	-1,26	-4,18	0,00	11,76	-2,19	0,02	0,57	0,00	0,91	0,18	-0,25	7,68	6,91	-1,27	-2,91	0,71	-4,39	11,76	0,08	-0,13
p.E72X	p.E72Q	-3,57	1,57	-0,94	-2,42	-1,85	0,08	-1,70	0,00	-0,85	-0,93	-0,73	-0,87	0,00	-1,05	-1,41	0,00	0,10	1,07	0,13	0,02	-0,14	1,83	2,11	-0,97	-0,29	0,31	-3,57	2,11	-0,40	-0,22
p.K82X	p.K82Y	0,00	12,77	6,19	0,03	2,98	10,65	1,21	12,86	-9,17	0,13	0,00	0,15	-1,61	-0,99	2,32	0,03	0,32	3,05	16,56	0,24	0,00	2,30	-0,05	5,68	1,44	2,70	-9,17	16,56	2,68	0,77
p.W87X	p.W87Y	-0,25	-1,98	-8,26	-10,28	-4,26	-1,01	-0,94	-0,90	-2,93	-2,85	-9,95	-9,45	-1,07	-2,15	-0,84	0,00	-0,01	-1,67	-0,01	-6,09	-5,00	-3,12	-2,39	-1,60	-1,67	-1,54	-10,28	0,00	-3,09	-1,83
p.Q96X	p.Q96Y	0,19	3,46	-6,95	-6,85	3,93	7,60	10,25	5,20	-11,94	5,97	7,03	-1,84	4,54	1,12	0,00	-0,35	0,13	6,37	0,10	0,05	7,61	1,49	1,67	-17,51	0,03	0,99	-17,51	10,25	0,86	1,06
p.E98X	p.E98Y	0,21	-6,66	-22,48	-15,78	2,14	-6,37	1,75	0,00	0,00	-16,76	-7,39	-20,67	-19,43	4,44	4,07	3,22	1,56	2,97	1,19	0,36	-6,68	12,03	9,72	-24,55	-8,17	-8,43	-24,55	12,03	-4,60	0,00
p.K108X	p.K108Y	0,42	25,31	3,95	5,99	1,20	-1,03	-2,87	-0,25	1,17	1,24	0,34	-2,70	-8,54	8,11	1,73	-5,11	-6,73	4,13	14,91	-2,63	3,56	0,08	14,69	1,82	-0,25	-3,41	-8,54	25,31	2,11	0,79
p.W125X	p.W125Y	-2,90	-4,39	-3,07	-2,54	-0,83	0,19	-0,05	-6,04	-7,73	2,53	-0,97	-0,60	-2,06	1,77	-2,37	-1,14	0,27	-0,09	-1,58	-5,12	0,44	-0,31	-0,66	0,01	2,61	-0,55	-7,73	2,61	-1,35	-0,74
p.K126X	p.K126Y	-0,42	0,00	-4,42	-3,77	0,95	3,11	0,07	-1,74	5,54	10,49	2,47	0,13	0,53	7,61	0,81	16,46	0,46	15,00	39,49	-3,03	0,00	12,49	-3,30	0,15	-2,60	-1,91	-4,42	39,49	3,64	0,31
p.K126X	p.K126Q	1,53	0,00	3,13	0,32	1,03	7,26	0,12	4,07	2,51	6,99	0,97	0,20	2,31	2,14	3,20	-0,72	0,62	17,34	34,06	-5,90	0,00	2,38	-3,89	0,07	-0,94	0,77	-5,90	34,06	3,06	1,00
p.E129X	p.E129Y	1,18	2,25	-1,75	0,00	0,35	1,89	0,14	5,53	-0,38	-0,28	-0,02	1,57	1,89	1,57	0,08	1,29	-3,06	4,50	7,55	3,54	0,30	4,02	0,50	0,02	-0,51	1,11	-3,06	7,55	1,28	0,81
p.G130X	p.G130W	13,10	3,93	2,02	0,43	2,53	5,20	0,66	1,13	-2,94	6,89	4,20	9,85	15,53	14,77	3,51	-0,47	-6,06	1,06	1,87	-13,52	-0,33	26,88	2,62	0,89	2,68	7,21	-13,52	26,88	3,99	2,58
p.E132X	p.E132Y	-0,12	-6,77	-1,60	0,21	0,32	2,76	0,00	-3,47	-0,40	-2,78	0,32	-1,12	-1,27	1,90	-2,53	0,32	2,85	1,23	8,24	3,48	-0,54	2,52	1,97	0,01	-1,65	-0,02	-6,77	8,24	0,15	0,01
p.E132X	p.E132Q	-0,24	-1,18	0,00	0,01	-0,01	-6,06	0,03	-10,96	-2,43	-0,24	0,02	-0,23	-0,81	0,01	-1,08	-0,01	0,54	0,07	-0,16	0,12	-0,13	0,28	0,61	0,00	0,57	-0,31	-10,96	0,61	-0,83	-0,01
p.Q136X	p.Q136Y	1,30	13,13	7,48	1,69	0,45	-12,51	0,07	-8,12	2,85	-3,31	-26,78	0,38	1,04	15,99	1,02	-17,16	2,04	0,15	1,89	-1,14	0,12	2,83	2,57	-8,11	0,59	1,23	-26,78	15,99	-0,78	0,81
p.E141X	p.E141Y	-6,39	-3,64	1,35	7,39	0,47	-15,30	-3,37	-17,91	-6,17	-14,58	-1,89	-12,52	-4,70	3,50	-4,42	-29,00	0,73	-3,02	2,22	-11,53	0,22	6,65	19,92	-0,28	0,82	1,25	-29,00	19,92	-3,46	-2,45
p.K142X	p.K142Y	-4,28	-3,39	-2,70	0,23	2,75	-17,85	3,15	-16,31	0,59	-13,64	-12,99	-13,70	-10,52	-3,50	-0,36	3,55	-1,92	16,75	10,84	-26,49	-1,50	-14,18	1,11	-3,96	-2,32	-8,24	-26,49	16,75	-4,57	-3,04

p.K1086X	p.K1086Y	-1,54	-1,23	-5,96	-13,29	-9,17	-8,48	-34,02	-14,78	-0,55	-12,62	-3,72	-32,78	1,09	4,29	1,09	2,83	-19,26
p.K1086X	p.K1086Q	-3,18	5,97	-12,19	-18,28	0,84	-2,41	-9,44	1,65	0,82	-1,35	-1,78	-6,37	-1,83	-6,76	-8,37	0,73	-1,26
p.S1089X	p.S1089Y	1,19	0,91	-0,56	0,00	-2,62	0,00	0,00	-4,80	0,00	-11,06	0,79	-24,28	-2,51	2,98	-4,01	-1,95	-4,67
p.S1089X	p.S1089W	3,10	2,55	5,43	0,00	0,00	0,00	0,00	0,00	0,00	-4,90	2,49	-20,35	-1,01	7,15	1,14	-1,36	-8,41
p.S1089X	p.S1089Q	-0,41	-0,52	-1,26	0,00	0,00	0,00	0,00	0,00	0,00	-1,07	-0,31	-2,46	-0,26	-1,68	-1,23	-1,56	-0,01
p.S1090X	p.S1090Y	-0,10	0,00	-13,82	-13,63	3,80	-12,35	2,57	-9,53	2,61	-8,55	-2,57	-22,63	-3,24	-13,64	-12,57	-2,16	-16,01
p.S1090X	p.S1090W	3,10	0,01	-11,18	-11,29	7,36	-9,15	4,98	-6,75	5,15	1,27	2,49	-26,31	0,05	-15,22	-7,83	-1,96	-14,55
p.S1090X	p.S1090Q	-0,41	-0,01	-1,07	-0,81	-0,76	-0,92	-0,41	-0,58	-0,56	-0,90	-0,31	-1,90	0,00	-0,50	-1,22	6,89	-1,09
p.K1091X	p.K1091Y	0,84	-11,36	-12,60	-4,24	6,93	-3,28	4,27	-2,57	5,24	-11,20	2,14	-38,25	-7,55	-3,22	-18,40	21,97	-5,41
p.K1091X	p.K1091Q	1,09	1,95	1,85	2,00	2,05	-2,09	1,30	0,21	1,42	0,28	0,82	3,14	1,92	0,19	0,23	24,00	5,45
p.E1094X	p.E1094Y	-28,81	-0,42	-30,58	-1,36	-1,85	-7,93	-2,12	-8,94	-0,57	-14,78	3,47	-4,68	-19,86	1,98	-11,54	8,46	-2,70
p.E1094X	p.E1094Q	0,86	0,26	0,08	3,64	2,01	0,18	0,28	0,06	0,88	0,24	0,17	1,46	0,00	0,35	-1,71	-0,94	4,67
p.Q1098X	p.Q1098Y	-46,11	-0,50	-58,93	-4,43	-2,10	-4,85	-1,72	2,06	0,05	-10,71	-6,20	-21,40	-34,90	9,94	-13,06	-1,93	-25,13
p.K1099X	p.K1099Y	-23,51	0,58	-49,16	-14,34	-1,47	12,95	-2,41	4,45	-2,78	1,11	-10,57	-4,91	-37,74	1,50	7,87	2,28	-1,09
p.K1099X	p.K1099Q	-37,33	-3,53	-10,40	-8,42	-3,40	11,06	-3,32	6,38	-2,87	6,94	-2,66	0,00	-3,42	-5,03	4,30	0,80	-7,92
p.K1100X	p.K1100Y	-30,81	0,00	-24,82	3,36	9,10	7,60	3,23	12,72	0,84	19,23	4,91	0,00	-25,66	1,13	12,95	0,00	-18,48
p.K1100X	p.K1100Q	-2,75	0,00	0,11	-2,11	3,05	5,34	1,61	7,55	1,63	13,48	5,59	0,00	1,62	-5,94	9,31	0,00	0,80
p.E1101X	p.E1101Y	-41,69	-5,22	-46,68	-14,18	-6,48	0,76	-3,20	-2,24	-8,39	-6,65	8,37	-2,15	-35,25	-5,46	-11,56	-43,70	-6,95
p.S1114X	p.S1114Y	1,32	0,79	4,29	3,92	0,00	5,81	0,07	4,25	6,03	0,60	3,94	1,78	6,78	11,14	0,07	2,02	0,63
p.K1117X	p.K1117Y	0,66	-1,85	8,14	0,22	2,26	5,86	5,85	8,81	11,85	7,18	9,76	3,20	3,27	1,81	1,39	-2,86	-1,03
p.L1121X	p.L1121Y	0,16	-0,94	1,29	9,19	-0,03	0,00	-2,15	0,00	-15,87	0,69	-6,69	-0,35	-0,35	7,23	0,42	4,07	4,93
p.E1123X	p.E1123Y	7,15	6,41	4,08	9,18	-0,01	0,07	-1,34	1,46	-7,69	-5,86	-11,89	2,96	17,42	10,08	5,53	-0,14	0,33
p.E1123X	p.E1123Q	0,31	0,26	0,10	2,90	1,24	0,01	0,01	0,08	-2,11	-3,92	0,29	7,32	0,84	-6,10	0,49	1,35	2,63
p.S1124X	p.S1124Y	0,32	4,41	5,52	6,13	1,27	1,54	2,12	1,27	-12,06	-6,16	2,97	1,49	3,45	9,69	0,88	1,28	0,42
p.S1124X	p.S1124W	2,80	8,89	15,83	10,56	7,22	3,08	12,14	2,85	6,05	-1,44	6,35	2,81	9,48	13,91	4,66	2,71	0,79
p.S1124X	p.S1124Q	-0,14	-6,65	1,57	-2,44	-0,56	-0,30	-2,09	-0,35	-5,38	-3,37	-1,05	-0,64	-0,39	1,86	-0,28	-0,33	-0,13
p.W1127X	p.W1127Y	-0,47	-11,81	-4,08	-2,17	-1,40	-2,86	-3,36	-3,48	-4,78	-3,50	-4,07	-2,39	-1,67	-8,46	-0,99	-2,27	0,00
p.G1133X	p.G1133W	0,00	3,76	-43,65	-65,26	-4,76	3,49	-32,93	7,91	-26,68	-1,50	8,51	-22,17	-13,65	-7,83	0,00	-37,77	-31,03
p.K1134X	p.K1134Y	-33,26	6,21	-64,09	-67,47	-8,40	-3,18	-25,31	-4,15	-19,91	3,90	-25,20	-47,35	-10,05	-7,47	-28,02	-20,74	-66,64
p.K1146X	p.K1146Y	0,55	6,38	1,98	-3,42	-18,29	2,75	-2,84	2,53	2,66	10,18	5,19	-2,45	5,35	0,83	2,05	8,63	-2,84
p.L1148X	p.L1148Y	-0,60	16,73	-2,84	-4,56	-22,18	-2,72	-4,46	-3,73	6,12	-10,11	3,32	-18,77	-5,84	2,51	-2,65	-3,76	-4,47
p.L1148X	p.L1148W	-0,54	22,60	-2,10	-3,53	-18,10	-2,05	-1,40	-2,76	10,64	-6,18	9,06	-15,66	-4,38	4,28	-1,98	0,78	-2,31
p.L1148X	p.L1148Q	9,11	10,77	-1,04	10,25	5,98	22,20	-2,77	28,43	19,39	12,00	3,44	5,76	15,72	15,64	20,46	22,39	39,17
p.L1151X	p.L1151Y	2,31	4,16	-2,83	-0,74	-10,63	5,47	-3,67	6,18	16,00	-5,91	-7,87	-13,33	-3,08	1,54	4,89	-4,31	0,00
p.L1151X	p.L1151W	5,24	2,68	-1,58	3,96	-3,98	3,83	2,43	10,54	12,75	-8,33	-5,88	-6,76	1,92	0,95	5,87	-6,23	0,00
p.L1151X	p.L1151Q	3,39	-0,32	0,37	7,05	-7,39	7,43	3,86	12,14	21,89	0,99	9,32	2,47	-0,92	-0,97	2,52	1,28	0,00
p.G1152X	p.G1152W	1,31	5,07	-0,77	5,58	-4,79	3,52	13,47	3,74	0,15	-6,37	-7,70	-3,73	4,74	0,59	4,15	0,00	0,00
p.E1154X	p.E1154Y	0,29	14,90	4,26	3,83	-27,70	1,75	-7,24	1,31	-1,18	3,03	-8,28	-22,29	5,79	5,15	-0,63	0,20	0,05
p.E1154X	p.E1154Q	-0,12	2,24	0,54	-0,06	-7,67	-0,91	-0,77	-0,39	-0,37	-5,58	-1,77	7,81	-0,67	0,88	-0,78	0,02	0,01
p.K1155X	p.K1155Y	0,64	5,74	2,83	2,59	-19,25	3,21	-3,64	4,20	2,00	-1,42	0,79	-24,20	-0,93	0,83	3,75	0,29	0,07
p.K1155X	p.K1155Q	0,33	2,01	0,54	0,17	6,80	0,77	-1,32	1,50	0,83	5,08	1,75	-0,65	1,23	0,09	2,17	0,08	0,02
p.E1158X	p.E1158Y	0,00	2,32	0,00	1,05	0,00	1,11	0,00	1,16	0,97	0,00	0,00	0,00	0,00	0,33	0,00	0,04	0,02
p.E1158X	p.E1158Q	0,00	0,07	-0,15	0,22	0,00	0,03	0,00	0,03	0,09	0,00	0,00	0,00	0,00	0,03	0,00	0,01	0,02
p.Q1160X	p.Q1160Y	0,17	5,03	0,95	2,03	0,72	2,39	1,43	1,95	1,45	1,05	1,09	0,77	0,70	0,01	-0,49	0,87	1,35
p.L1163X	p.L1163Y	0,33	11,44	1,14	2,06	0,29	-1,08	-0,19	-1,95	4,38	-0,99	2,23	0,03	0,06	4,62	0,34	1,57	1,81
p.E1165X	p.E1165Y	3,19	2,12	4,93	3,02	-0,41	-2,43	-1,17	-0,15	-7,05	-1,22	-2,77	3,36	4,28	-3,14	2,56	0,08	-0,04
p.K1166X	p.K1166Y	0,49	8,88	1,22	1,00	2,59	-1,59	2,90	-1,35	4,55	3,24	1,67	1,40	2,89	5,64	1,86	0,24	0,05
p.K1166X	p.K1166Q	0,03	-3,57	0,29	0,78	0,91	2,84	0,33	2,75	0,30	1,76	0,99	0,24	0,79	1,88	0,82	0,09	0,02
p.K1168X	p.K1168Y	-0,04	-10,76	-0,81	-1,81	-0,27	-3,70	-0,56	-4,28	-5,64	-1,18	-1,56	-0,33	-0,13	5,64	0,20	-0,18	0,05
p.K1168X	p.K1168Q	-0,16	-6,52	-1,31	-2,98	-0,37	-0,87	-1,06	0,51	0,21	-0,17	-0,86	-0,97	-0,96	0,47	0,27	0,09	0,02
p.G1172X	p.G1172W	0,26	0,00	1,31	0,00	1,12	3,49	2,35	2,80	-4,51	1,38	-2,75	0,00	0,85	-8,29	0,68	0,94	1,59
p.K1173X	p.K1173Y	0,19	1,95	0,98	2,13	0,76	2,67	1,50	-0,23	4,87	1,07	-0,83	0,36	0,77	13,97	0,61	0,78	1,26
p.E1175X	p.E1175Y	0,01	1,52	2,07	1,60	-1,12	-17,67	-0,21	-16,01	-10,24	-3,52	-5,07	0,36	1,99	16,73	-5,89	-6,13	11,61

p.W2131X	p.W2131Y	-0,50	-2,10	-0,57	-0,72	-1,67	-1,60	-2,11	-0,79	-9,53	-2,00	-0,93	-0,89	-0,27	-0,65	-1,16	0,01	0,99	-1,75	-0,65	-1,16	-2,73	-3,09	1,22	-0,23	0,53	-2,20	-9,53	1,22	-1,32	-0,91
p.Q2132X	p.Q2132Y	0,00	-2,24	0,00	0,00	2,87	-9,69	4,99	1,02	3,48	-0,52	0,43	-2,11	0,70	1,07	0,59	1,05	7,59	8,83	6,49	18,37	2,17	8,81	5,83	0,73	12,42	9,04	-9,69	18,37	3,15	1,06
p.R2135X	p.R2135W	8,93	3,36	9,52	8,37	2,53	9,34	-0,14	3,45	0,22	4,71	2,52	2,87	2,73	14,74	14,20	0,21	2,51	-6,28	-4,38	5,60	-8,76	8,87	2,11	2,85	2,95	-3,30	-8,76	14,74	3,45	2,86
p.G2136X	p.G2136W	10,21	0,00	-1,10	0,66	-5,76	6,27	-2,81	0,00	6,02	1,86	48,39	-0,35	-0,02	1,78	7,93	0,16	1,94	2,28	1,08	4,24	29,45	17,55	-12,53	19,31	-8,80	-8,59	-12,53	48,39	4,59	1,43
p.G2140X	p.G2140W	3,62	3,28	1,10	6,96	-1,82	9,62	2,37	7,82	16,95	4,70	1,42	0,15	0,45	0,18	7,38	6,30	17,17	5,18	2,95	16,32	-0,76	16,59	6,26	1,54	0,03	0,00	-1,82	17,17	5,22	3,45
p.L2142X	p.L2142W	-0,03	1,33	0,37	-3,65	0,00	0,29	0,43	1,83	7,75	0,40	0,19	3,50	1,65	0,45	-0,51	0,60	-7,33	-3,05	0,05	1,22	1,18	1,98	1,99	-0,02	-0,23	-0,09	-7,33	7,75	0,39	0,39
p.L2142X	p.L2142Y	-1,58	-21,08	-0,25	-5,18	0,00	-5,98	-2,86	-9,63	3,08	-2,16	-0,23	-2,06	0,96	-0,28	-5,04	-1,98	-5,60	-8,67	-0,05	-9,16	-1,09	-2,62	-3,77	-0,43	-1,74	-0,66	-21,08	3,08	-3,39	-2,02
p.L2142X	p.L2142Q	-2,78	-6,13	-1,04	0,43	0,00	-10,64	-0,04	-21,11	-1,59	-3,09	-0,84	11,31	0,15	-1,11	-6,66	-4,18	-8,43	-1,05	-0,24	-19,51	-3,77	-10,04	-8,78	-0,81	-4,59	-1,65	-21,11	11,31	-4,08	-2,21
p.S2151X	p.S2151Y	2,22	-9,99	1,60	3,40	1,52	-18,74	-7,58	-27,73	-20,87	-31,73	2,44	-18,11	0,82	0,19	-13,88	-0,13	-5,69	-2,65	-15,80	-6,77	0,00	-3,71	-16,06	-10,66	-7,71	0,24	-31,73	3,40	-7,91	-6,23
p.S2151X	p.S2151W	9,88	-4,04	2,87	6,49	3,89	-13,77	-6,35	-24,68	-17,82	-28,82	14,01	-15,59	0,91	8,83	-7,97	4,77	-5,74	5,56	-7,73	-3,46	0,00	7,83	-15,78	-9,22	-8,26	3,52	-28,82	14,01	-3,87	-3,75
p.S2151X	p.S2151Q	10,84	-1,34	-0,07	-0,83	13,78	-2,06	-0,67	-8,03	-2,58	-14,55	1,04	-4,28	-1,29	-0,41	2,16	-0,49	-0,66	-2,34	-7,72	-0,64	-0,09	-2,09	-8,83	7,38	-2,85	0,08	-14,55	13,78	-1,02	-0,75
p.K2155X	p.K2155Y	-8,66	4,30	0,49	-7,19	0,80	-28,74	-4,29	-19,12	-1,53	-9,07	-0,51	-9,31	-0,59	1,91	-8,29	-0,69	0,09	5,72	7,58	-2,51	-0,38	-6,50	-12,81	1,97	5,69	2,36	-28,74	7,58	-3,43	-0,64
p.K2155X	p.K2155Q	2,51	1,09	0,68	1,40	0,42	2,48	1,51	3,61	10,57	1,96	0,00	0,04	0,61	0,57	1,54	-0,93	-1,95	1,89	2,94	-3,64	-0,56	-10,68	-21,29	-3,83	1,69	-0,60	-21,29	10,57	-0,31	0,59
p.R2166X	p.R2166W	2,18	6,35	2,70	5,58	7,75	2,51	5,66	4,12	1,91	0,80	0,65	6,52	2,52	1,43	1,75	-2,17	6,21	5,39	11,40	7,74	-1,12	3,66	14,31	-1,57	-3,89	-12,22	-12,22	14,31	3,08	2,61
p.L2170X	p.L2170Y	9,41	-1,69	-0,57	-2,32	-3,92	-0,50	-3,21	-1,24	5,72	-0,20	-0,26	-1,74	-0,77	-0,41	1,91	-4,64	-2,00	-2,61	-4,66	-16,80	-12,21	15,04	-2,84	-0,05	-4,63	-15,44	-16,80	15,04	-1,95	-1,71
p.E2184X	p.E2184Y	7,74	6,84	-2,88	6,94	6,49	1,41	7,78	2,45	10,49	1,62	6,24	4,26	2,41	6,83	4,35	8,90	22,94	44,43	22,42	10,21	-7,36	8,45	2,21	0,00	8,94	10,09	-7,36	44,43	7,85	6,83
p.L2185X	p.L2185Y	-4,56	-9,12	-5,05	-4,71	-3,03	-0,69	-3,77	-1,54	-22,61	-1,07	-5,24	-2,46	-2,07	-3,82	-3,17	-4,79	3,74	3,29	-0,86	-1,84	-10,18	17,45	-2,55	-0,05	-22,32	-9,53	-22,61	17,45	-3,87	-3,10
p.C2188X	p.C2188W	5,43	-6,10	-3,24	-10,85	-9,30	-3,75	-8,77	-7,97	-8,50	-5,10	-46,82	-20,92	-18,83	12,07	-2,19	-13,47	-0,12	-0,18	-7,50	4,89	-52,95	-5,58	-11,08	-64,72	-13,02	-3,94	-64,72	12,07	-11,64	-7,73
p.L2190X	p.L2190W	-8,63	10,66	-14,98	2,48	-8,90	-26,79	-27,37	-24,35	13,27	-5,09	13,38	0,68	-27,51	6,65	-29,06	-6,55	-1,30	0,61	0,00	5,04	1,10	23,55	-15,48	-16,84	-32,99	-19,69	-32,99	23,55	-7,23	-5,82
p.L2190X	p.L2190Y	-20,26	7,17	-25,31	-6,43	-4,97	-34,09	-37,18	-32,20	3,09	-12,89	6,61	-3,34	-33,85	1,53	-33,05	-11,21	-5,78	-2,06	0,00	-3,81	-0,62	16,99	-6,61	-10,34	-26,59	-12,58	-37,18	16,99	-11,07	-6,52
p.L2190X	p.L2190Q	4,86	-8,54	4,83	-8,08	-0,28	5,08	-7,49	4,16	3,38	0,49	3,95	-7,02	8,81	-5,54	-1,06	19,37	-6,14	-5,61	0,00	13,78	-3,82	4,50	-9,80	10,67	6,78	29,99	-9,80	29,99	2,21	1,94
p.C2193X	p.C2193W	-4,62	0,00	-0,01	-2,78	-8,01	0,00	-2,90	0,00	-4,17	-0,04	-32,65	-0,01	-0,05	-2,48	-0,05	6,26	-5,83	-10,12	-2,24	25,42	-55,96	6,00	-16,54	-10,54	-16,24	-3,36	-55,96	25,42	-5,42	-2,63
p.L2197X	p.L2197Y	-13,73	6,28	-18,25	-29,27	-3,28	-2,03	-25,13	-1,98	7,45	-12,45	5,33	-16,40	-15,25	6,07	-14,18	-0,23	-24,30	0,00	0,00	-7,02	0,00	-3,18	-2,15	-6,13	-6,21	-15,20	-29,27	7,45	-7,36	-4,70
p.G2198X	p.G2198W	-9,81	-1,23	-12,01	-44,16	-32,36	-0,67	-42,30	-0,78	-14,83	-8,44	-11,73	-4,57	-11,89	-3,12	-9,35	7,02	-25,91	0,01	0,03	1,23	2,93	1,38	-5,33	14,96	-11,49	0,02	-44,16	14,96	-8,55	-4,95
p.E2200X	p.E2200Y	-8,29	3,37	-13,13	-33,32	-19,62	3,97	-28,04	4,14	-2,34	-11,49	-34,96	6,92	-7,23	0,00	-8,66	-1,76	3,84	2,48	0,61	3,22	2,15	0,00	0,16	0,20	1,07	0,00	-34,96	6,92	-5,26	0,00
p.K2202X	p.K2202Y	-27,90	4,48	-11,16	-30,85	-23,19	6,53	-33,56	10,61	11,62	-7,91	-36,30	-6,74	-18,45	3,06	-17,62	-3,68	-32,05	13,84	30,69	-10,24	-0,40	0,39	0,16	-9,61	-7,58	0,18	-36,30	30,69	-7,53	-7,16
p.K2202X	p.K2202Q	-1,22	1,83	2,07	-2,62	0,02	18,27	-1,08	22,71	9,66	39,97	-8,21	2,30	19,90	-3,09	13,01	23,60	3,62	38,38	39,84	0,74	0,21	0,08	0,07	3,40	1,12	0,10	-8,21	39,97	8,64	1,95
p.S2205X	p.S2205Y	-24,88	-3,11	-13,97	-35,33	4,76	0,83	-16,87	3,75	-35,39	-7,41	-10,55	-3,69	-15,60	-0,26	-14,46	0,01	0,01	15,65	9,52	-1,47	0,29	0,14	2,20	-3,73	-0,77	0,35	-35,39	15,65	-5,77	-1,12
p.S2205X	p.S2205W	-21,53	3,21	-12,97	-32,54	9,50	5,70	-2,31	10,64	-16,42	17,59	-3,16	4,12	-11,15	2,00	-2,99	-0,63	0,01	12,54	7,86	0,03	0,49	0,87	3,27	-2,77	-1,78	0,92	-32,54	17,59	-1,14	0,26
p.S2205X	p.S2205Q	-6,39	-0,92	-2,61	-9,01	-1,08	0,11	-5,38	-0,32	-6,00	-4,22	-1,23	2,57	7,01	-0,81	0,88	-4,10	0,00	7,05	6,23	-0,01	-0,48	0,01	-0,33	-3,25	4,32	0,07	-9,01	7,05	-0,68	-0,40
p.Q2208X	p.Q2208Y	-24,30	0,70	-8,73	-25,62	-9,25	0,87	-25,98	0,67	-7,13	3,60	-20,34	4,69	-7,49	6,99	-14,08	1,77	0,05	6,28	5,64	1,32	0,02	1,16	-0,84	-0,40	6,30	1,20	-25,98	6,99	-3,96	0,36
p.S2212X	p.S2212Y	1,53	0,55	-0,97	-3,36	9,07	-2,28	-13,93	-0,23	-34,25	-24,94	-43,53	1,78	3,48	19,57	3,84	2,91	0,26	1,04	0,74	1,70	0,54	13,47	3,84	-0,01	4,98	9,23	-43,53	19,57	-1,73	0,89
p.S2212X	p.S2212W	2,67	2,16	4,38	2,15	20,95	10,47	7,07	10,54	-5,01	-11,65	-39,81	5,22	6,59	44,71	6,93	4,01	0,46	2,43	1,94	2,91	1,60	20,85	9,80	-3,08	8,37	15,33	-39,81	44,71	5,08	4,19
p.S2212X	p.S2212Q	-0,24	-0,42	-0,61	-0,83	17,81	-1,47	3,36	-1,37	-14,29	-2,03	-9,14	-1,15	-0,66	3,35	-0,90	1,08	-0,04	-0,50	-0,31	-0,24	-0,32	0,57	-2,09	5,65	2,48	4,51	-14,29	17,81	0,08	-0,37
p.W2222X	p.W2222Y	-2,05	3,00	-0,50	-1,02	-2,59	1,58	-1,98	-0,33	5,93	3,83	-5,70	-4,30	-2,50	10,49	-2,07	-2,63	-0,40	-2,72	-5,67	-2,06	11,47	-3,67	23,86	0,60	7,84	1,06	-5,70	23,86	1,13	-0,76
p.S2225X	p.S2225Y	0,87	0,00	3,66	3,94	2,47	-3,50	6,11	-2,49	-16,31	-3,34	-4,83	1,53	3,05	0,00	-0,48	0,17	0,16	2,17	3,17	1,13	-1,37	9,61	3,98	-0,27	-2,12	2,57	-16,31	9,61	0,37	0,52
p.S2225X	p.S2225W	1,97	0,00	4,01	5,52	0,79	-1,58	7,57	0,00	-5,29	-2,05	-3,70	3,64	2,10	0,00	1,54	-0,16	0,35	4,00	6,65	2,32	-1,60	12,84	9,17	-0,13	-0,09	3,13	-5,29	12,84	1,96	1,16
p.S2225X	p.S2225Q	-0,22	0,00	-0,12	0,07	2,00	-1,34	4,96	0,00	-19,84	-3,88	-2,04	-0,69	-2,60	0,00	-0,92	-2,34	-0,06	-0,71	-1,07	-0,32	-2,57	3,71	-2,15	-0,35	-9,41	-2,95	-19,84	4,96	-1,65	-0,70
p.K2226X	p.K2226Y	2,16	5,08	3,85	3,71	3,87	3,50	1,31	1,62	-22,61	0,88	3,00	-7,31	3,33	2,07	-1,89	2,50	0,00	0,14	2,48	0,13	-0,90	-14,97	-7,61	1,00	1,29	2,35	-22,61	5,08	-0,42	1,46
p.K2226X	p.K2226Q	0,77	1,47	-0,15	0,40	1,46	0,99	-0,05	0,44	3,43	2,41	2,82	0,04	0,66	0,48	1,84	0,81	0,00	0,08	2,26	0,08	-0,55	-10,37	-3,76	0,30	-3,17	0,79	-10,37	3,43	0,13	0,46
p.R2228X	p.R2228W	-5,40	6,78	3,47	4,65	-3,83	3,36	-1,32	2,07	2,63	4,23	5,16	-14,23	-1,28	2,59	-0,37	3,08	5,40	-35,39	-20,01	8,14	3,75	9,37	-1,78	0,67	6,35	-4,82	-35,39	9,37	-0,64	2,61
p.W2238X	p.W2238Y	-9,52	-2,07	-2,70	-5,97	-17,80	-1,78	-15,71	-0,51	-5,28	-5,86	-15,41	-5,89	-2,46	-0,66	-0,60	-0,02	-12,84	-23,06	-12,58	-10,17	10,41	5,79	6,02	-0,10	7,23	12,82	-23,06	12,82	-4,18	-2,58
p.R2239X	p.R2239W	3,78	5,17	4,87	-1,10	-0,45	5,28	-0,65	1,81	10,48	6,27	13,74	8,71	3,45	2,36	0,63	0,05	-16,33	-26,27	-10,19	4,01	-3,11	16,67	5,45	2,08	10,06	22,29	-26,27	22,29	2,66	3,61
p.Q2241X	p.Q2241Y	4,82	-16,14	6,59	2,50	-17,53	-6,01	-16,59	-3,92	0,01	-8,55	-33,67	-15,76	-3,64	0,00	2,66	2,31	-20,40	0,00	-2,61	-29,44	2,47	5,01	3,00	0,30	1,24	6,91	-33,67	6,91	-5,25	0,00
p.K2246X	p.K2246Y	-9,27	1,60	-1,22	-8,81	-7,75	-3,76	-16,83	-4,42	-8,56	-3,34	-14,55	0,87	-0,76	-8,68	4,95	0,11	0,26	1,03	2,00	0,33	3,23	5,38	1,54	0,40	-16,60	0,96	-16,83	5,38	-3,15	-0,33
p.K2246X	p.K2246Q	-9,59	0,38	-4,46	-10,38	-0,29	-12,39	-3,63	-9,19	1,64	-1,60	-8,73	0,44	-2,58	-9,61	5,12	0,02	0,05	0,29	0,83	0,05	0,67	-6,13	0,30	0,12	-0,49	1,90	-12,39	5,12	-2,59	-0,13
p.E2247X	p.E2247Y	-5,28	1,20	1,26	-0,36	1,48	3,10	0,12	0,00	0,10	1,72	0,00	1,41	0,80	2,87	-0,22	0,08	0,21	7,28	1,91	0,26	-6,78	0,34	2,98	0,00	4,13	6,47	-6,78	7,28	0,97	0,57
p.W2248X	p.W2248Y	-3,24	-0,45	-0,84	-1,71	-6,64	0,00	-4,89	0,00	-3,41	-2,24	-2,85	-0,47	-0,82	-2,14	-0,16	-0,09	-0,16	-0,78	-1,76	-0,13	-1,77	-10,40	-7,93	-0,34	-3,43	-2,31	-10,40	0,00	-2,26	-1,74
p.Q2254X	p.Q2254Y	-14,79	10,72	-2,53	-5,70	-1,17	-0,46	-2,33	-0,28	0,59	-2,23	-11,80	9,46	-19,03	4,22	-14,21	1,62	0,77	1,20	1,48	1,14	-2,65	0,06	1,39	0,00	0,20	0,09	-19,03	10,72	-1,70	0,03
p.K2255X	p.K2255Y	0,45	0,78	4,47	5,38	0,00	-1,19	0,02	-0,44	0,04	-2,30	0,00	1,16	-9,62	6,35	-11,62	0,25	0,47	8,63	8,54	0,63	-4,21	0,01	0,29	0,00	0,04	0,05	-11,62	8,63	0,32	0,05
p.K2258X	p.K2258Y	-11,85	1,11	-6,30	-7,30	2,69	1,46	-4,30	4,05	8,29	8,60	6,26	1,22	-21,42	4,24	-15,64	0,09	0,20	1,21	2,14	0,27	5,64	0,83	4,28	1,94	0,09	-2,60	-21,42	8,60	-0,57	1,16
p.K2258X	p.K2258Q	5,21	0,36	3,26	4,38	0,91	10,66	-1,55	9,38	2,65	24,49	1,77	0,35	0,38	4,20	3,26	0,02	0,04	0,31	0,65	0,05	3,43	0,15	1,19	0,50	0,96	0,28	-1,55	24,49	2,97	0,93
p.G2261X	p.G2261W	-13,65	0,03	-0,19	2,23	-2,66	10,40	-3,12	11,21	0,00	13,03	-3,50	0,00	-20,50	-0,77	-16,51	0,00	0,00	-6,53	-3,28	-1,35	-14,47	8,06	18,74	3,68	7,67	8,60	-20,50	18,74	-0,11	0,00
p.Q2265X	p.Q2265Y	-0,77	-7,96	13,80	11,26	0,02	0,01	-0,24	0,00	0,35	-0,03	-5,99	-18,64	2,29	17,76	6,62	-12,84	0,02	0,00	1,33	1,58	-10,65	1,70	6,07	-2,19	6,75	4,17	-18,64	17,76	0,55	0,02
p.G2266X	p.G2266W	8,80	6,93	3,65	5,89	5,58	7,60	10,04	0,19	0,71	14,56	4,05	-5,31	11,17	5,78	9,05	-7,55	0,00	0,00	-2,51	2,58	4,89	1,71	1,26	8,93	7,16	5,73	-7,55	14,56	4,26	5,23
p.K2268X	p.K2268Y	-18,28	6,53	0,78	-5,57	-0,89	5,56	0,15	-9,34	0,71	-8,61	0,74	-17,25	-15,87	4,92	-20,49	0,00	2,21	2,89	8,57	8,84	5,02	0,80	7,07	2,65	3,25	2,71	-20,49	8,84	-1,27	0,79
p.K2268X	p.K2268Q	3,49	2,50	1,21	1,56	0,77	2,00	1,60	2,53	0,40	4,51	1,65	5,57	3,64	-0,62	4,13	0,00	0,40	0,59	1,60	1,03	2,47	0,21	1,80	0,72	-1,84	0,77	-1,84	5,57	1,63	1,48
p.K2277X	p.K2277Y	1,09	8,16	0,62	-0,58	1,47	5,60	1,60	0,28	0,36	7,70	10,48	7,00	1,37	2,89	-0,54	2,34	-0,61	6,89	0,25	-0,57	-2,87	4,98	4,43	8,39	8,01	7,71	-2,87	10,48	3,33	1,97
p.E2278X	p.E2278Y	0,25	4,80	0,01	-3,50	-0,75	0,14	0,63	0,22	0,26	3,47	-11,14	0,94	0,48	2,22	-0,63	0,22	0,16	8,62	5,87	4,06	-8,90	-10,13	10,92	-0,81	0,60	1,03	-11,14	10,92	0,34	0,25
p.Q2289X	p.Q2289Y	1,02	0,14	0,27	1,08	0,58	2,36	0,67	0,00	-22,06	0,01	3,67	0,00	0,47	1,37	0,79	-0,13	0,01	0,33	0,41	0,04	-1,81	0,15	3,06	-6,59	-9,15	0,00	-22,06	3,67	-0,90	0,21
p.W2290X	p.W2290Y	-1,01	-2,40	-0,23	-0,92	-0,51	-1,94	-0,57	-0,87	-5,04	-0,44	-1,29	-0,79	-0,60	-0,92	-0,32	-0,13	-0,01	-0,15	-0,12	-0,09	8,34	-0,92	-0,14	-2,78	-5,38	0,00	-5,38	8,34	-0,73	-0,54
p.Q2295X	p.Q2295Y	0,00	-3,07	0,00	0,00	0,00	-5,55	0,40	-1,30	0,00	-0,57	0,00	-0,86	-0,12	0,00	8,53	0,37	0,37	1,79	2,98	0,70	0,16	0,33	0,06	0,01	0,00	4,03	-5,55	8,53	0,32	0,00
p.K2298X	p.K2298Y	6,74	28,23	0,00	0,99	3,15	-9,82	1,47	-3,69	3,65	14,29	0,53	5,02	0,48	6,34	1,07	0,34	0,21	1,03	1,50	0,38	2,55	-0,60	-7,46	1,84	5,11	7,91	-9,82	28,23	2,74	1,27
p.K2298X	p.K2298Q	1,92	19,65	0,00	0,25	1,19	3,11	0,00	10,56	0,96	8,45	0,14	-1,28	-1,96	1,51	0,84	0,09	0,07	0,27	0,47	0,10	0,47	-0,55	0,94	0,31	0,99	0,80	-1,96	19,65	1,90	0,47
p.K2300X	p.K2300Y	-13,19	7,71	-4,42	-0,06	3,68	-6,91	-1,21	0,53	1,24	3,23	0,40	2,76	-1,24	-5,09	2,43	0,04	0,00	-0,59	0,45	0,10	-0,01	1,93	-2,41	0,79	-5,54	-2,04	-13,19	7,71	-0,67	0,02
p.Q2303X	p.Q2303Y	7,32	2,87	-0,13	0,00	0,00	2,69	1,43	1,47	2,63	0,77	0,25	1,15	-0,41	4,77	0,49	8,39	3,09	-12,36	-7,33	9,93	0,13	0,42	0,11	0,56	-5,23	2,18	-12,36	9,93	0,97	0,67
p.G2304X	p.G2304W	3,45	5,33	0,00	0,00	0,00	0,07	0,00	2,88	6,22	1,82	14,61	-2,14	-0,27	2,88	0,01	1,21	-18,46	-9,90	-5,28	-26,14	0,01	8,43	0,92	11,89	0,36	4,83	-26,14	14,61	0,11	0,21
p.L2320X	p.L2320Y	0,76	-10,33	-0,14	0,00	-0,15	-1,11	-0,24	-1,26	0,41	-5,41	0,00	-1,99	-1,10	-2,58	-1,19	-0,82	-1,60	-1,50	-0,08	-2,29	0,50	-0,82	-0,87	-5,44	0,25	-0,48	-10,33	0,76	-1,44	-0,84
p.L2320X	p.L2320W	4,40	9,66	-0,01	0,00	0,05	-0,07	0,06	-0,01	3,65	-0,36	0,00	0,14	0,08	-0,06	0,12	2,60	-3,23	1,32	-7,52	-4,30	2,88	1,53	-0,12	-3,70	3,29	2,28	-7,52	9,66	0,49	0,05
p.L2320X	p.L2320Q	0,24	-6,20	-0,23	0,00	-0,31	-1,95	-0,46	-2,31	-0,47	-2,84	0,00	-3,75	-1,92	-4,61	-2,20	8,02	-2,02	-3,59	-0,10	-5,98	-0,24	-3,84	-1,89	-4,02	-0,46	-3,32	-6,20	8,02	-1,71	-1,93
p.R2326X	p.R2326W	0,00	20,14	7,58	3,09	0,33	2,56	6,47	7,78	10,26	14,30	6,13	0,53	9,70	6,14	2,07	3,25	4,39	6,87	10,08	4,73	4,03	13,49	6,94	1,11	2,39	0,27	0,00	20,14	5,95	5,43
p.Q2330X	p.Q2330Y	0,68	21,46	4,91	4,61	-3,25	4,31	-3,26	0,60	-0,90	9,53	9,59	2,62	2,19	11,83	1,35	-3,24	0,23	6,21	7,31	-0,31	6,98	4,16	7,71	1,75	4,59	21,75	-3,26	21,75	4,75	4,24
p.W2332X	p.W2332Y	-0,70	-4,23	-9,45	-9,64	-2,48	-10,63	-4,41	-7,85	-3,85	-3,74	-2,23	-2,27	-2,93	-4,76	-1,82	-3,54	-1,94	-7,51	-9,71	-7,07	-6,05	-1,60	-2,27	0,26	-6,27	11,24	-10,63	11,24	-4,06	-3,80
p.Q2335X	p.Q2335Y	9,66	2,35	9,59	5,79	-1,14	0,70	2,17	8,09	-0,51	0,92	0,87	5,55	9,73	7,14	8,41	1,69	2,60	0,86	-11,81	5,49	-10,05	-1,26	-7,46	2,36	6,26	5,38	-11,81	9,73	2,44	2,35
