# Supplementary material for: Immunogenic implications of translational readthrough modulate the association of F8 nonsense mutations with inhibitors in Hemophilia A
Source: Mol Med. 2026 Jun 27;32:144. doi: 10.1186/s10020-026-01536-y (PMC13576119; doi:10.1186/s10020-026-01536-y)
Supplement: Supplementary file 5 [file 10020_2026_1536_MOESM5_ESM.pdf]

Supplementary data

Supplementary Table S2

Distribution of Pts^{inh} based on WT readthrough

A

WT Rdthr	*0*	*≥1*	*≥2*	*≥3*
+	164	31	10	0
(%)	*(20.1)*	*(13.4)*	*(6.0)*	*(0.0)*
-	653	200	156	136
Total	*817*	*231*	*166*	*136*

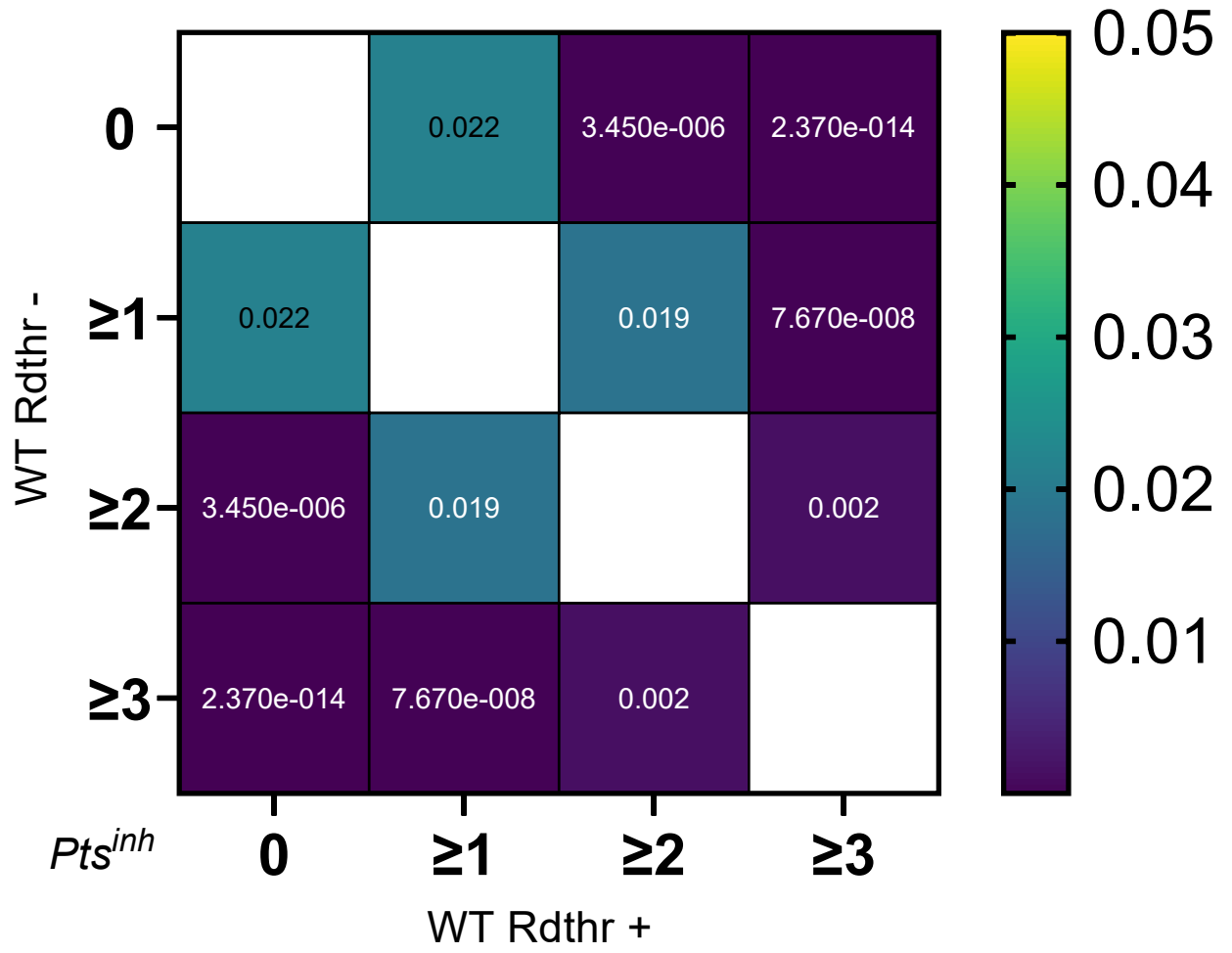

B

WT Rdthr	*0*	*≥1*	*≥2*	*≥3*
+	116	31	10	0
(%)	*(20.4)*	*(13.4)*	*(6.0)*	*(0.0)*
-	454	200	156	136
Total	*570*	*231*	*166*	*136*

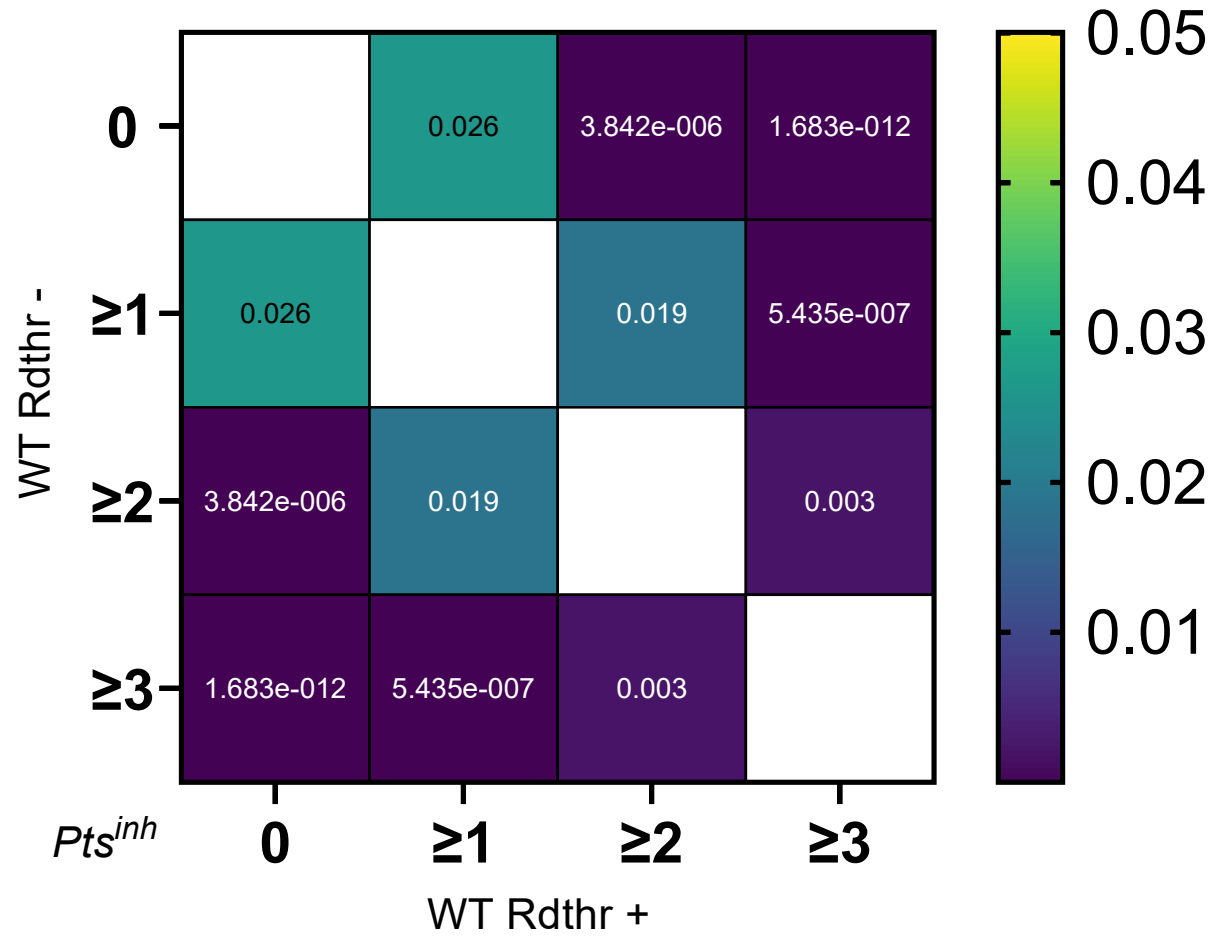

Data were obtained by the UAA=Tyr/Gln UGA= Trp and UAG=Tyr codification

A $Pts^{inh=0}$, all patients for whom inhibitor was not reported or was reported as negative;

B $Pts^{inh=0}$ only patients for whom the absence of inhibitor was reported in the database

Supplementary Table S3

Distribution of Pts^{inh} based on WT readthrough (UAG=Gln).

WT Rdthr	*0*	*≥1*	*≥2*	*≥3*
+	209	46	22	8
(%)	*(25.6)*	*(19.9)*	*(13.3)*	*(5.9)*
-	608	185	144	128
Total	*817*	*231*	*166*	*136*

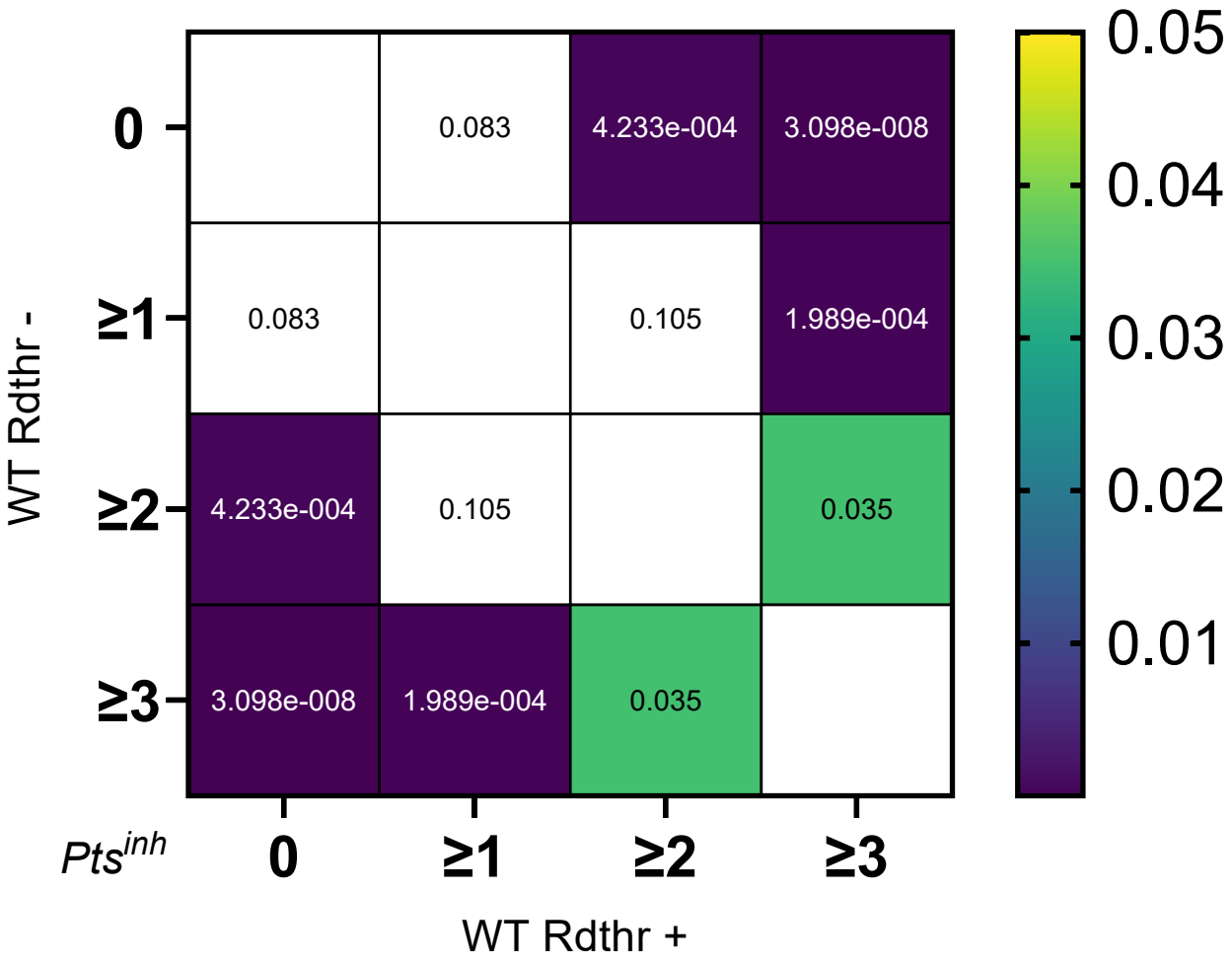

Data were obtained by the UAA=Tyr/Gln UGA= Trp and UAG=Gln codification

Supplementary Table S4

WT readthrough among $Pts^{inh=0}$ and $Pts^{inh\geq1}$ after implementation with CHAMP database data

WT Rdthr	*0*	*≥1*
+	159	35
(%)	*(19.7)*	*(14.5)*
-	648	206
Total	*807*	*241*

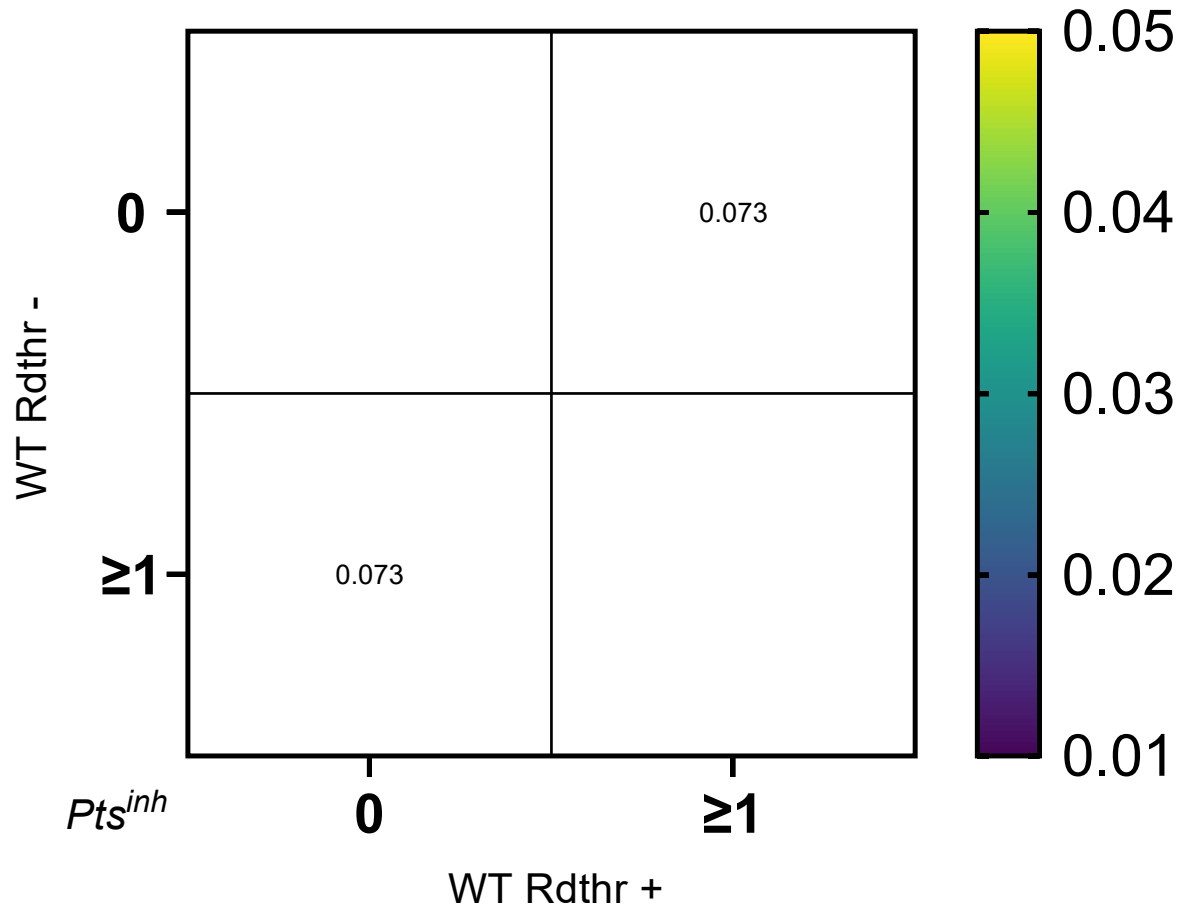

Supplementary Table S5

Distribution of Pts^{inh} for each PTC class.

Inh	*UGA*	*UAG*	*UAA*	*Total*
+	*146*	*59*	*26*	231
(%)	*(23.8)*	*(24.3)*	*(13.5)*	
-	*467*	*184*	*166*	817
Total	*613*	*243*	*192*	1048

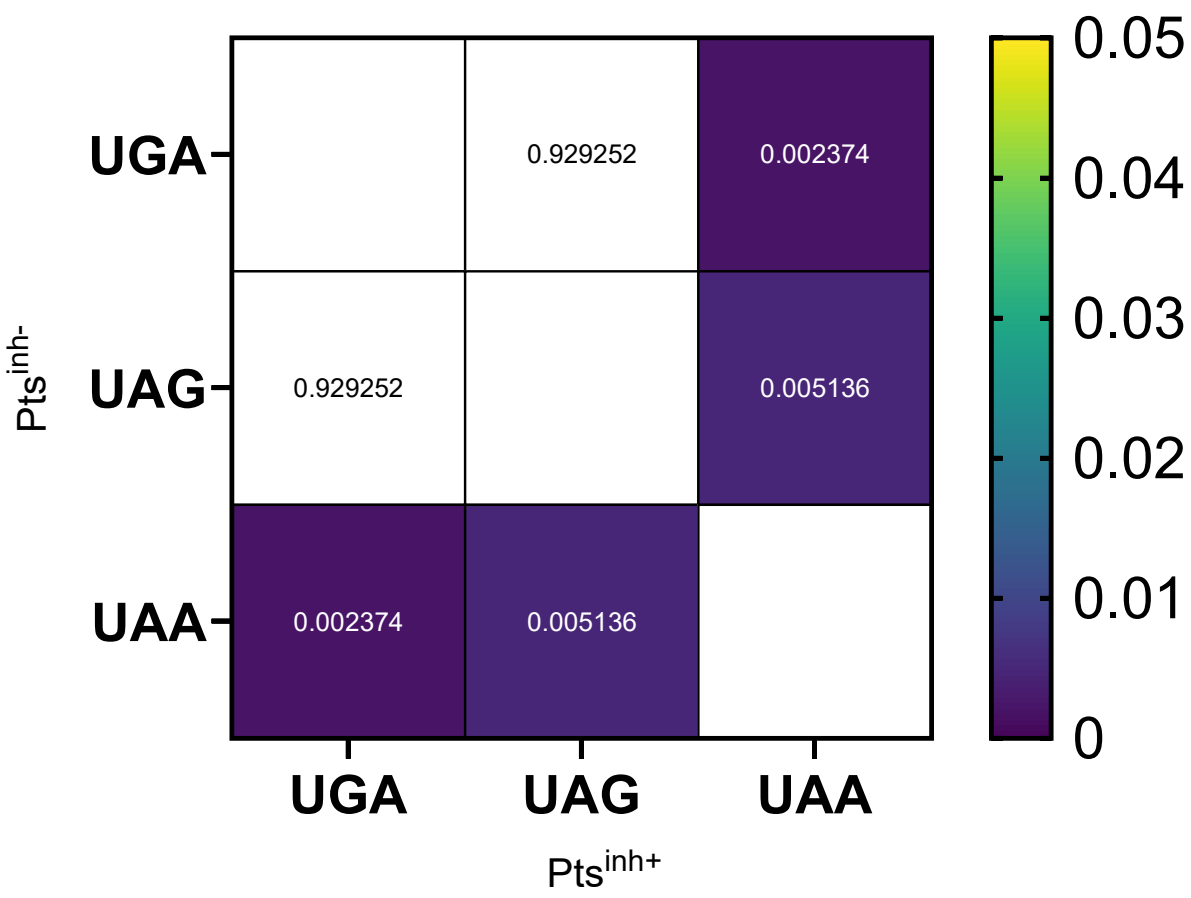

Supplementary Table S6

Distribution for each PTC class of Pts with PTCs undergoing WT Rdthr

WT Rdthr	*UGA*	*UAG*	*UAA*	*Total*
+	*69*	*28*	*98*	195
(%)	*(11.2)*	*(11.5)*	*(51.0)*	
Total	*613*	*243*	*192*	1048

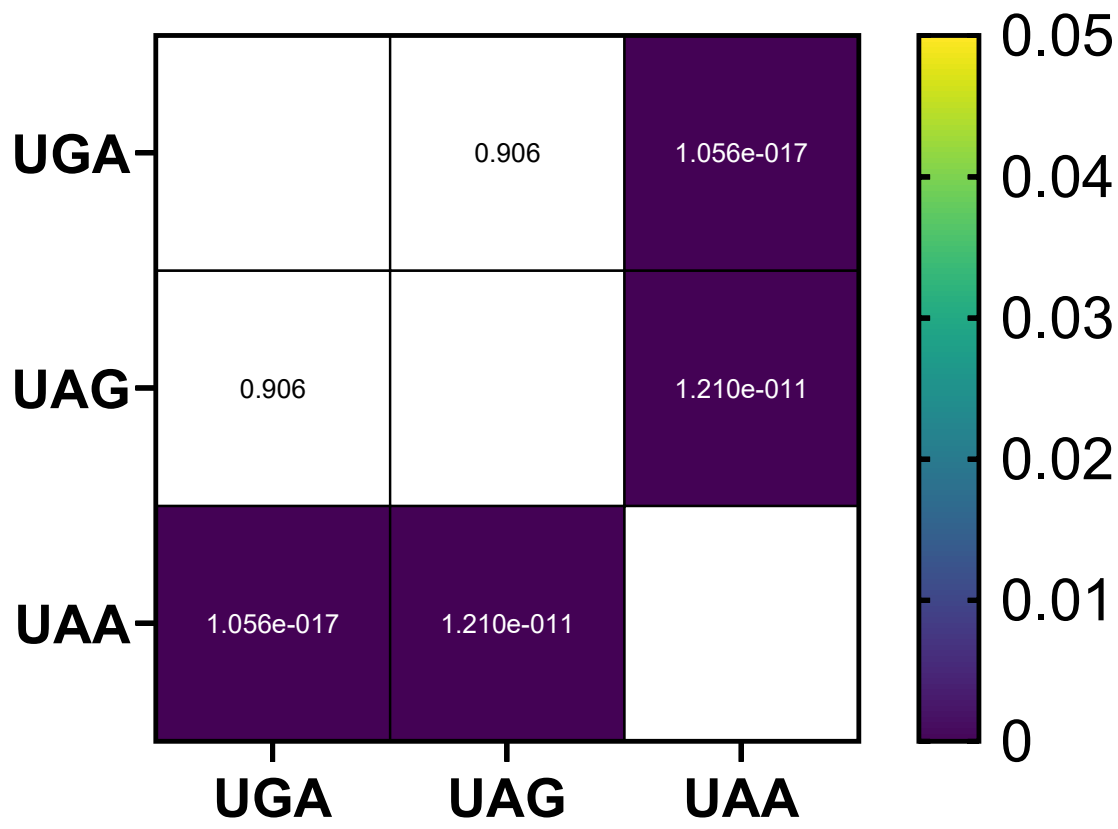

Supplementary Table S7

Distribution of Pts^{inh} for each tetranucleotide class.

Inh	UGAC	UGAA	UGAU	UGAG	UAGU	UAGC	UAGA	UAGG	UAAC	UAAG	UAAU	UAAA	Total
+	37	46	46	17	12	11	17	19	3	10	4	9	231
(%)	(24)	(23)	(27)	(19)	(22)	(35)	(21)	(24)	(10)	(15)	(16)	(12)	
-	118	155	124	70	42	20	63	59	26	55	21	64	817
Total	155	201	170	87	54	31	80	78	29	65	25	73	1048

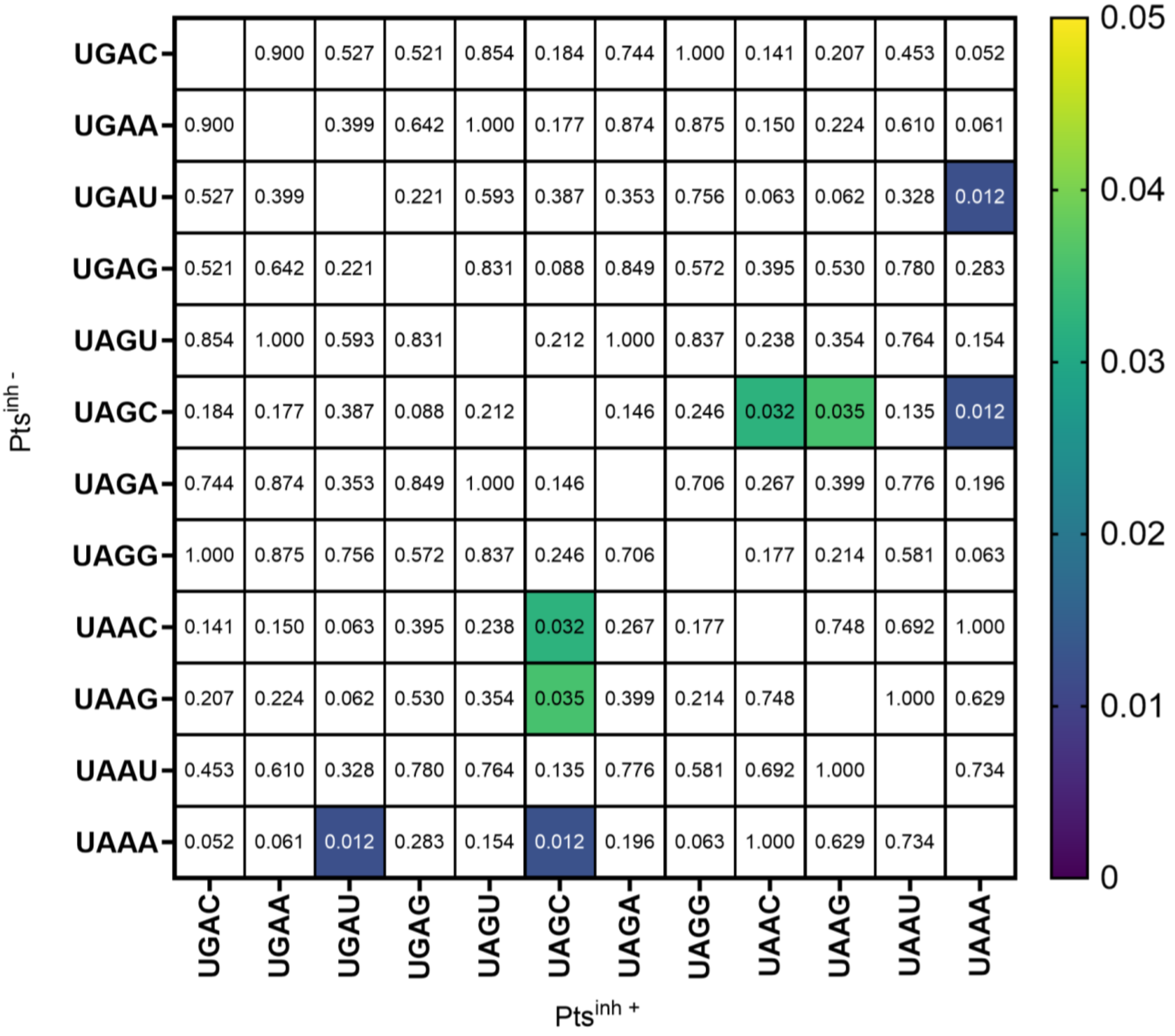

Supplementary Table S8

Distribution of Pts with WT Rdthr for each tetranucleotide class.

WT Rdthr	UGAC	UGAA	UGAU	UGAG	UAGU	UAGC	UAGA	UAGG	UAAC	UAAG	UAAU	UAAA	Total
+	2	3	1	3	3	4	0	1	3	4	2	5	31
(%)	(5.4)	(6.5)	(2.2)	(18)	(25)	(36)	(0.0)	(5.3)	(100)	(40)	(50)	(55.6)	
-	35	43	45	14	9	7	17	18	0	6	2	4	200
Total	37	46	46	17	12	11	17	19	3	10	4	9	231

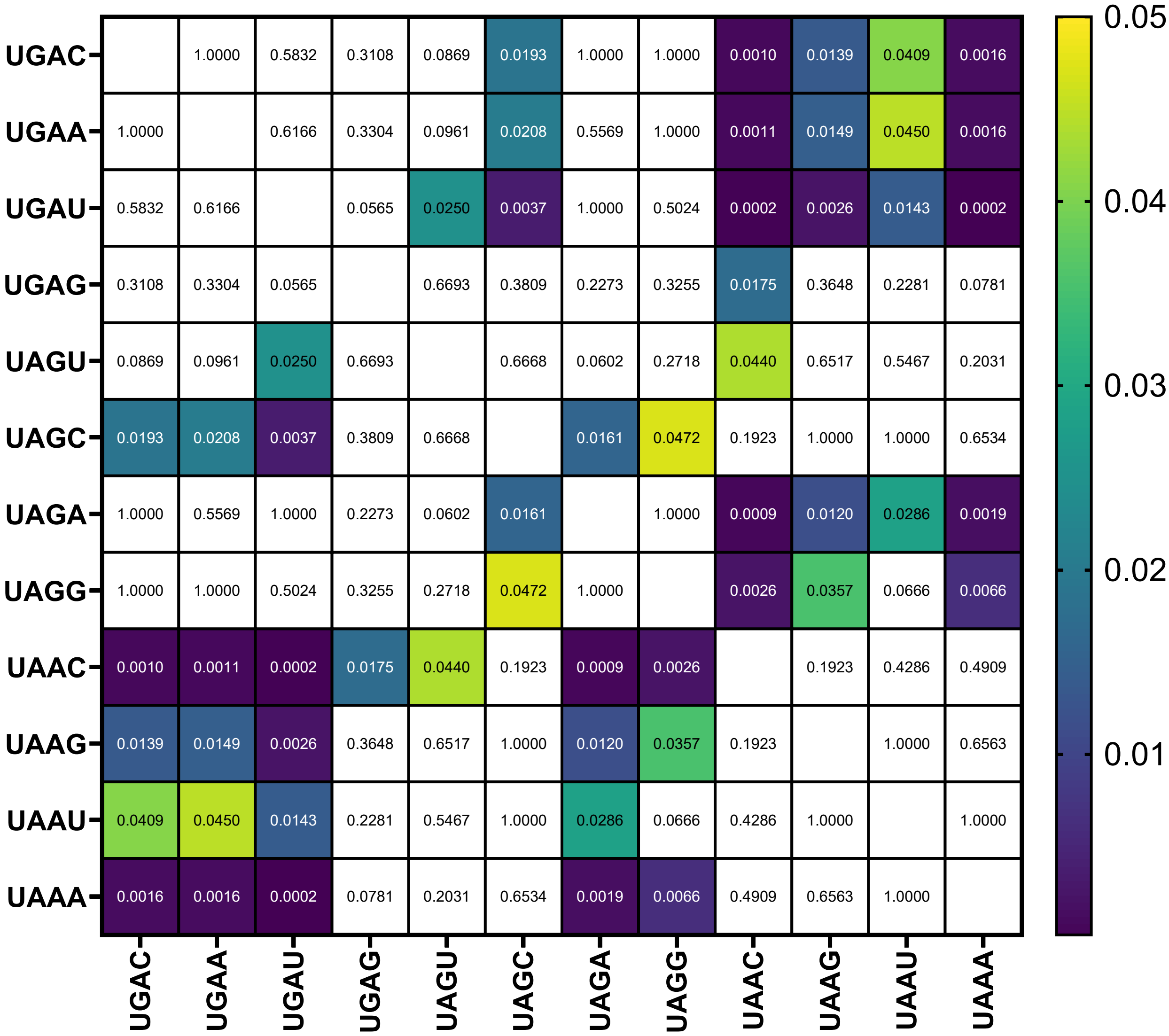

Supplementary Table S9

Distribution of Pts^{inh} for domain localization of PTCs and for PTC classes.

	All PTC				*UGA*			*UAG*			*UAA*		
Inh	*A1-A2*	*B*	*A3-C1-C2*	*Total*	*A1-A2*	*B*	*A3-C1-C2*	*A1-A2*	*B*	*A3-C1-C2*	*A1-A2*	*B*	*A3-C1-C2*
+	*42*	*34*	*157*	*230*	*24*	*15*	*106*	*14*	*10*	*35*	*4*	*9*	*13*
(%)	*(14.7)*	*(12.9)*	*(32.1)*	*(22.4)*	*(14.8)*	*(15.6)*	*(31.6)*	*(17.9)*	*(15.9)*	*(34.3)*	*(8.7)*	*(8.6)*	*(30.9)*
-	*244*	*229*	*322*	*795*	*138*	*81*	*229*	*64*	*53*	*67*	*42*	*95*	*29*
Total	*286*	*263*	*479*	*1028*	*162*	*96*	*335*	*78*	*63*	*102*	*46*	*104*	*42*

Supplementary Table S10

Distribution of Pts WT Rdthr based on FVIII domain localization of PTCs.

WT Rdthr	*A1-A2*	*B*	*A3-C1-C2*	*Total*
+	*72*	*72*	*51*	195
(%)	*(25.2)*	*(27.4)*	*(10.6)*	
-	*214*	*191*	*428*	833
Total	*286*	*263*	*479*	1028

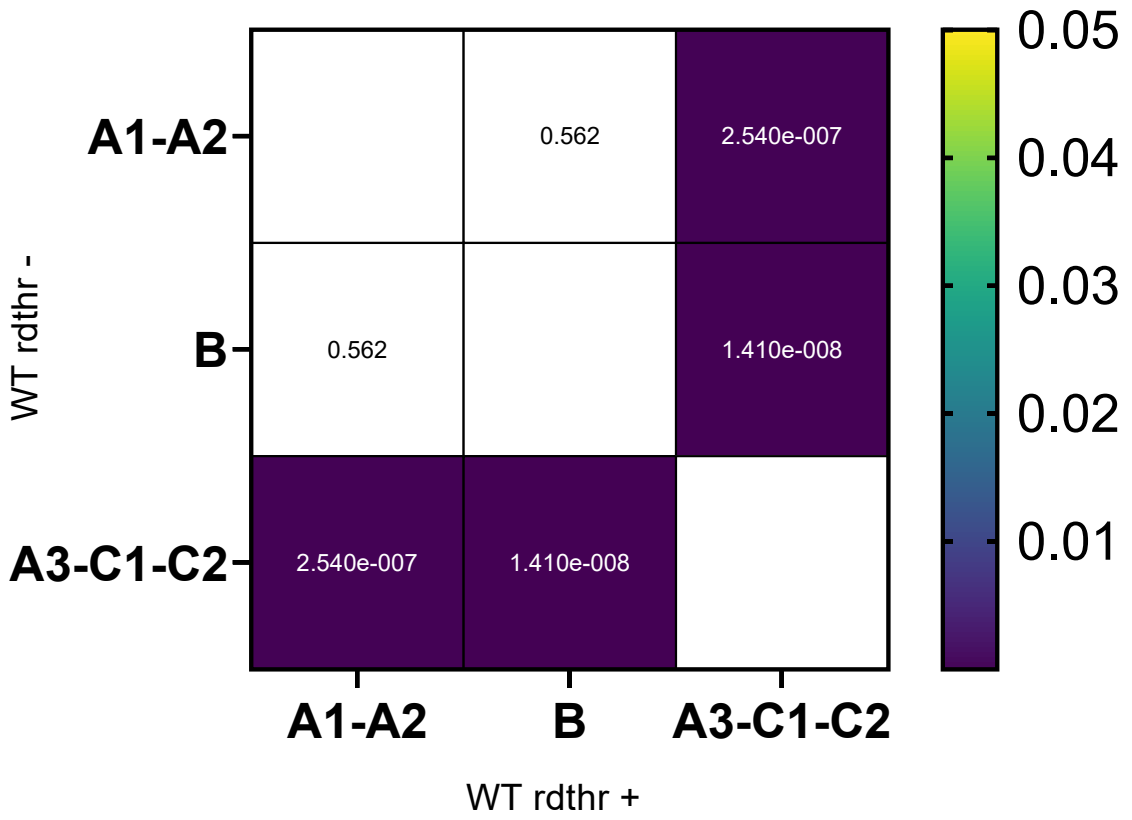

Supplementary Table S11

Distribution of WT Rdthr for PTCs observed (EAHAD) and predicted (never reported) as single nucleotide variants.

WT Rdthr	*Observed*	*Predicted*	*Total*
+	*105*	*144*	*249*
(%)	*(31)*	*(22)*	*(25)*
-	*230*	*518*	*748*
Total	*335*	*662*	*997*

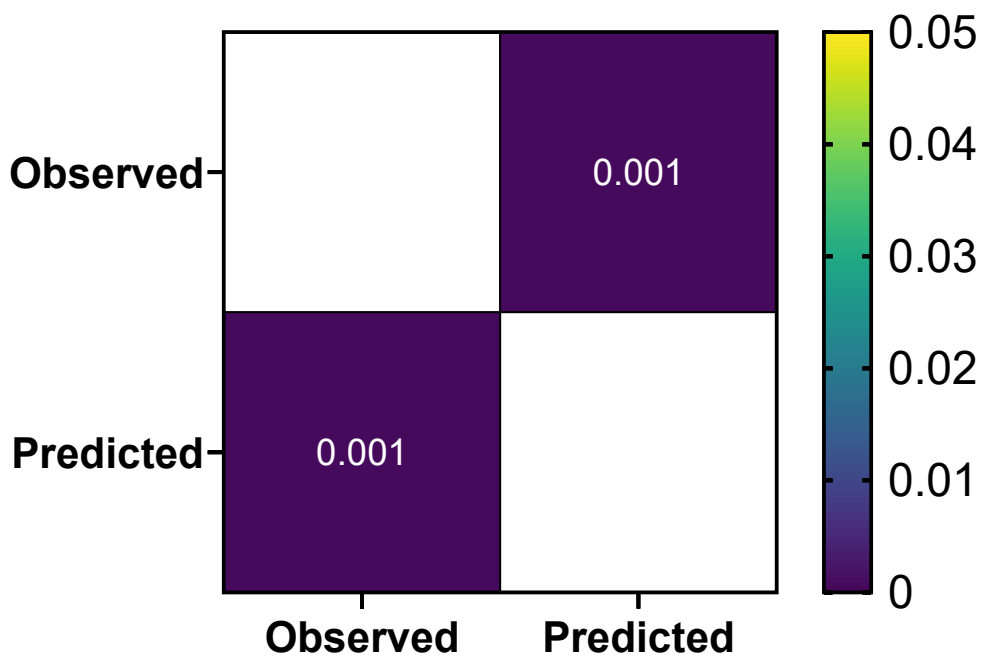

Supplementary Table S12

Distribution for each PTC class of PTCs observed (EAHAD) and predicted (never reported) as single nucleotide variants.

	Observed n(%)	*Predicted n(%)*	*Total n(%)*
UGA	*88(27)*	*160(24)*	*248(25)*
UAG	*122(36)*	*223(34)*	*345(35)*
UAA	*125(37)*	*279(42)*	*404(41)*
Total	*335*	*662*	*997*

Supplementary Table S14

Mean differential affinity, expressed as percentile ranking, of peptides obtained from WT Rdthr- PTCs grouped by PTC^{inh} status and FVIII domain location.

	A1-A2			*B*			*A3-C1-C2*		
PTC^{inh}	0	≥1	≥2	0	≥1	≥2	0	≥1	≥2
**Mean**	0,53	0,80	3,00	-2,25	-0,55	1,32	-0,48	-0,04	0,34
**Standard error**	0,16	0,37	0,66	0,19	0,29	0,86	0,23	0,21	0,38
**Count**	1768	364	156	2574	728	104	1144	1118	442
**Variants number**	68	14	6	99	28	4	44	43	17

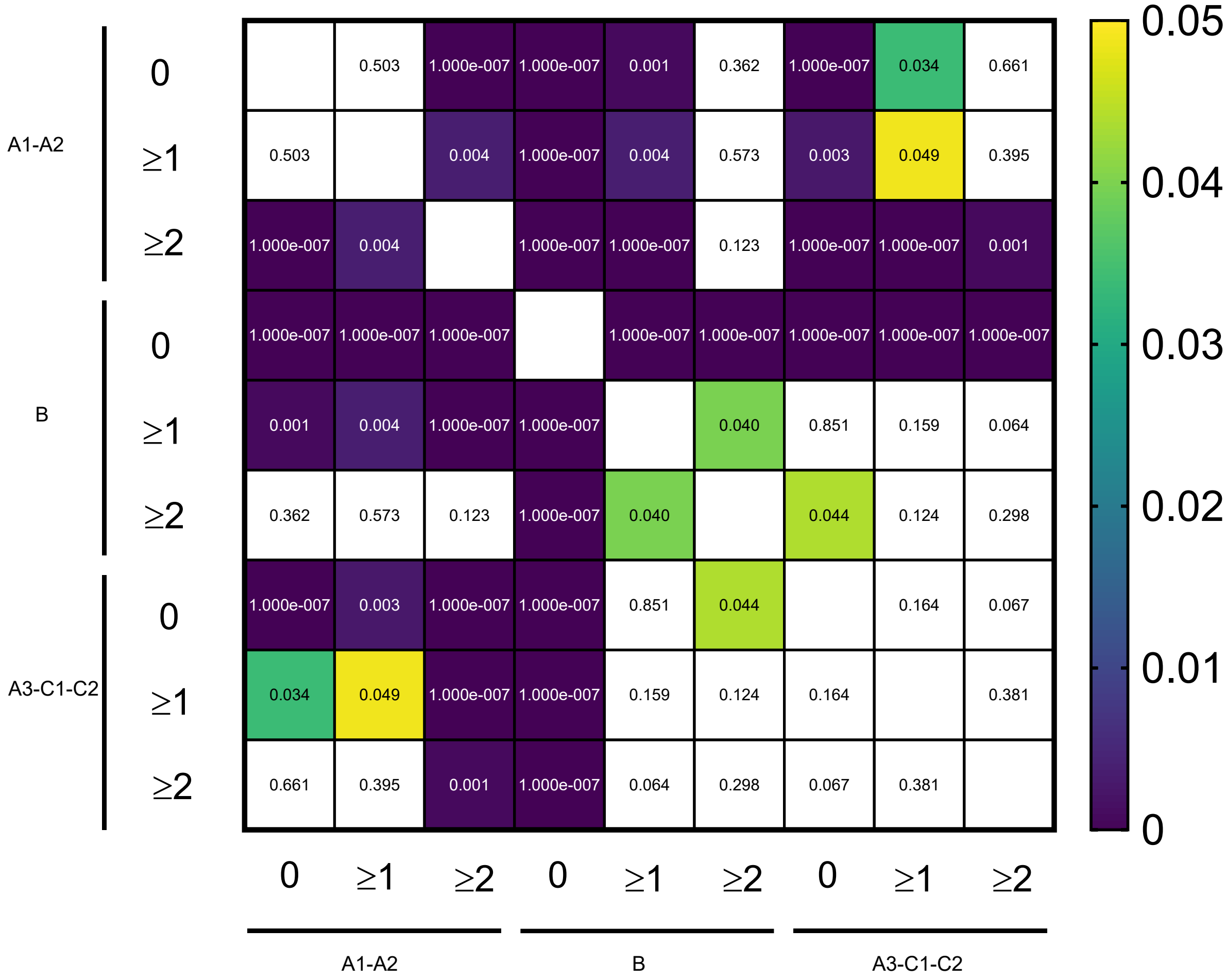

Supplementary Figure S1

A

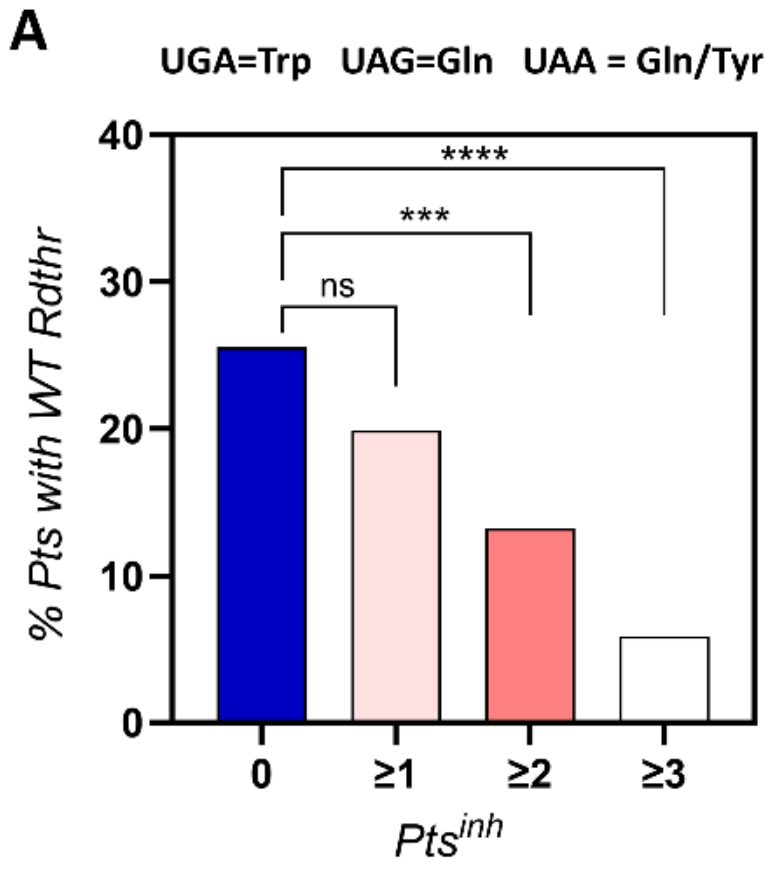

B

	EAHAD (UAG=Tyr)	EAHAD (UAG=Gln)
0 vs ≥1	2,16E-02	8,25E-02
0 vs ≥2	3,45E-06	4,23E-04
0 vs ≥3	1,11E-12	3,10E-08
≥2 vs ≥3	2,50E-03	3,50E-02
≥1 vs ≥3	5,44E-07	1,99E-04

Insertion of WT amino acid upon readthrough in the UAG=Gln model

A) HA patients with *F8* PTCs predicted to undergo WT readthrough, exploring Gln at UAG, were classified in accordance with PTCs associated with an increasing number of inhibitor-positive cases ($Pts^{inh=0}$, patients with PTCs never found in patients with inhibitors; $Pts^{inh\geq1}$,$Pts^{inh\geq2}$ $Pts^{inh\geq3}$, patients affected by PTCs found in 1,2,3 or more patients with inhibitors). B) Statistical comparison between models with Tyr or Gln reinsertion at UAG. Statistical significance of Fisher's exact test.

Supplementary Figure S2

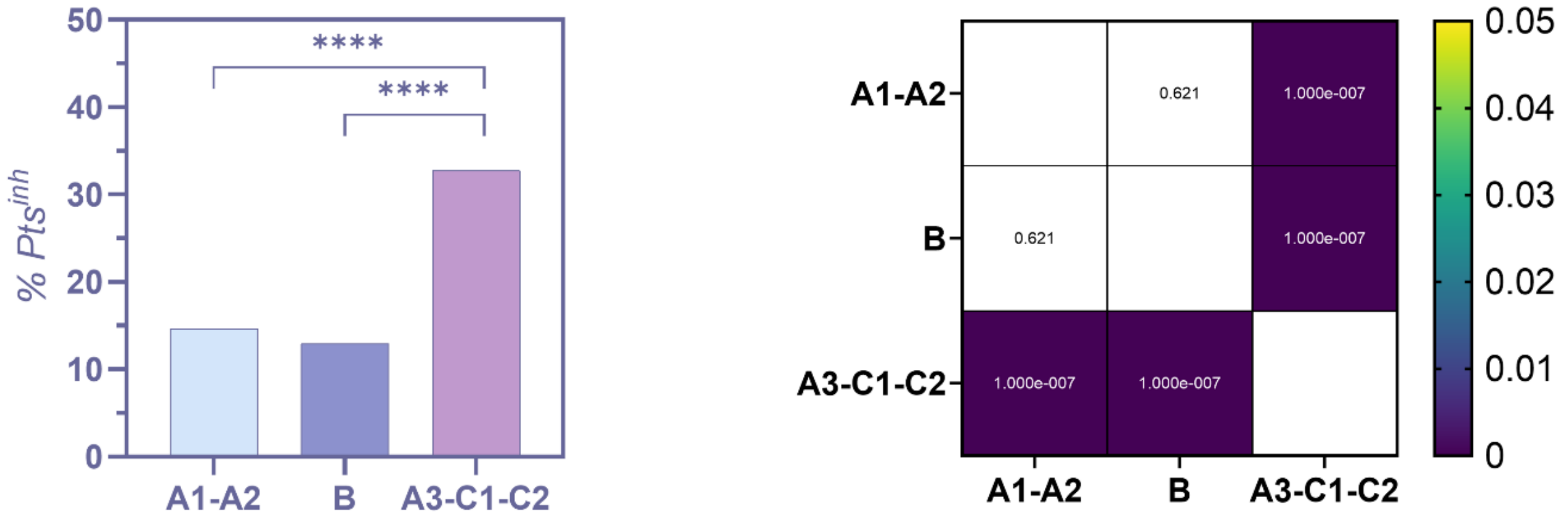

Comparison of the percentage of Pts^{inh} with PTCs in FVIII domains.

Left panel. Distribution of Pts^{inh} with PTCs in grouped FVIII domains. *Right panel*. Statistical Comparison

Supplementary Figure S3

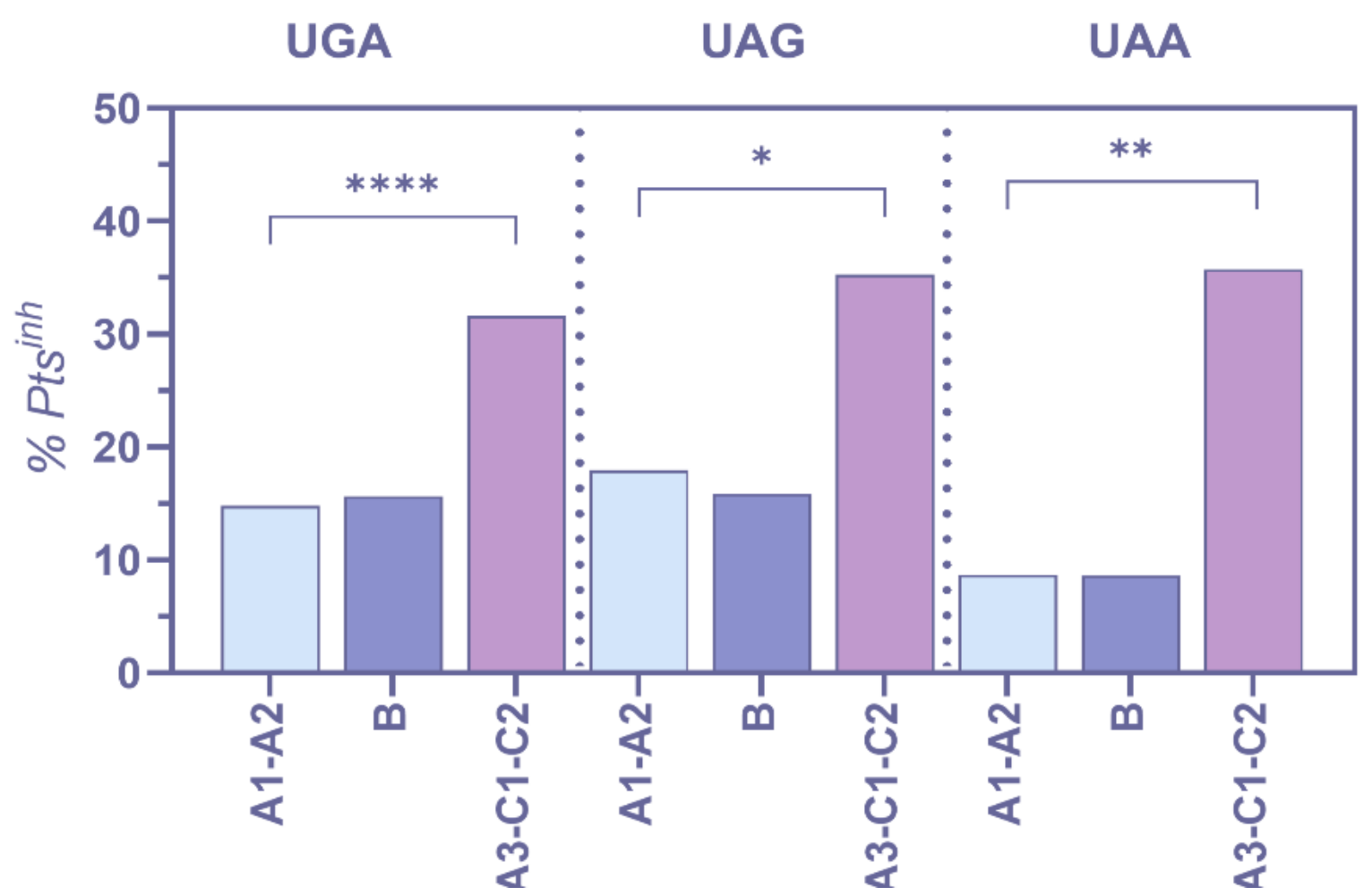

Comparison of the percentage of Pts^{inh} for PTCs in FVIII domains.

Distribution of Pts^{inh} in relation to FVIII domain location of PTC classes (UGA/UAG/UAA).

Supplementary Figure S4

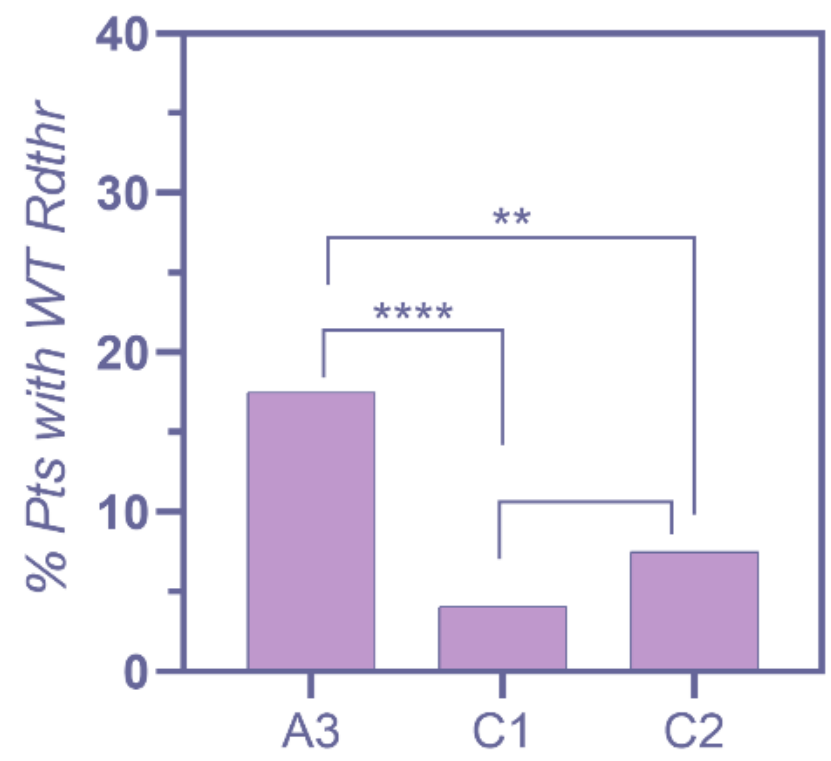

Comparison of the percentage of Pts with PTCs with WT Rdthr in single FVIII domains (A3, C1 and C2)

Supplementary Figure S5

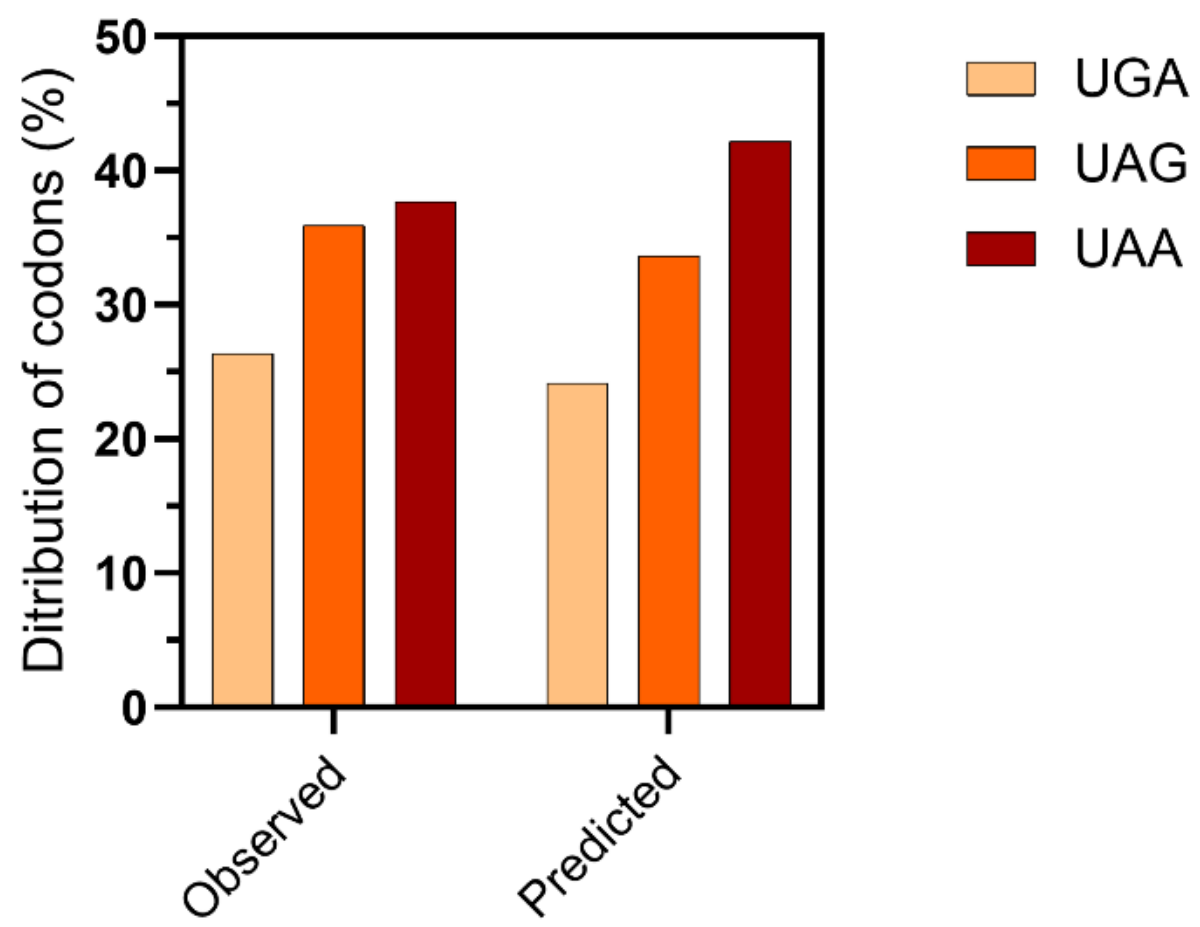

Comparison of observed (EAHAD) and predicted (never reported before) PTCs for each PTC class (UGA, UAG, UAA).

Prediction of PTCs produced by single nucleotide variants (SNV).

Supplementary Figure 6

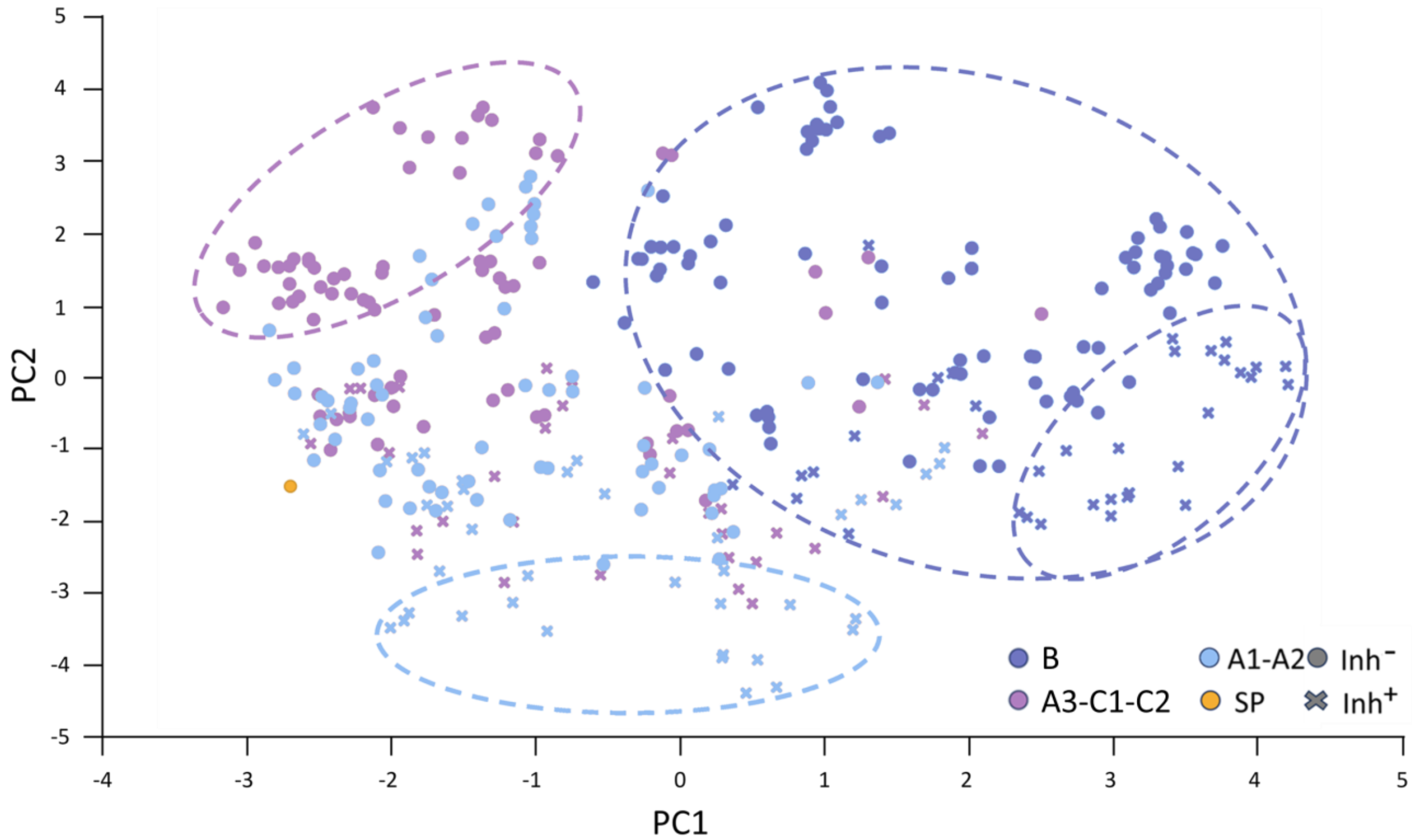

Distribution of observed PTCs by PCA

Grouping of observed (EAHAD) PTCs by Principal Component Analysis (PCA) including as variables PTC class, fourth nucleotide, *F8* PTC exon location, FVIII protein domains, prediction of WT readthrough and inhibitor association. Each PTC was color-coded based on its localization within specific FVIII domains (A3-C1-C2, purple; A1-A2, light blue; B domain, violet; Signal Peptide, SP, orange) and symbol-coded according to inhibitor association (dot, inh$^-$; cross, inh$^+$). Subgroups of PTCs are highlighted by dashed, colored ovals. Small subgroups of inhibitor-positive PTCs are underlined in the B domain and A1-A2 chains, and an inhibitor negative subgroup in the A3-C1-C2. To enhance data visualization and prevent overlapping among points, jittering was applied to the plotted data.

Supplementary Figure S7

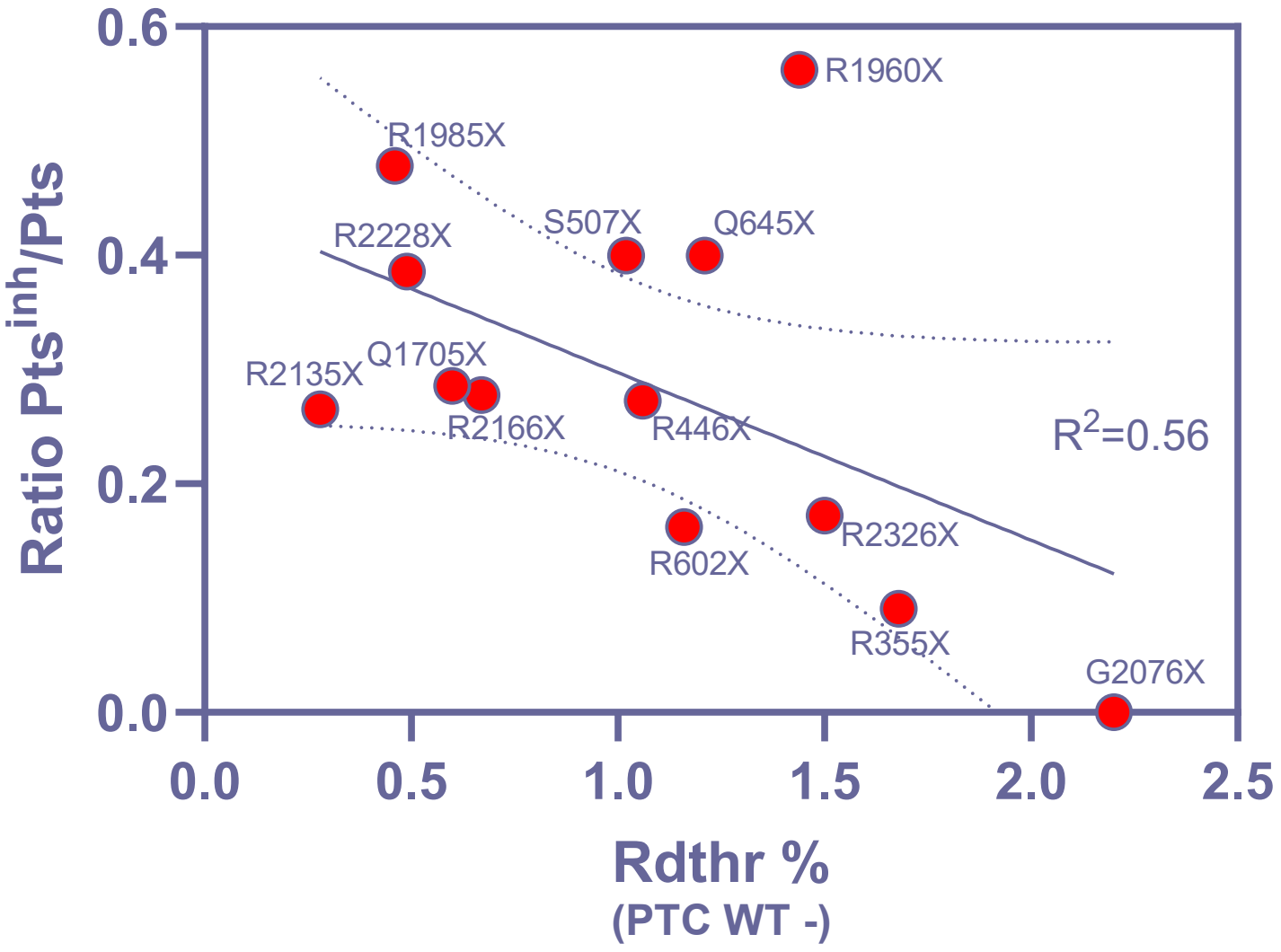

Correlation between recombinant Rdthr output and ratio of patients with inhibitor on total number of patients for WT Rdthr- PTCs.

Inverse relation between readthrough over PTCs WT –, excluding those within B domain, and ratio of patients with inhibitor on total number of patients for 12 PTCs WT – (red dots). Among the recombinant PTCs variants, the R1960X PTC value (Rdthr % 1.44), located completely outside the 95% (or 99%) confidence limits of the relation between the experimental readthrough and patients with inhibitor/total number of patients ratio, is reported but not considered to estimate the displayed R^2 value. If included, the R^2 is 0.22.
